# Supplementary material for: New Genetic Bomb Trigger: Design, Synthesis, Molecular Dynamics Simulation, and Biological Evaluation of Novel BIBR1532-Related Analogs Targeting Telomerase against Non-Small Cell Lung Cancer
Source: Pharmaceuticals (Basel). 2022 Apr 14;15(4):481. doi: 10.3390/ph15040481 (PMC9025901; doi:10.3390/ph15040481)
Supplement: Supplementary file 1 [file pharmaceuticals-15-00481-s001.zip › pharmaceuticals-1664622-supplementary.pdf]

New Genetic Bomb Trigger: Design, Synthesis, Molecular Dynamics Simulation, and Biological Evaluation of Novel BIBR1532-Related Analogs Targeting Telomerase against Non-Small Cell Lung Cancer

Haytham O. Tawfik [1,*], Anwar A. El-Hamaky [1], Eman A. El-Bastawissy [1], Kirill A. Shcherbakov [2], Alexander V. Veselovsky [2], Yulia A. Gladilina [2], Dmitry D. Zhdanov [2,3,*], and Mervat H. El-Hamamsy [1]

[1] Department of Pharmaceutical Chemistry, Faculty of Pharmacy, Tanta University, Tanta 31527, Egypt; anwaramin@pharm.tanta.edu.eg (A.A.E.-H.); emanesmat@pharm.tanta.edu.eg (E.A.E.-B.); mhamamsy@pharm.tanta.edu.eg (M.H.E.-H.)
[2] Laboratory of Medical Biotechnology, Institute of Biomedical Chemistry, Pogodinskaya St. 10/8, Moscow 119121, Russia; kirill.soff@gmail.com (K.A.S.); veselov@ibmh.msk.su (A.V.V.); leonova_y@mail.ru (Y.A.G.)
[3] Department of Biochemistry, Peoples' Friendship University of Russia (RUDN University), Miklukho—Maklaya St. 6, Moscow 117198, Russia
* Correspondence: haytham.omar.mahmoud@pharm.tanta.edu.eg (H.O.T.); zhdanovdd@gmail.com (D.D.Z.)

Supplementary Material

Spectral data section ……………………………..…. S2-S54
Biological section ……………………………….…. S55-S59

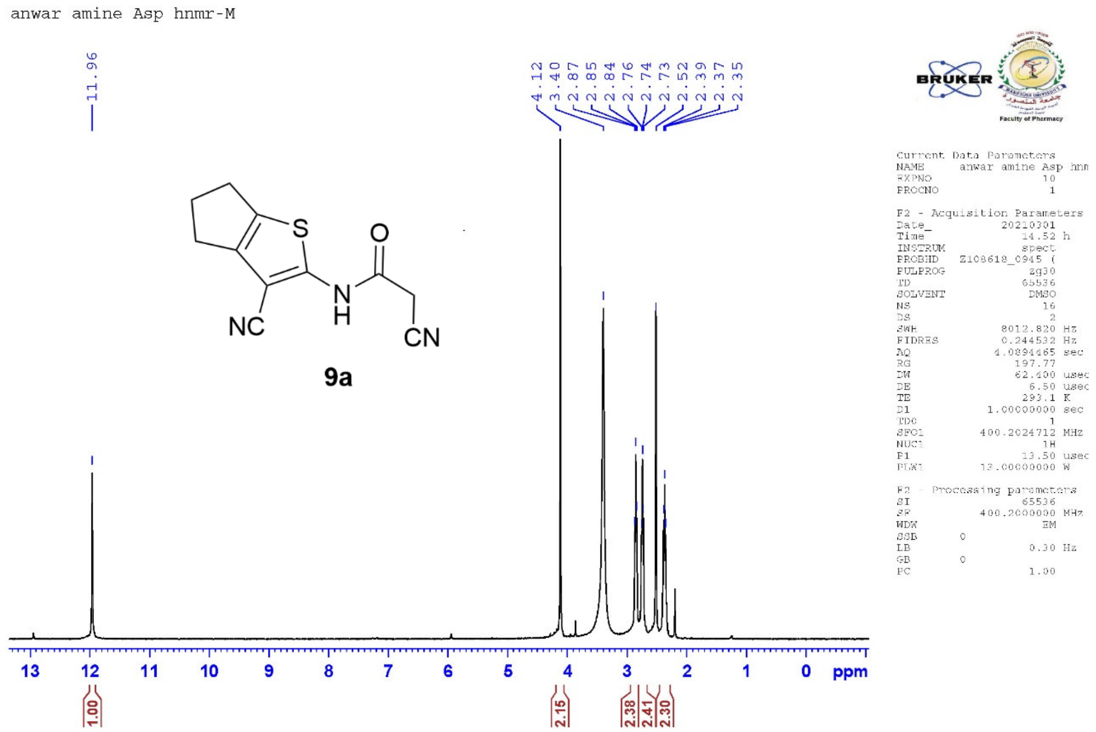

Figure S1. ^{1}H NMR (400 MHz, DMSO-d_6) spectrum of compound **9a**.

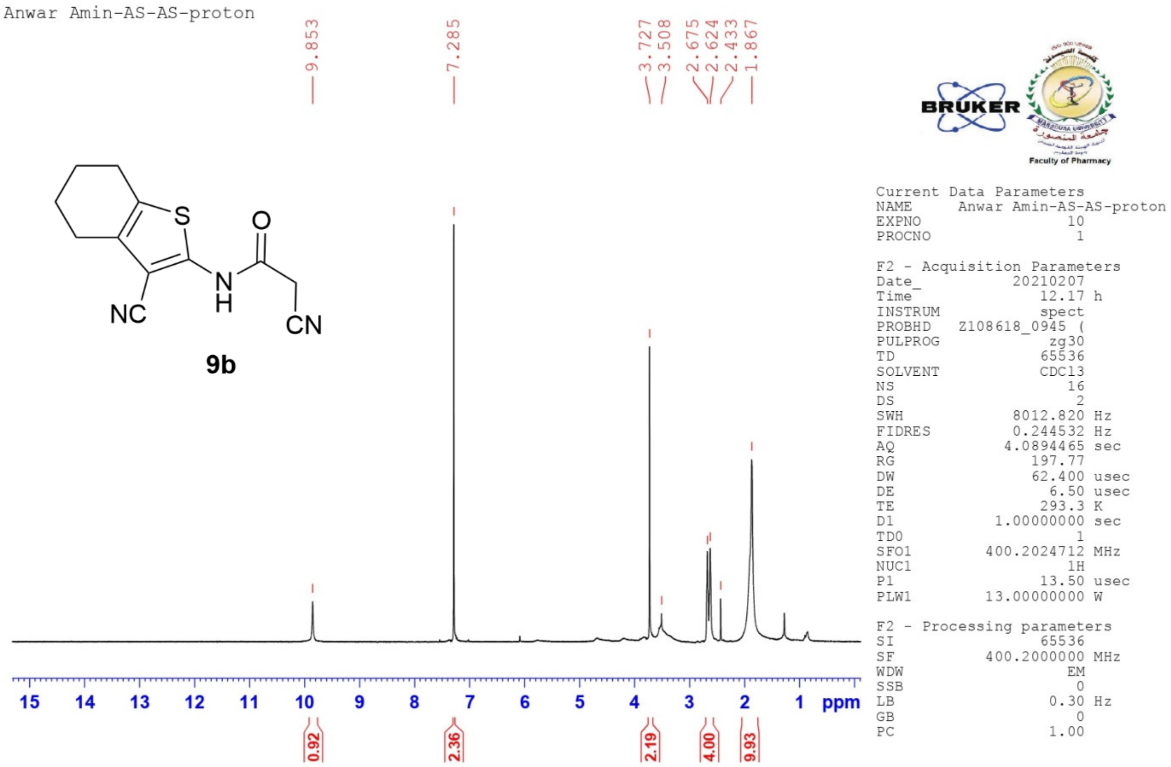

Figure S2. ^{1}H NMR (400 MHz, $CDCl_3$) spectrum of compound **9b**.

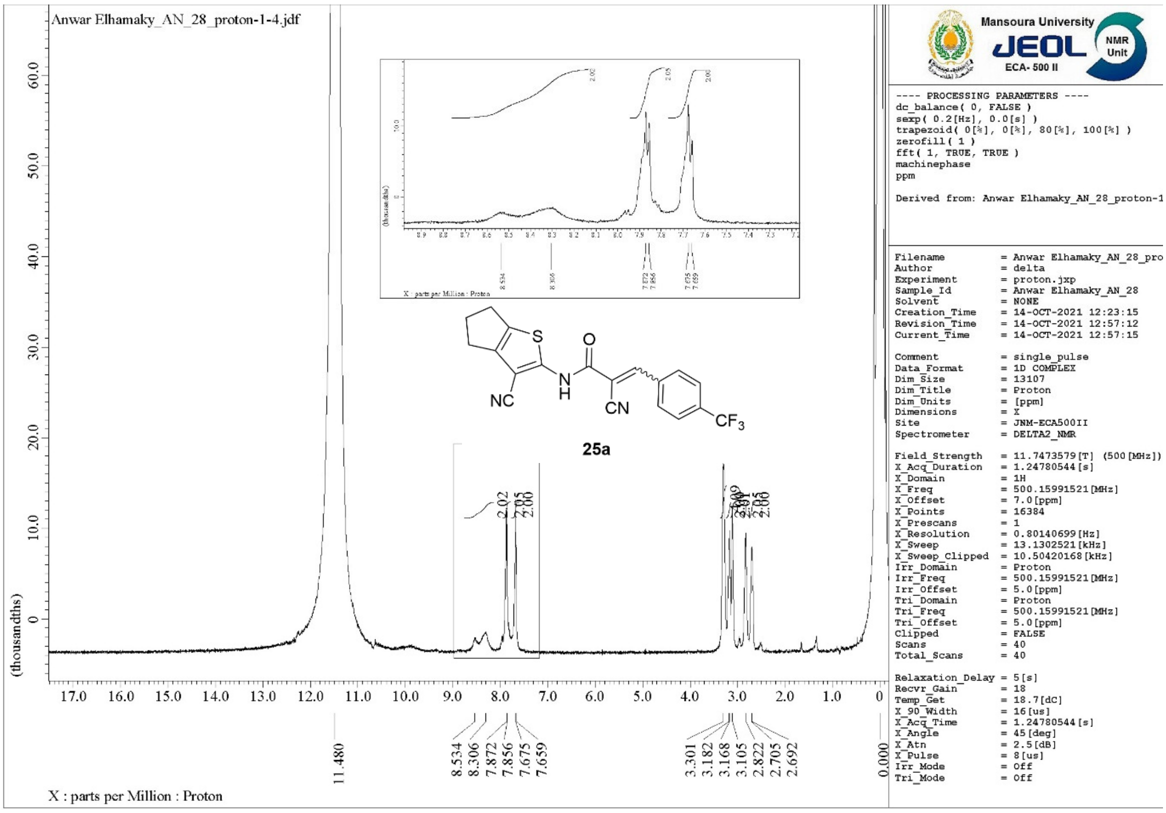

Figure S3. ^{1}H NMR (500 MHz, CF_3COOD) spectrum of compound **25a** as *E*/*Z* mixture.

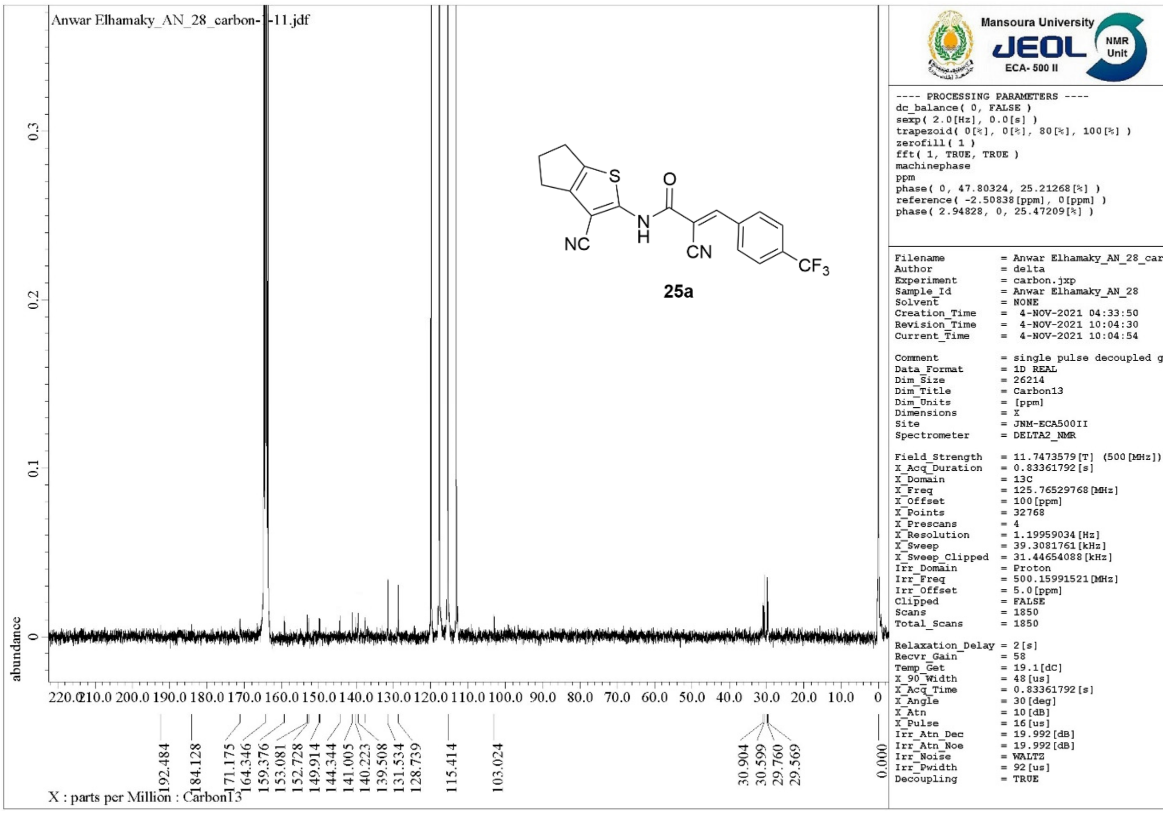

Figure S4. ^{13}C NMR (125 MHz, CF_3COOD) spectrum of compound **25a**.

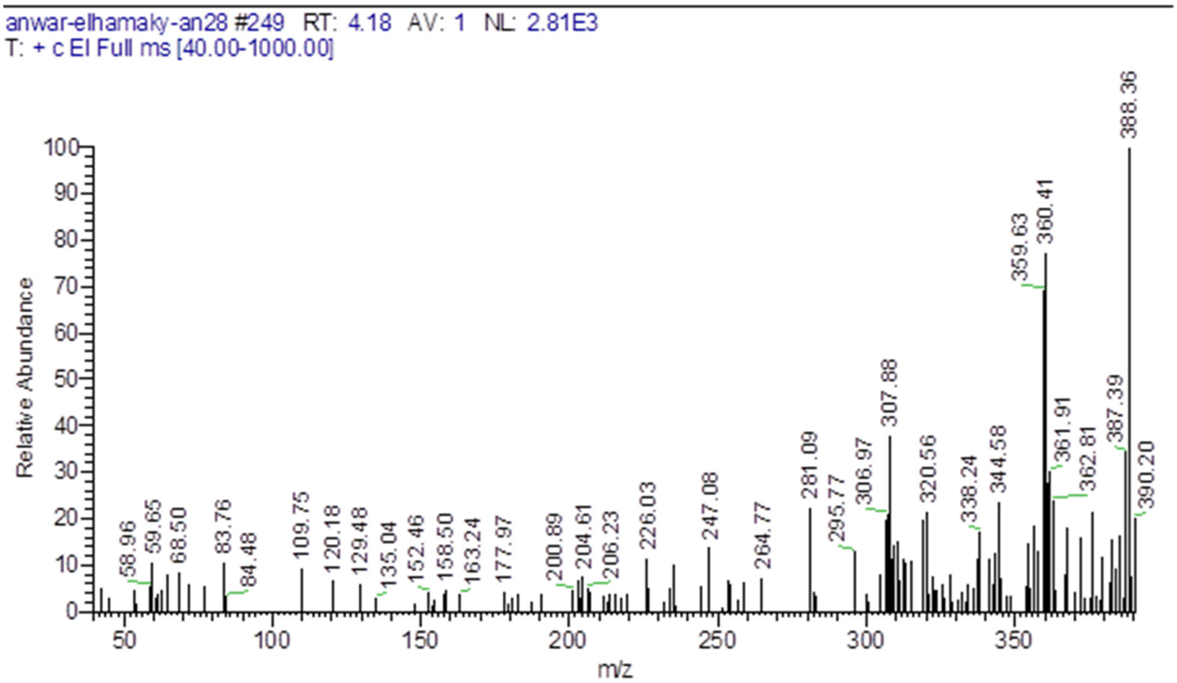

Figure S5. Mass spectrum of compound **25a**.

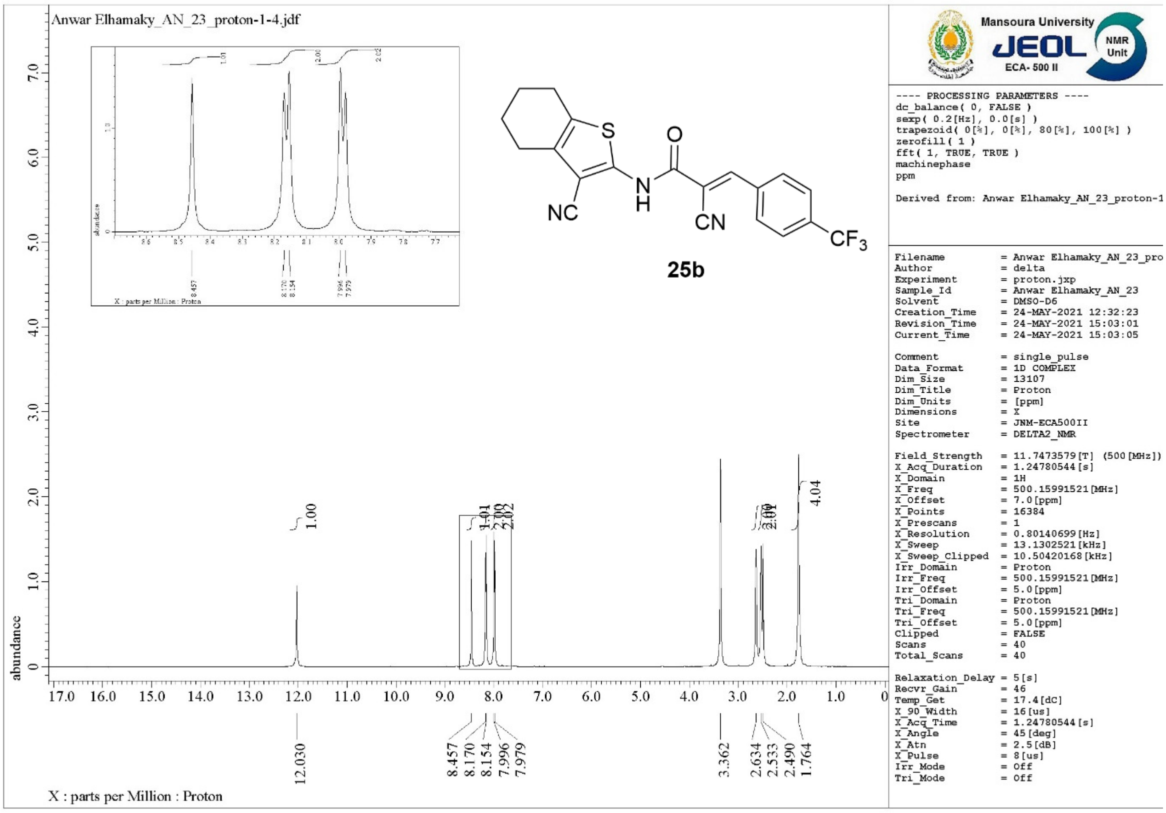

Figure S6. ^{1}H NMR (500 MHz, DMSO-d_6) spectrum of compound **25b** in *E* configuration.

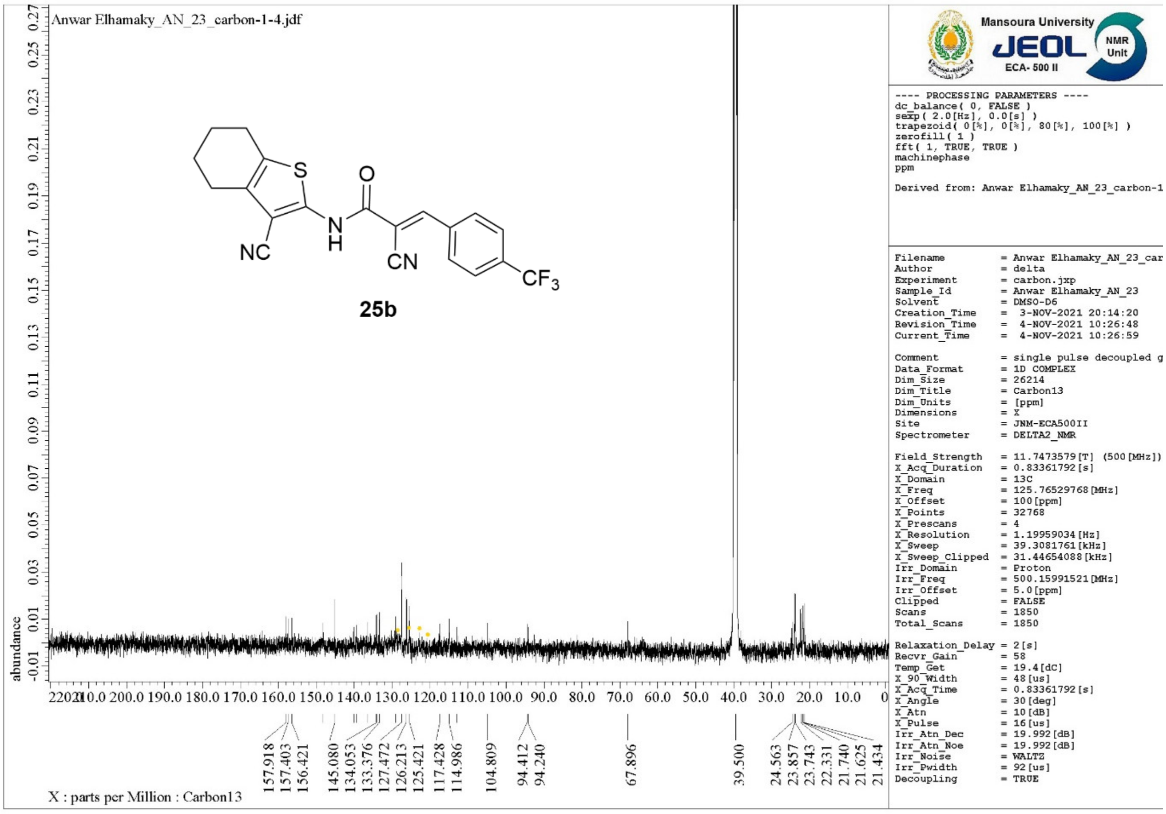

Figure S7. ^{13}C NMR (125 MHz, DMSO-d_6) spectrum of compound **25b**.

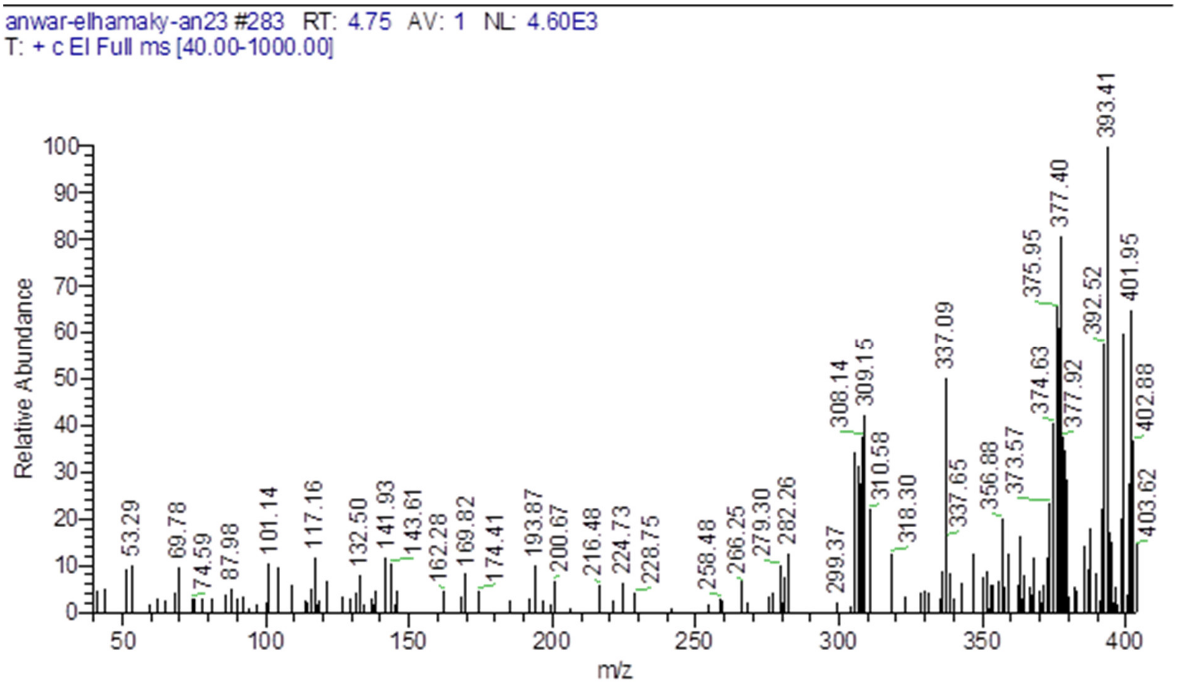

Figure S8. Mass spectrum of compound **25b**.

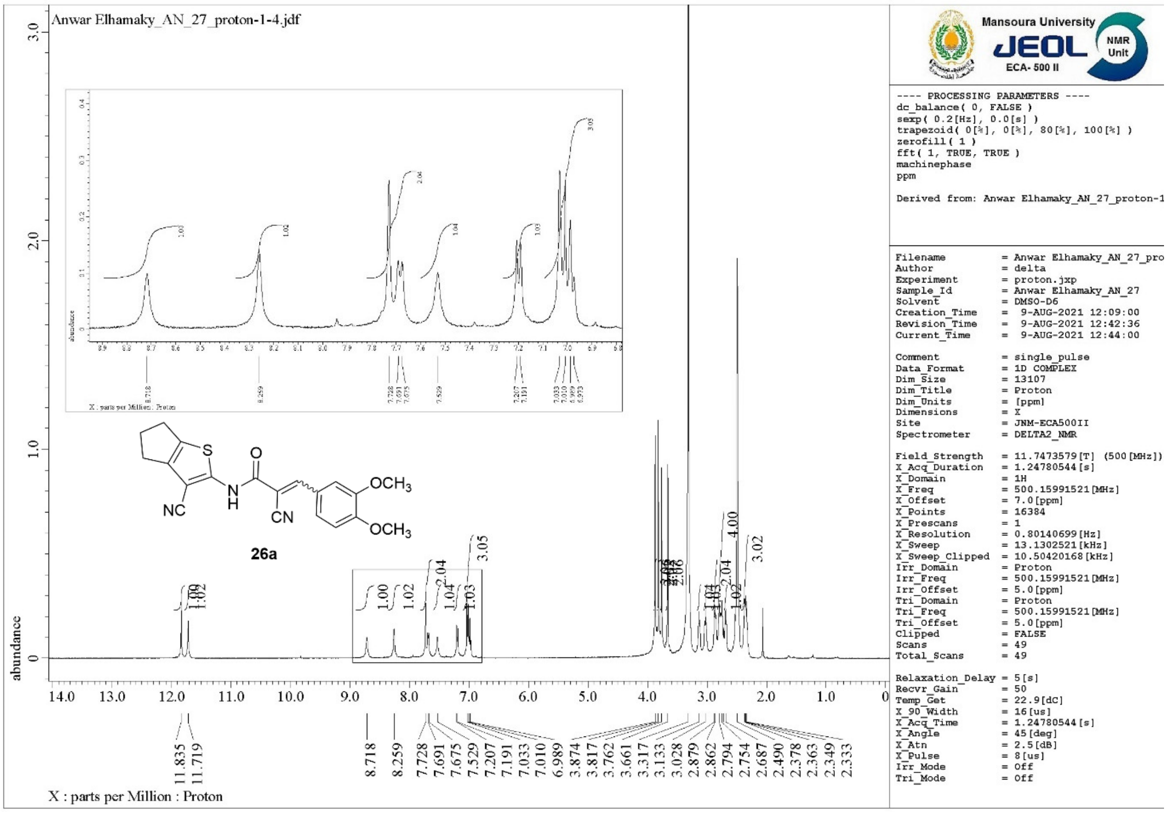

Figure S9. ^{1}H NMR (500 MHz, DMSO-d_6) spectrum of compound **26a** as *E*/*Z* mixture.

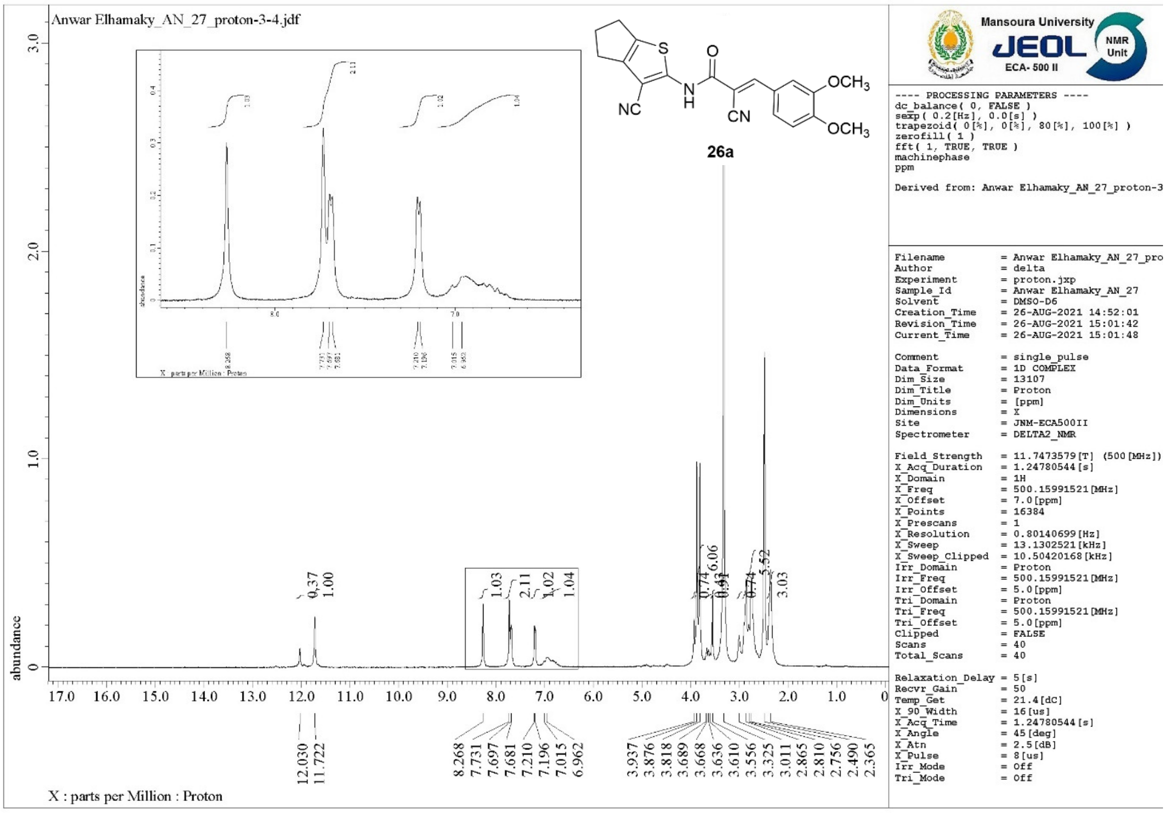

Figure S10. ^{1}H NMR (500 MHz, DMSO-d_6) spectrum of compound **26a** as a pure *E* configuration.

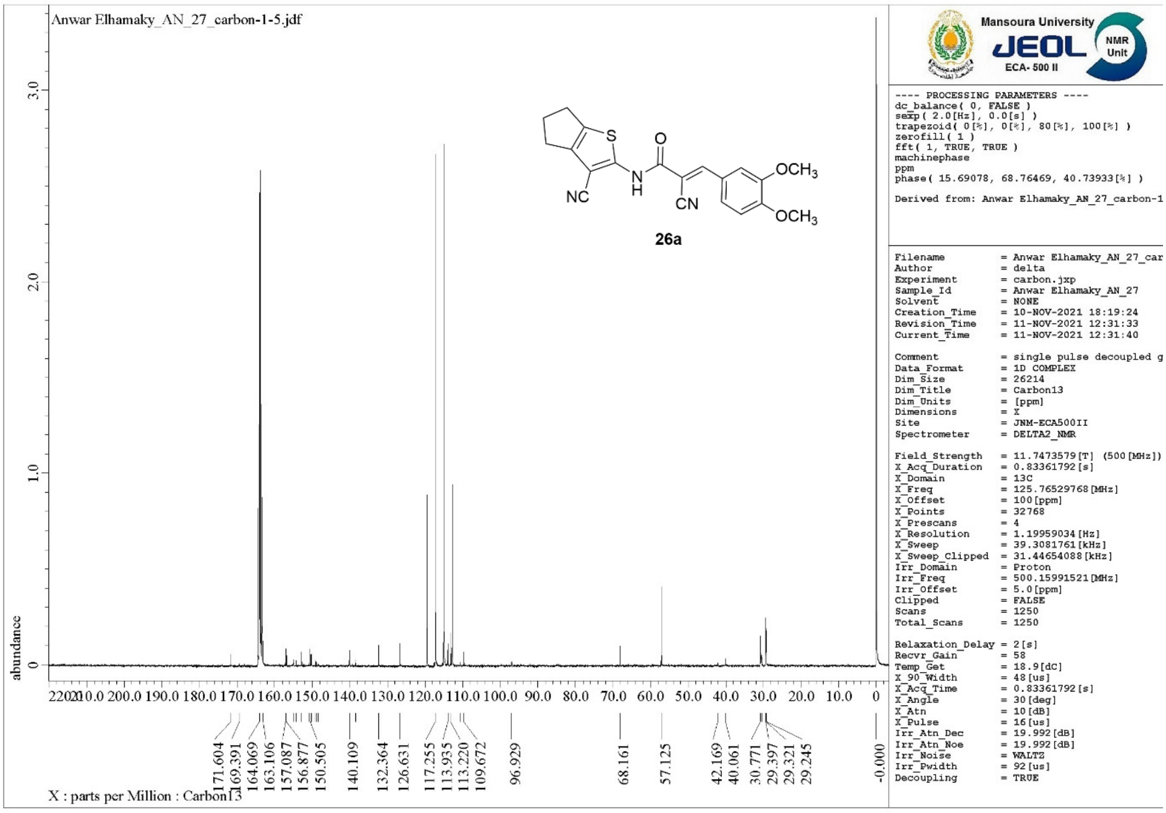

Figure S11 a. ^{13}C NMR (125 MHz, CF_3COOD) spectrum of compound **26a**.

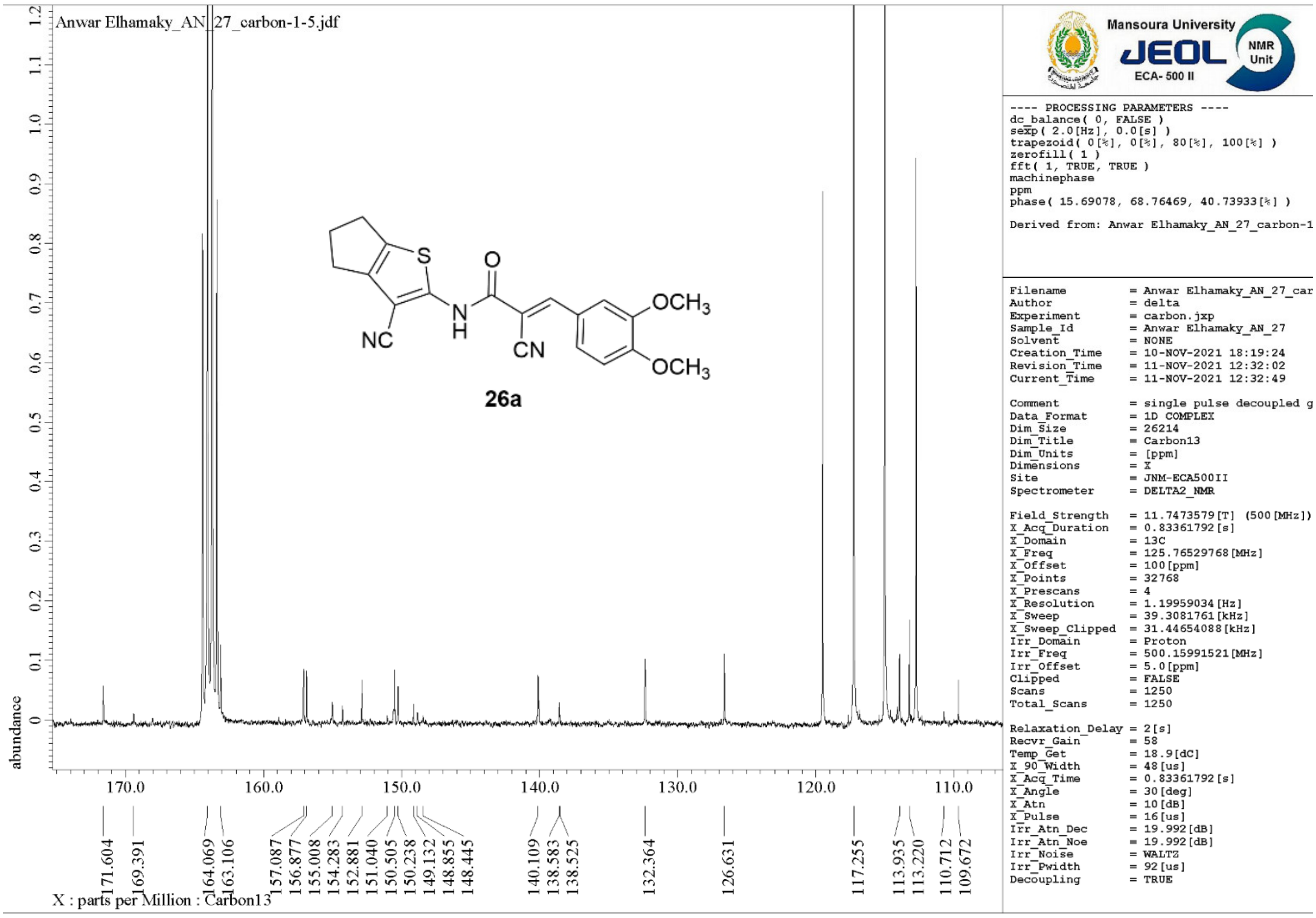

Figure S11 b. ^{13}C NMR (125 MHz, CF_3COOD) spectrum of compound **26a** (focusing on 109.0-172.0 ppm).

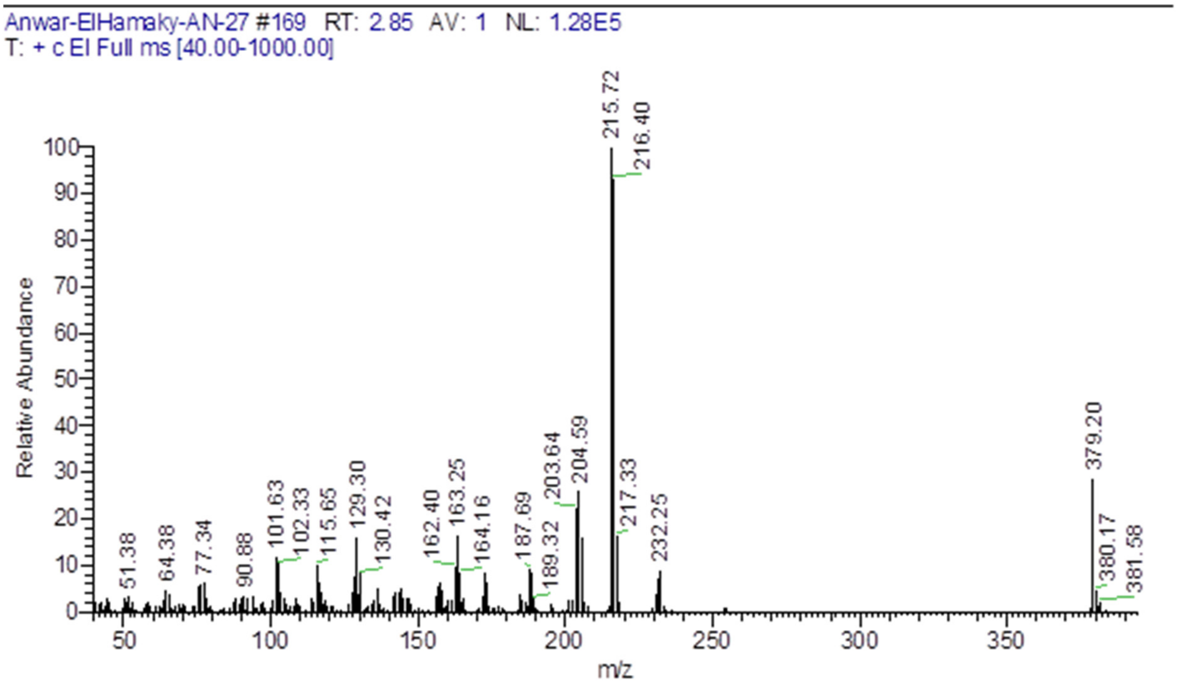

Figure S12. Mass spectrum of compound **26a**.

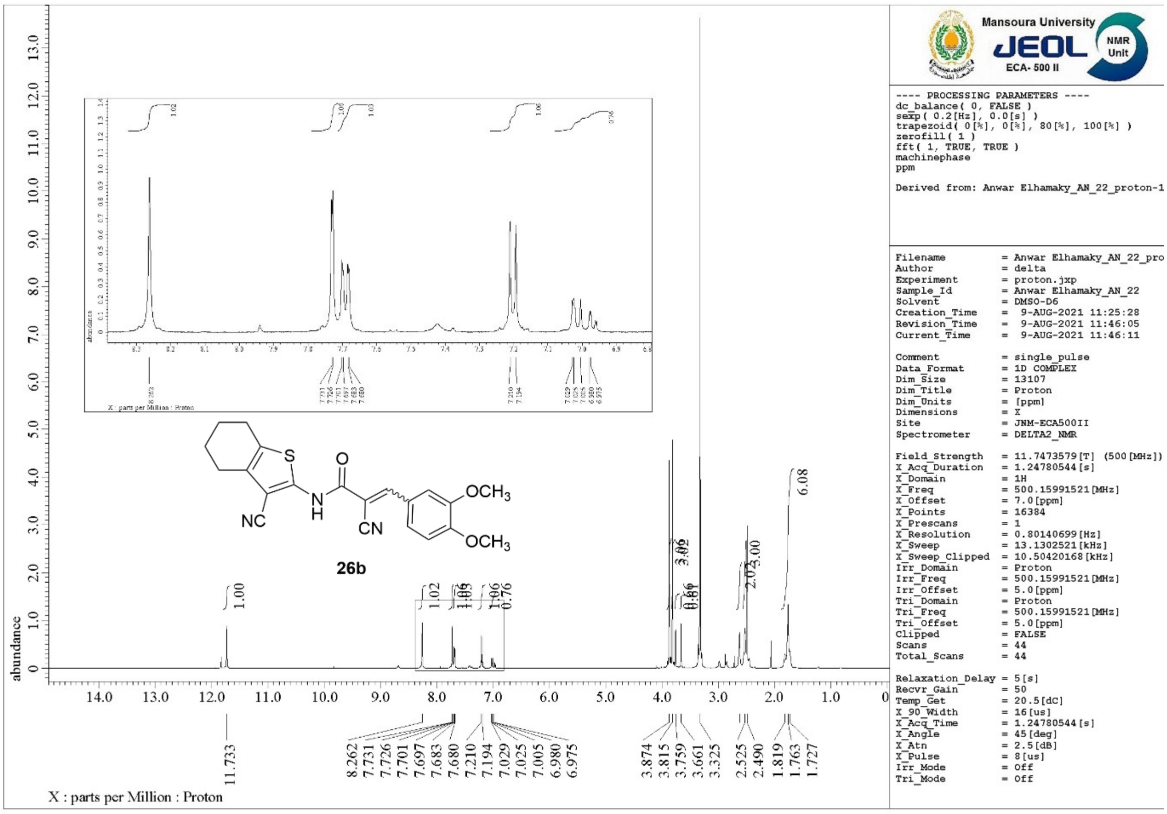

Figure S13. ^{1}H NMR (500 MHz, DMSO-d_6) spectrum of compound **26b** as *E*/*Z* mixture.

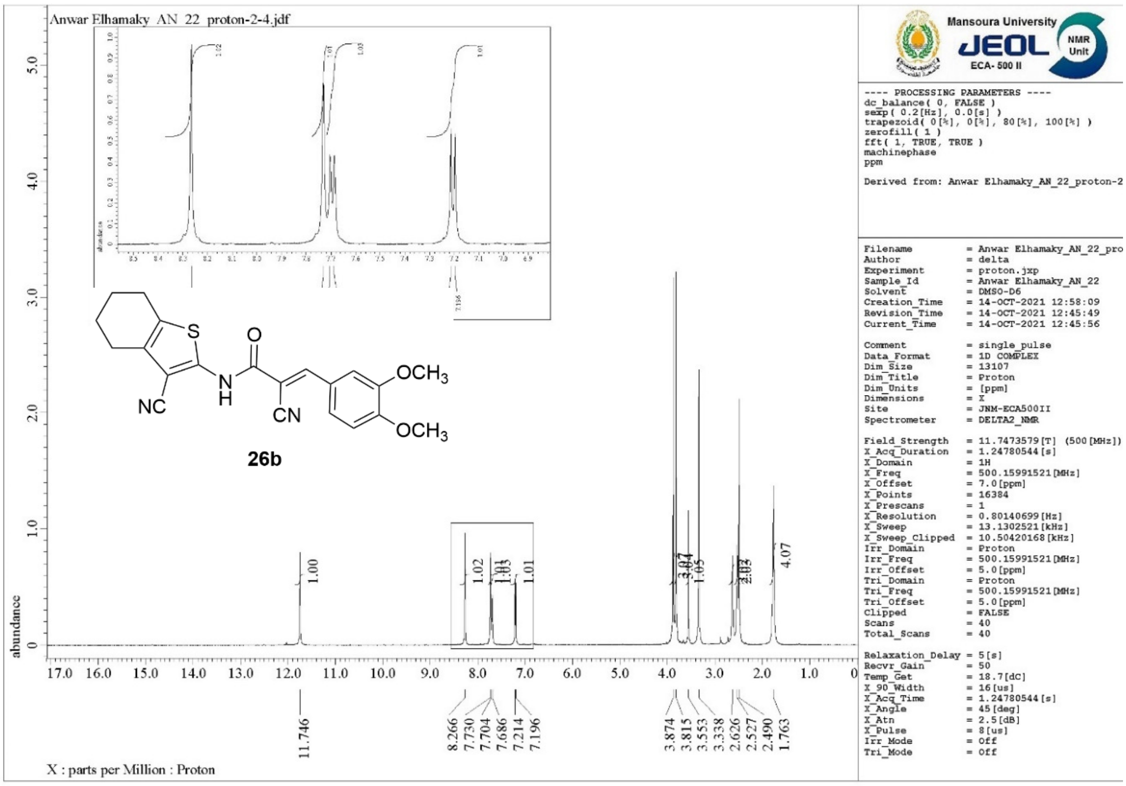

Figure S14. ^{1}H NMR (500 MHz, DMSO-d_6) spectrum of compound **26b** as a pure *E* configuration.

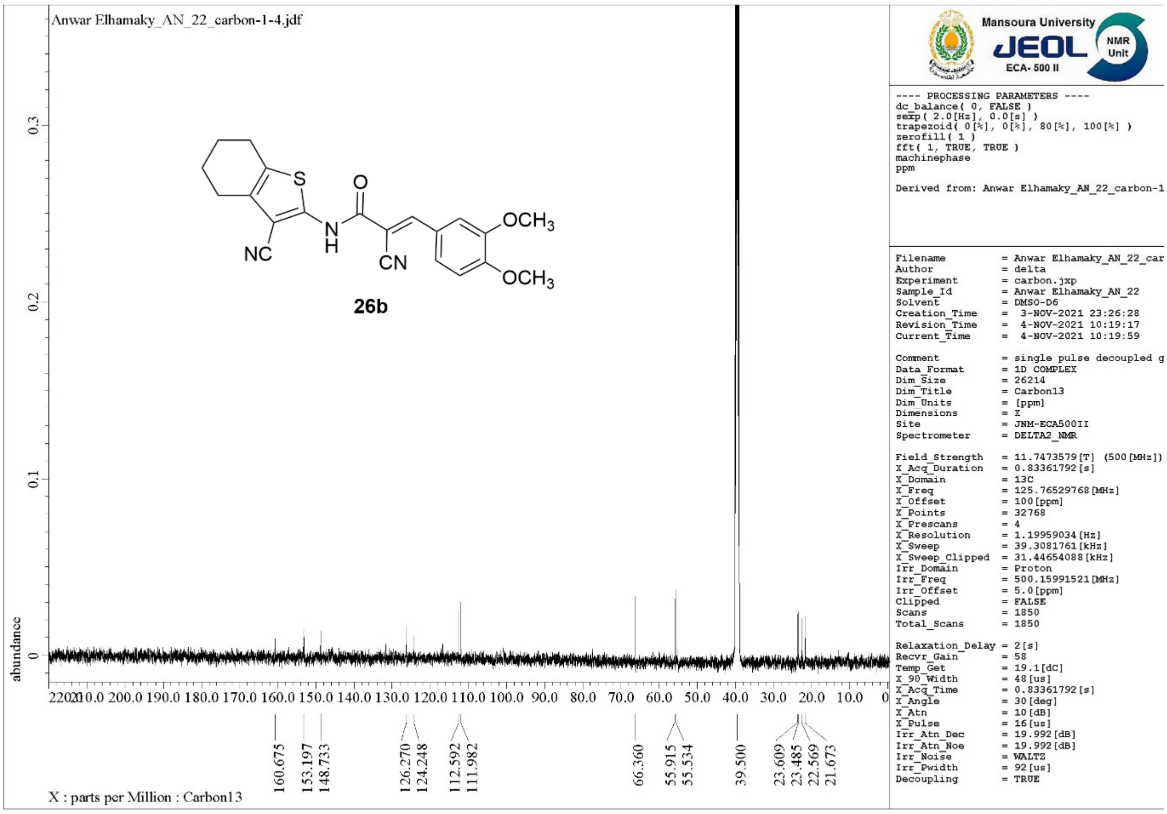

Figure S15. ^{13}C NMR (125 MHz, DMSO-d_6) spectrum of compound **26b**.

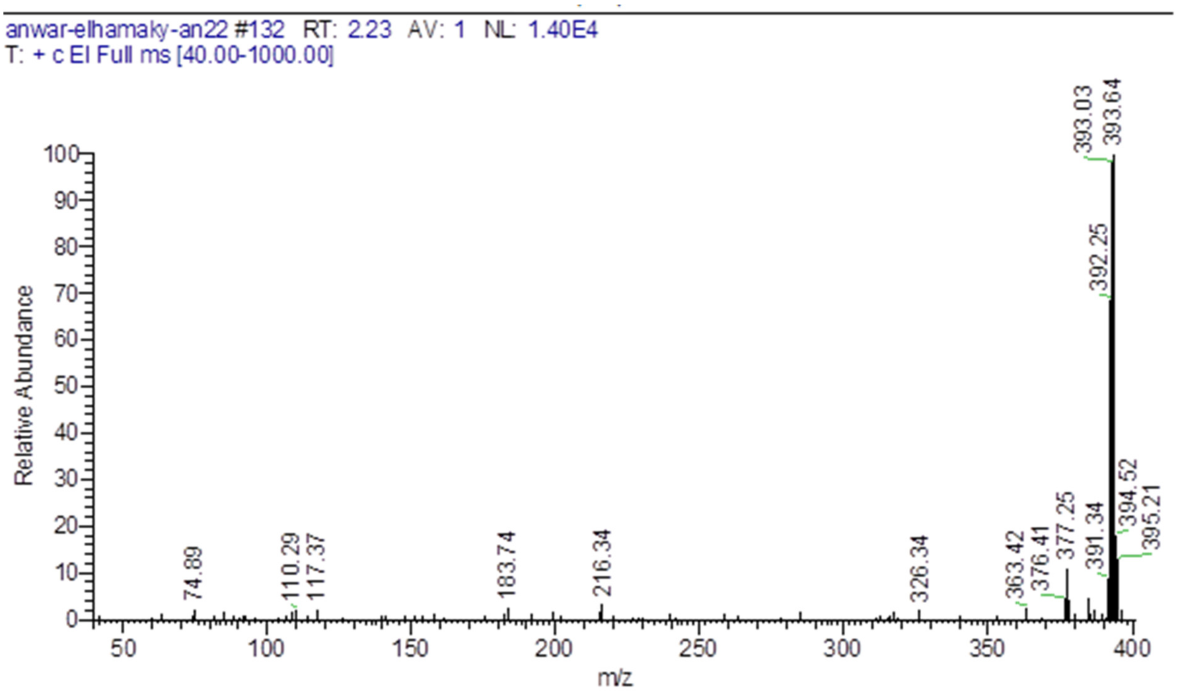

Figure S16. Mass spectrum of compound **26b**.

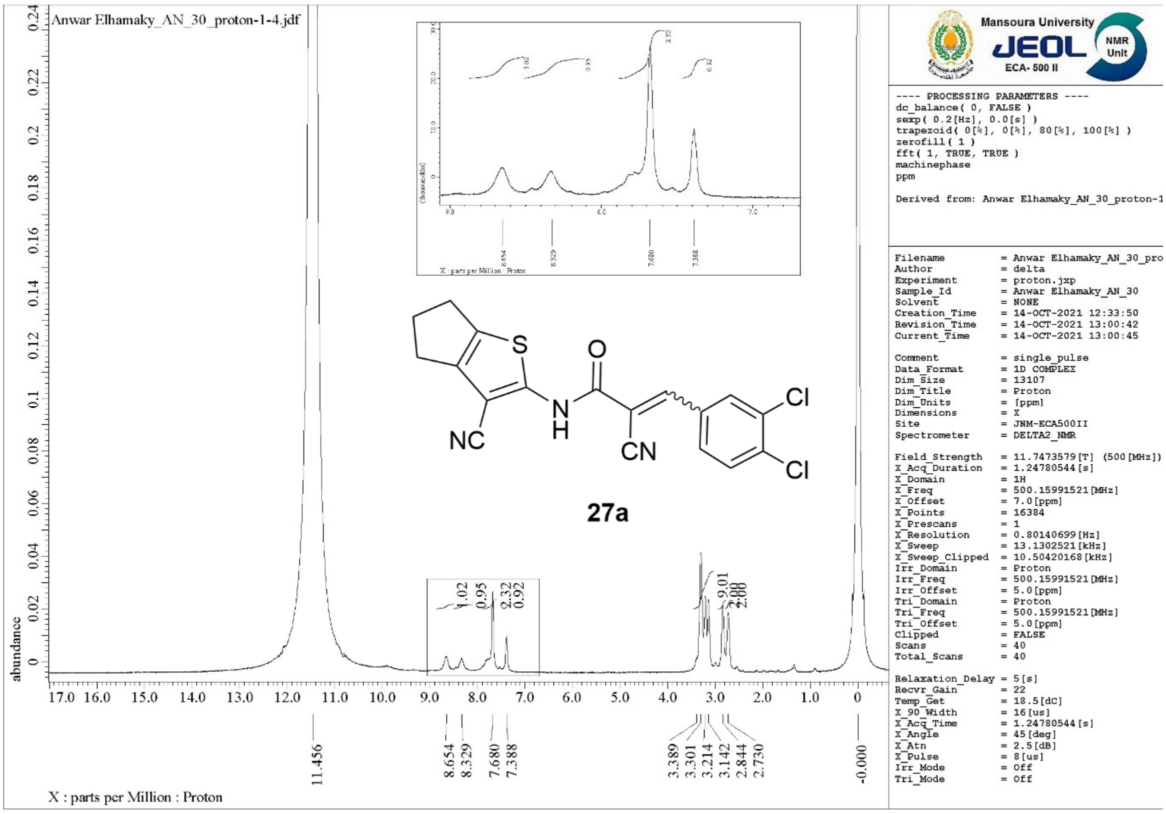

Figure S17. ^{1}H NMR (500 MHz, CF_3COOD) spectrum of compound **27a** as *E*/*Z* mixture.

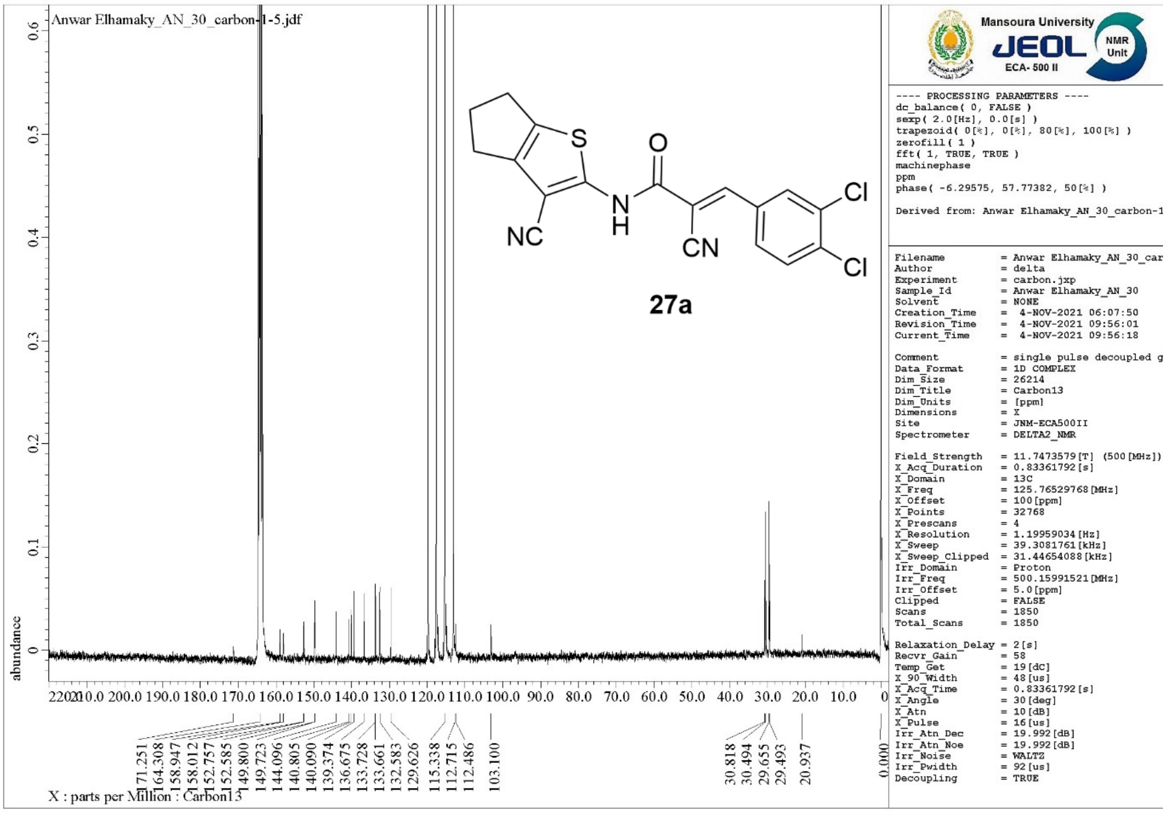

Figure S18. ^{13}C NMR (125 MHz, CF_3COOD) spectrum of compound **27a**.

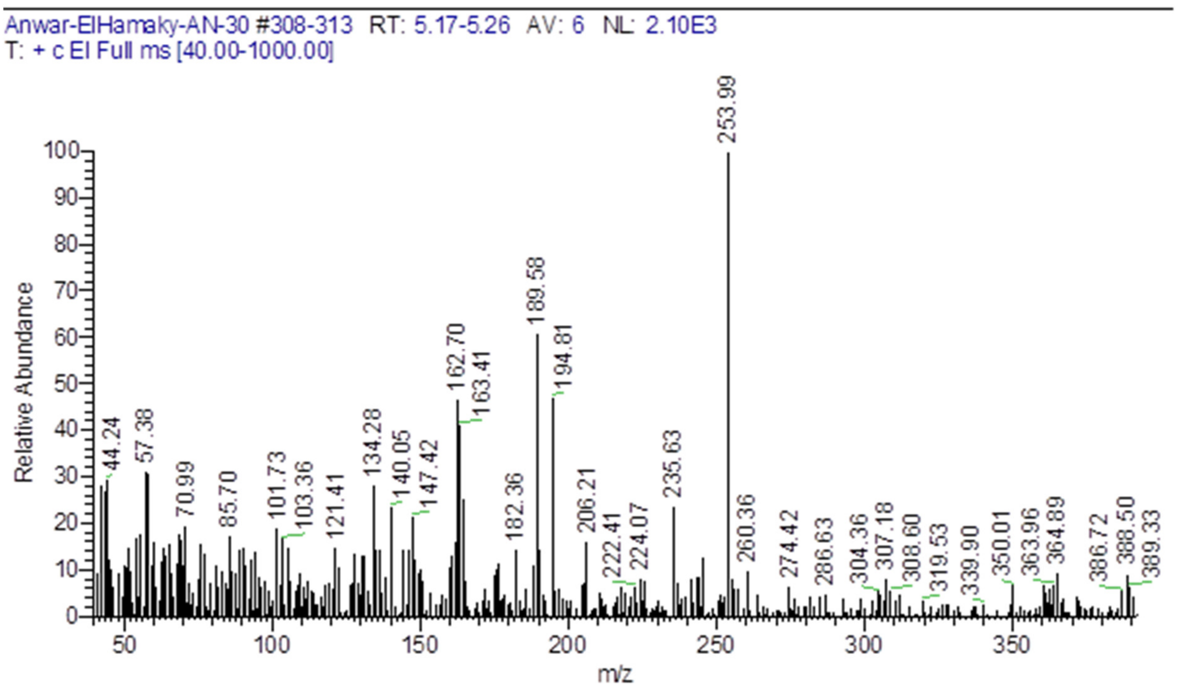

Figure S19. Mass spectrum of compound **27a**.

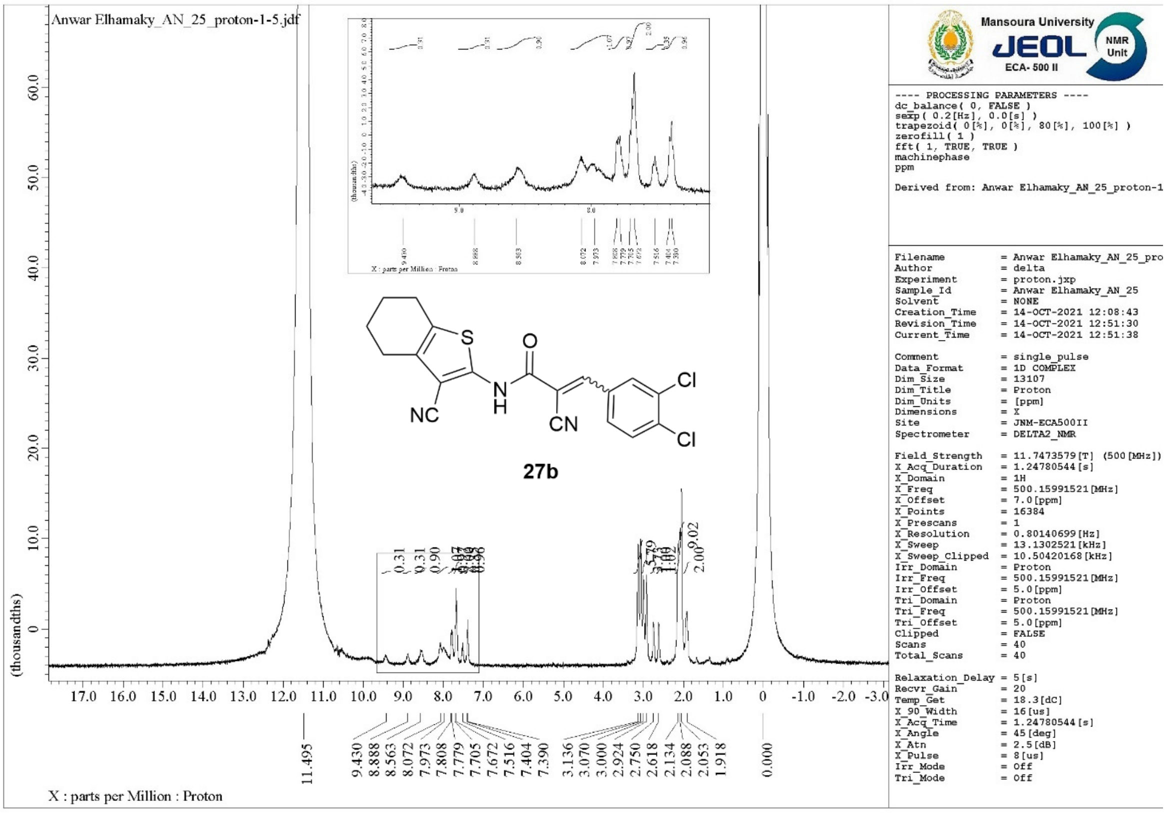

Figure S20. ^{1}H NMR (500 MHz, CF_3COOD) spectrum of compound **27b** as *E*/*Z* mixture.

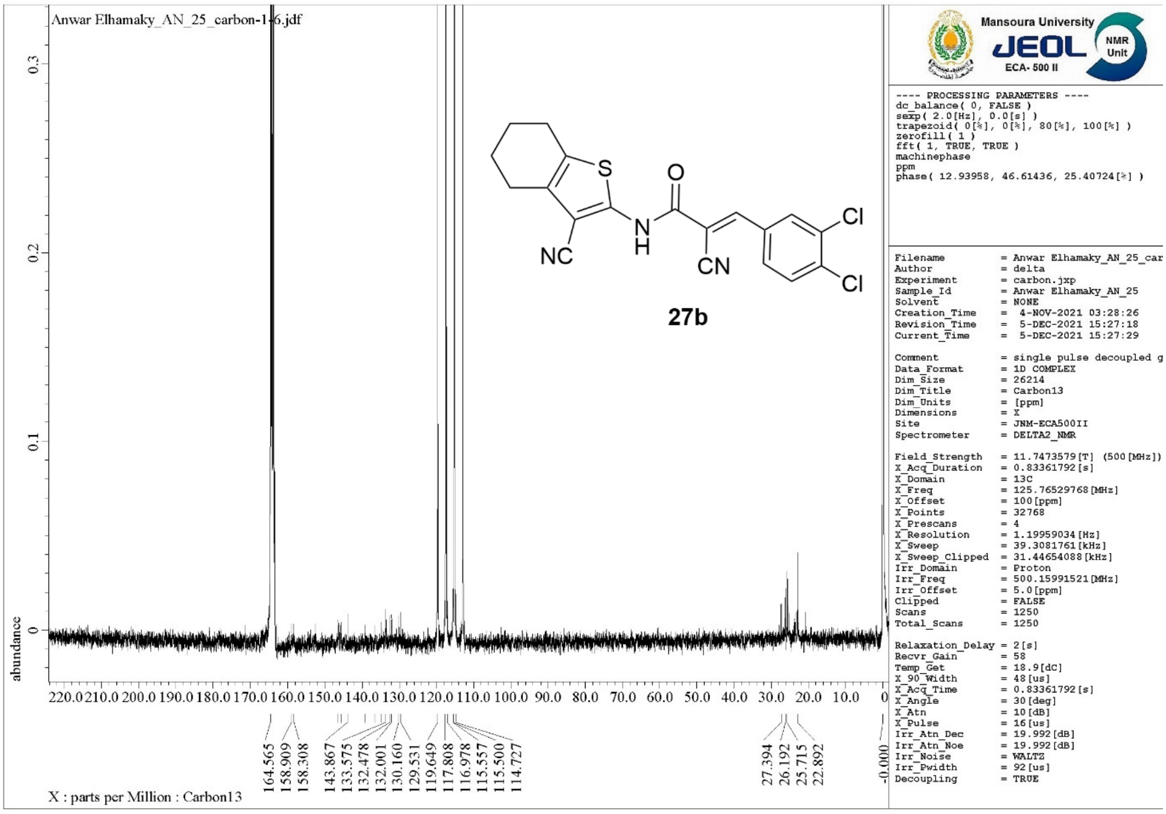

Figure S21 a. ^{13}C NMR (125 MHz, CF_3COOD) spectrum of compound **27b**.

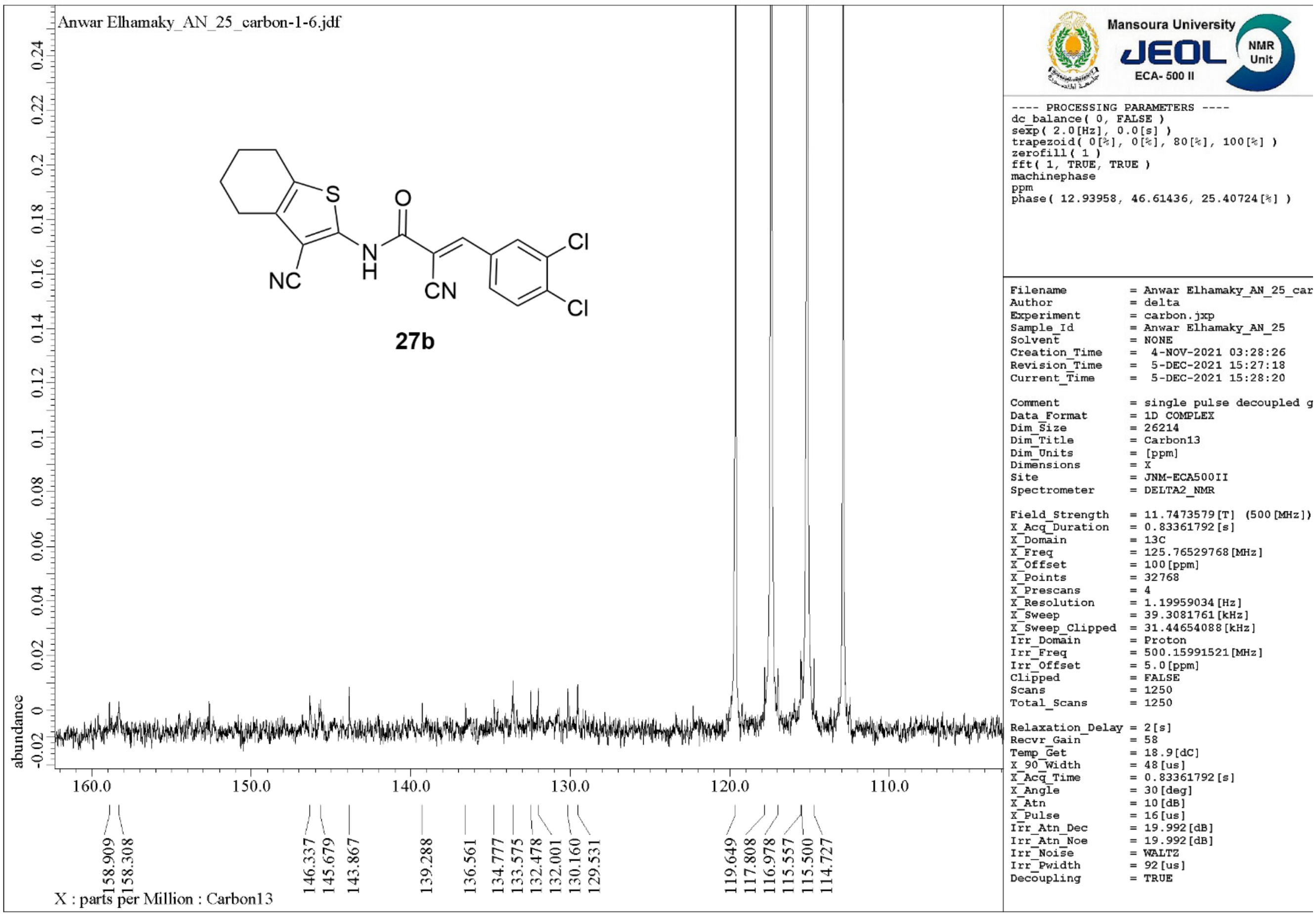

Figure S21 b. ^{13}C NMR (125 MHz, CF_3COOD) spectrum of compound **27b** (focusing on 110.0-160.0 ppm).

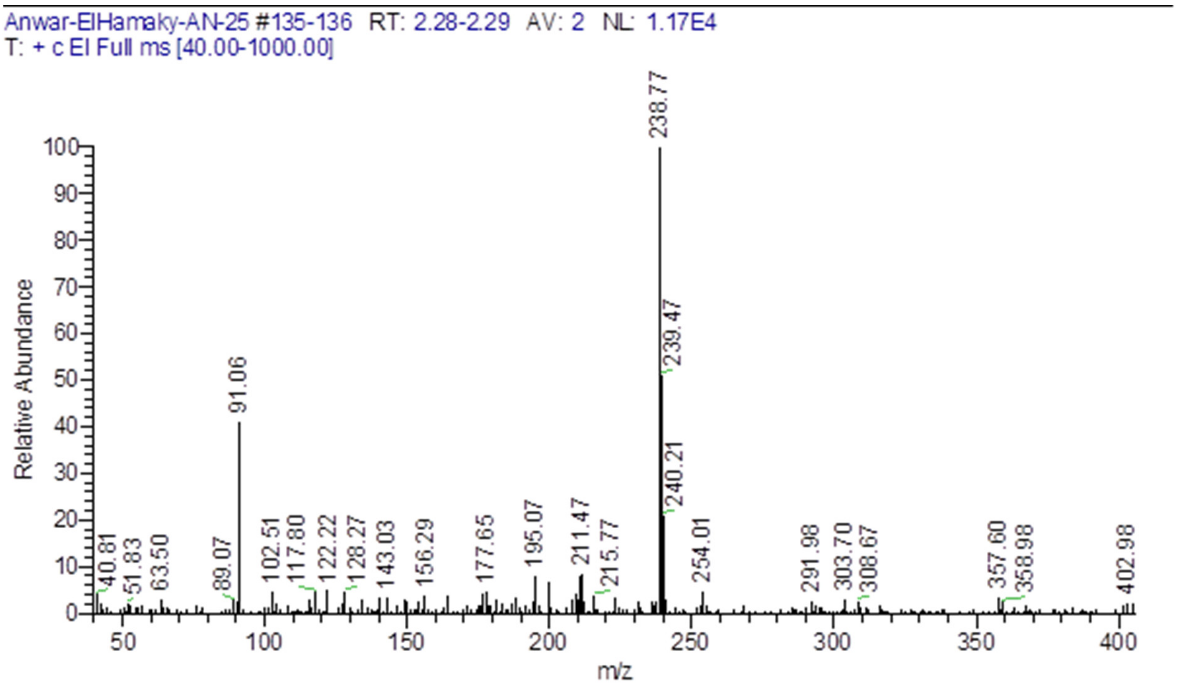

Figure S22. Mass spectrum of compound **27b**.

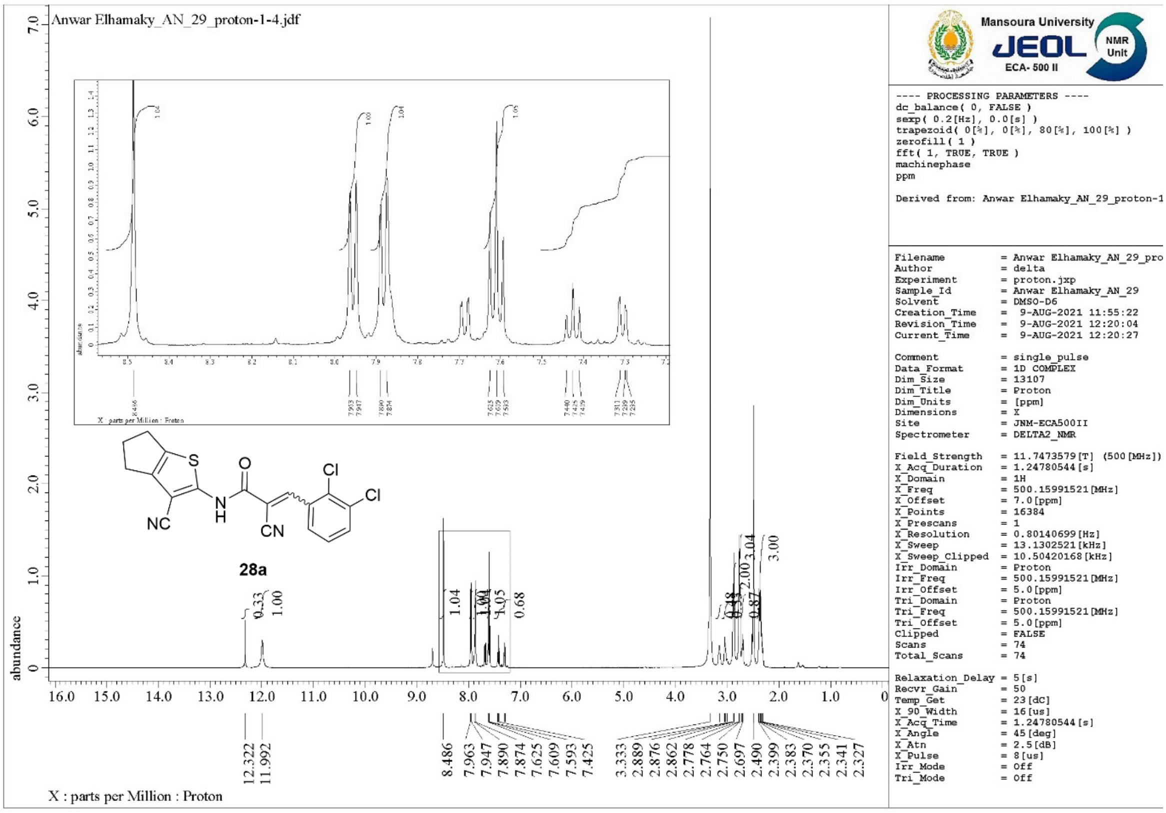

Figure S23. ^{1}H NMR (500 MHz, DMSO-d_6) spectrum of compound **28a** as *E*/*Z* mixture.

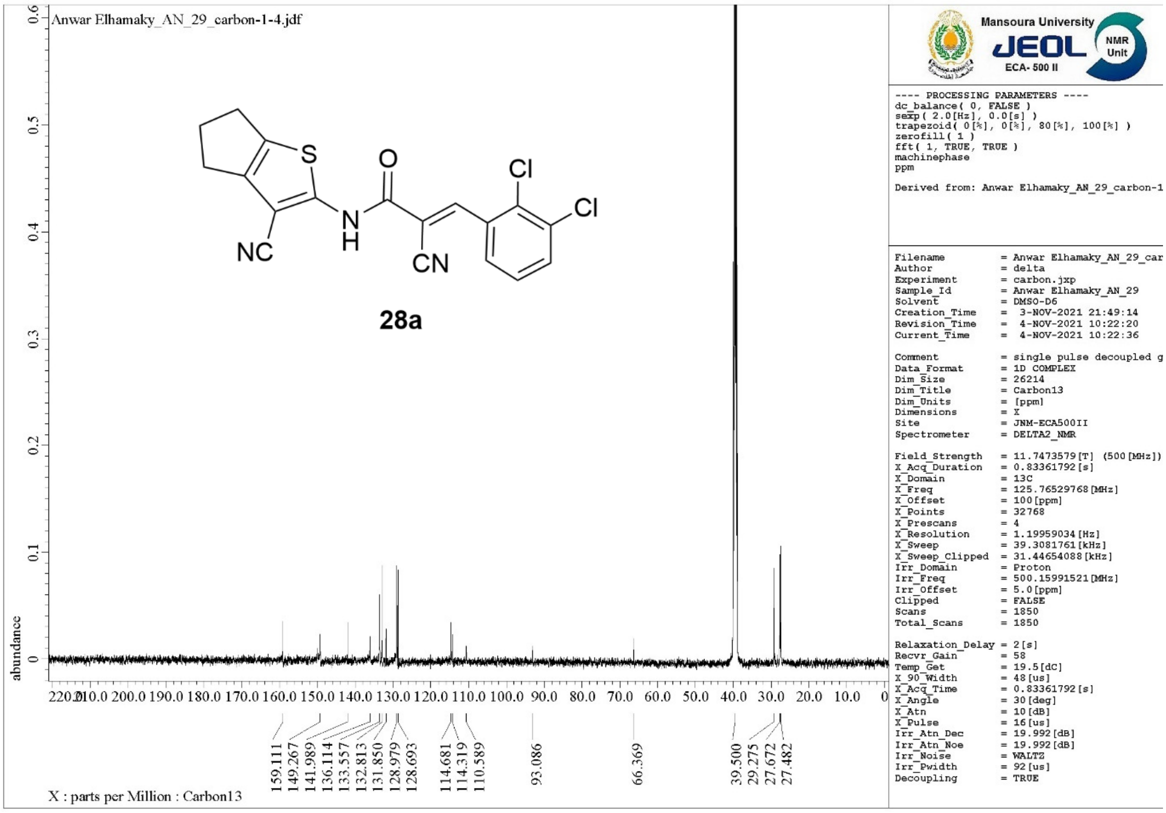

Figure S24. ^{13}C NMR (125 MHz, DMSO-d_6) spectrum of compound **28a**.

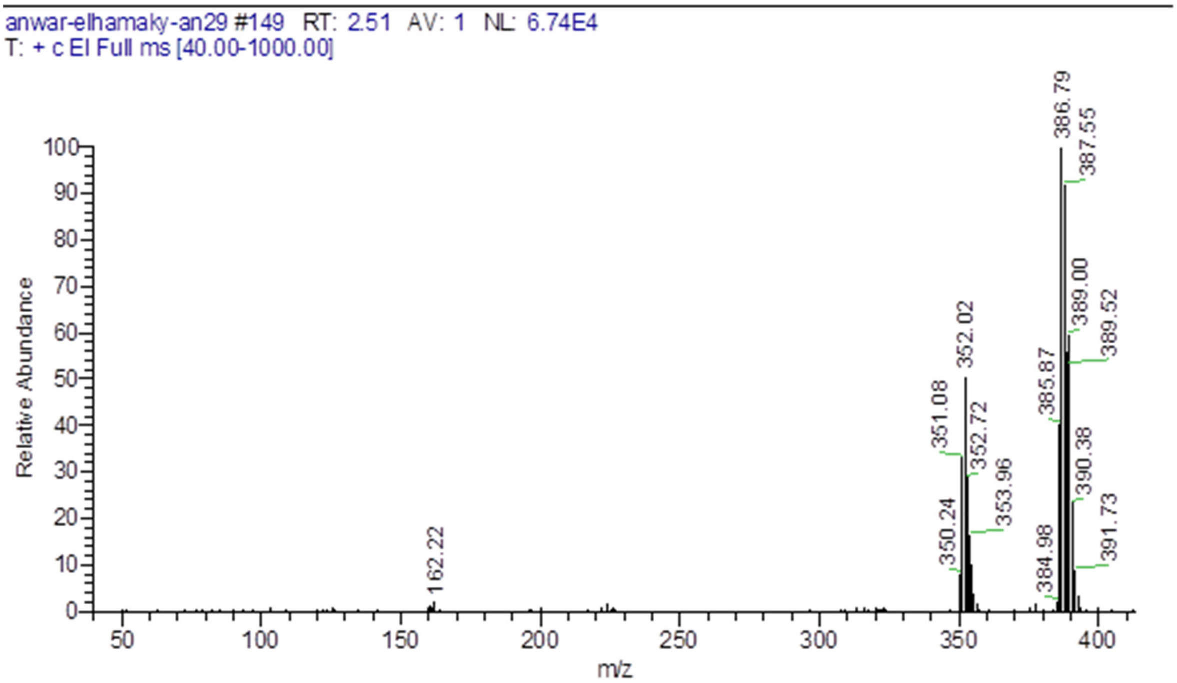

Figure S25. Mass spectrum of compound **28a**.

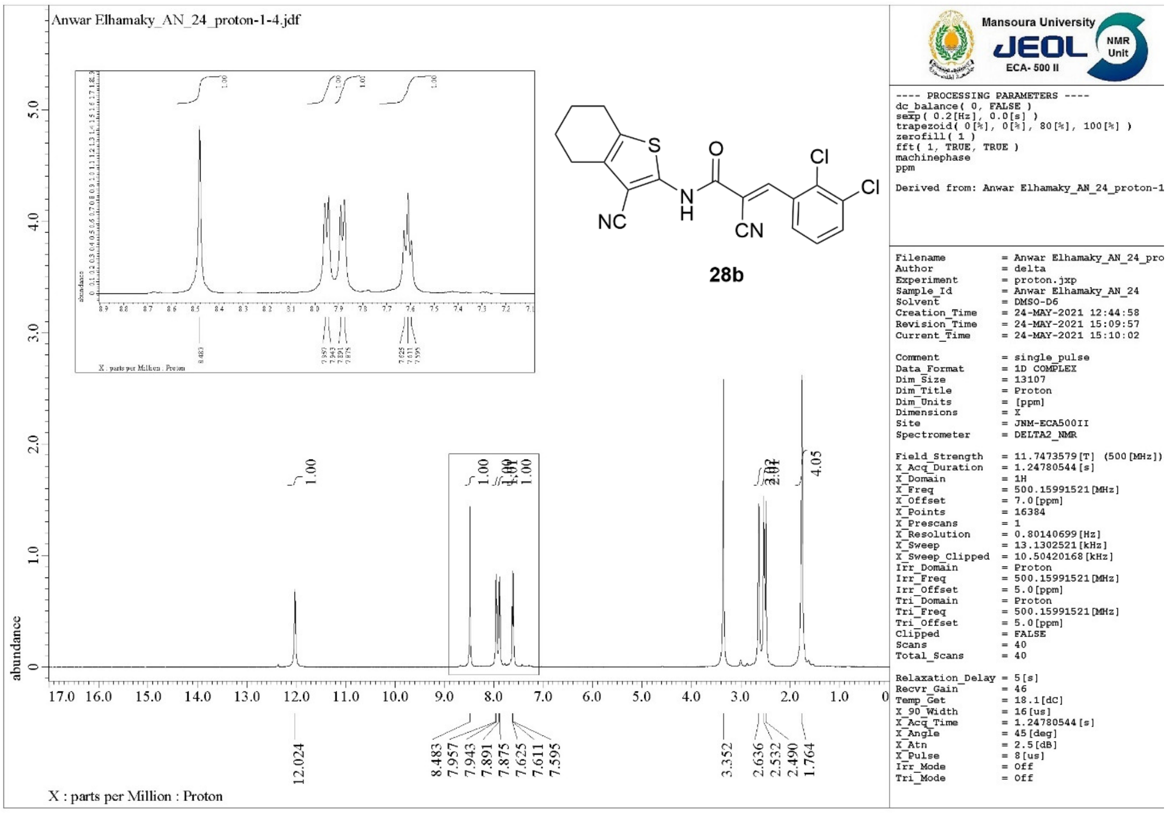

Figure S26. ^{1}H NMR (500 MHz, DMSO-d_6) spectrum of compound **28b** in *E* configuration.

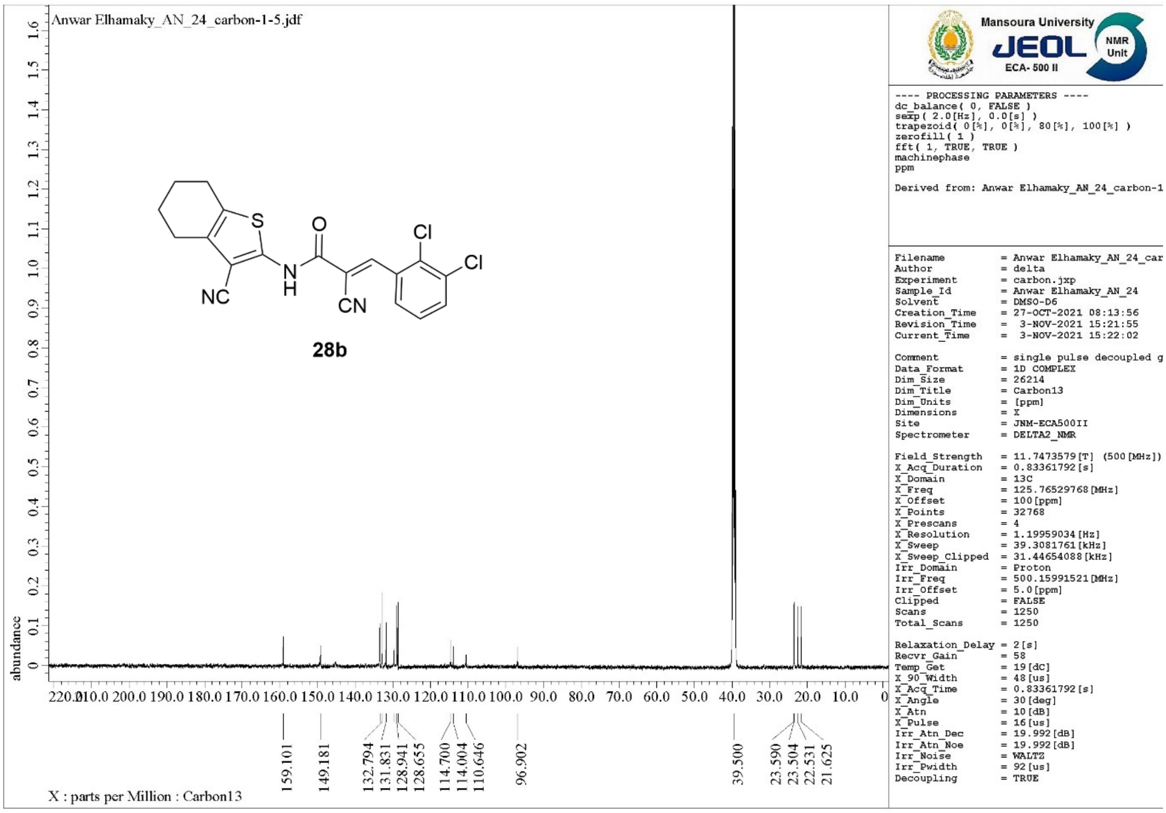

Figure S27. ^{13}C NMR (125 MHz, DMSO-d_6) spectrum of compound **28b**.

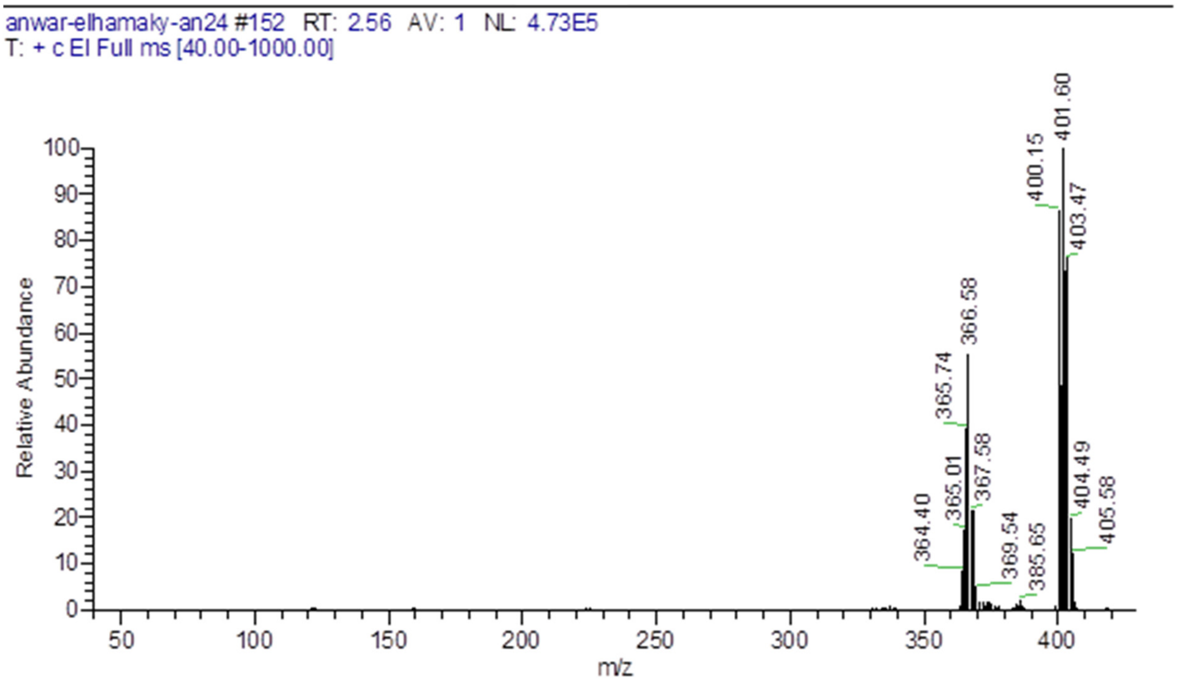

Figure S28. Mass spectrum of compound **28b**.

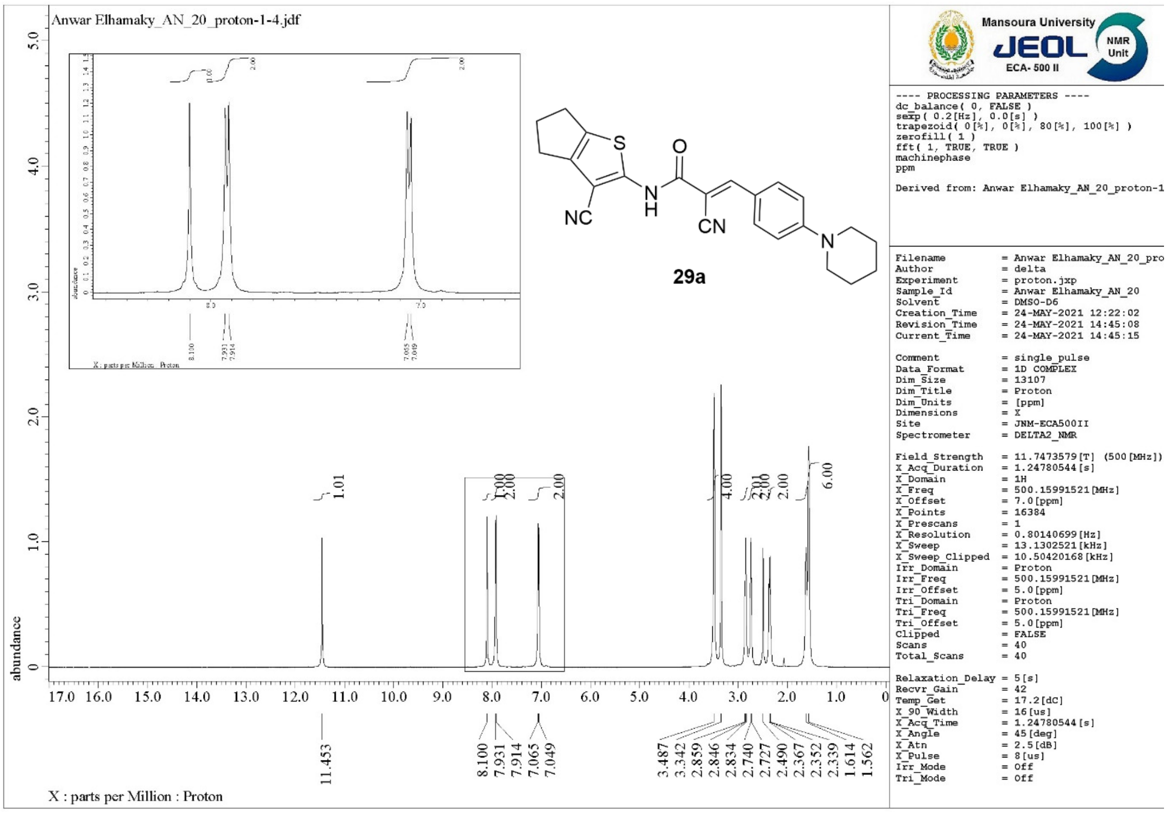

Figure S29. ^{1}H NMR (500 MHz, DMSO-d_6) spectrum of compound **29a** in *E* configuration.

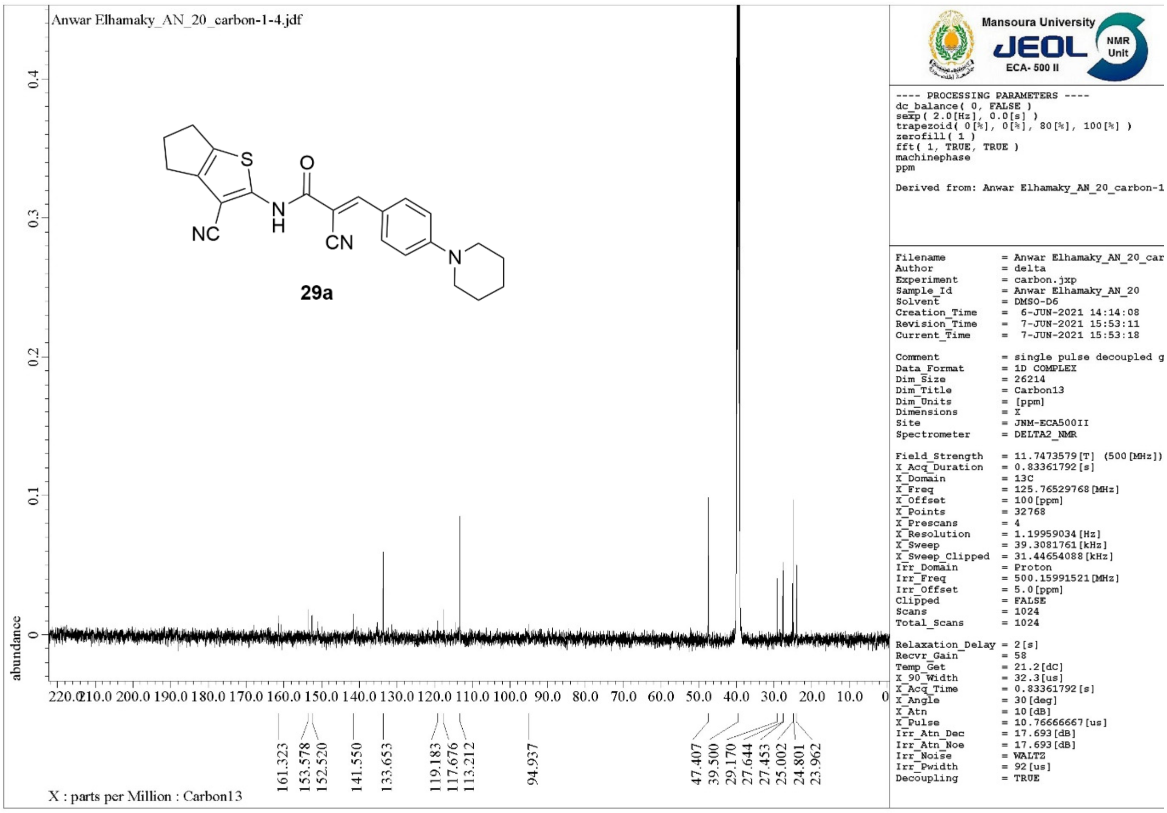

Figure S30. ^{13}C NMR (125 MHz, DMSO-d_6) spectrum of compound **29a**.

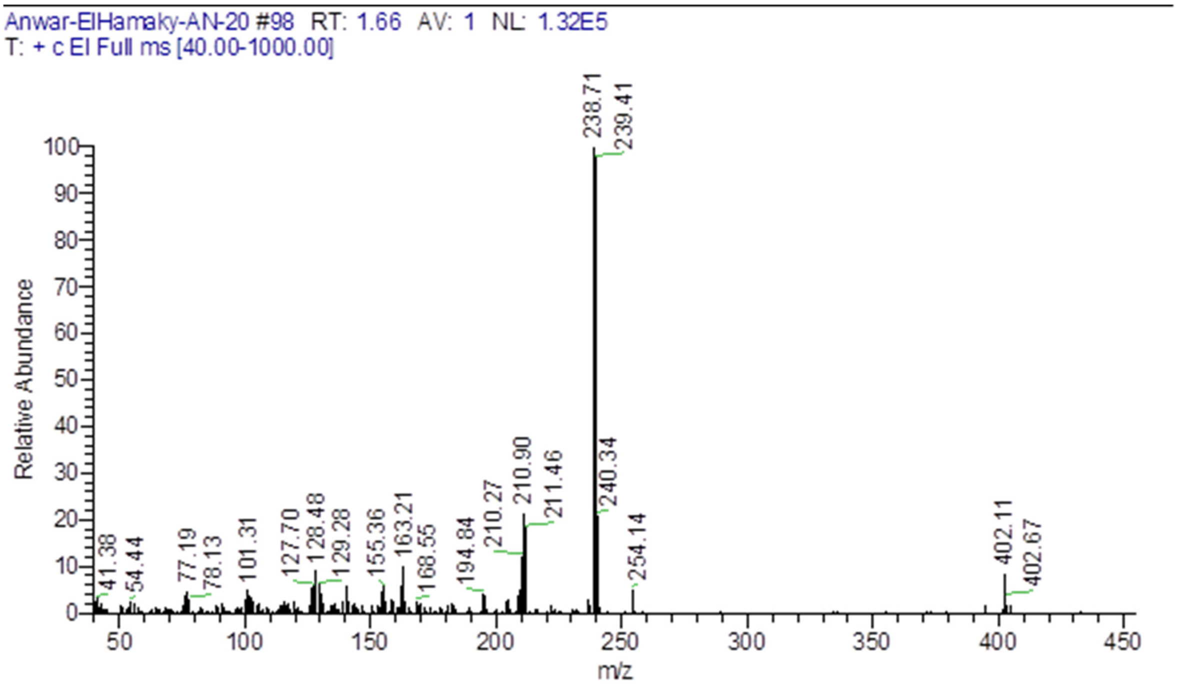

Figure S31. Mass spectrum of compound **29a**.

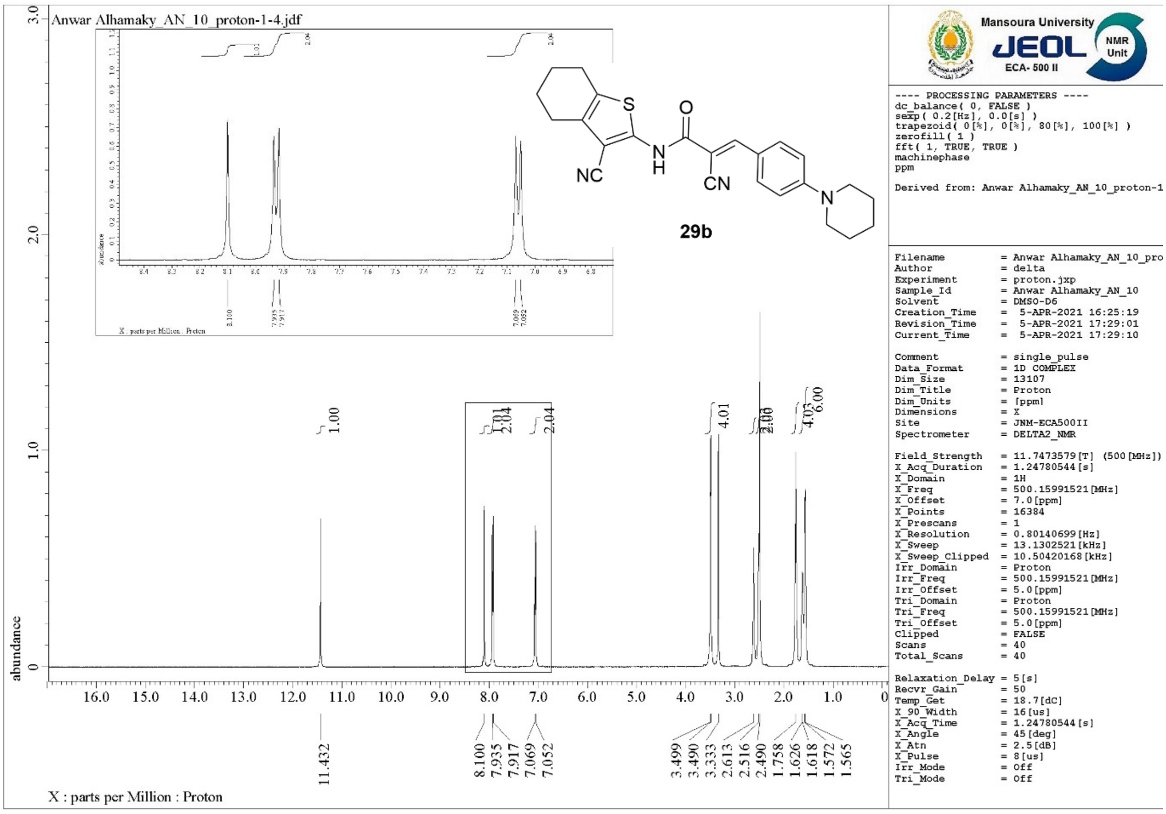

Figure S32. ^{1}H NMR (500 MHz, DMSO-d_6) spectrum of compound **29b** in *E* configuration.

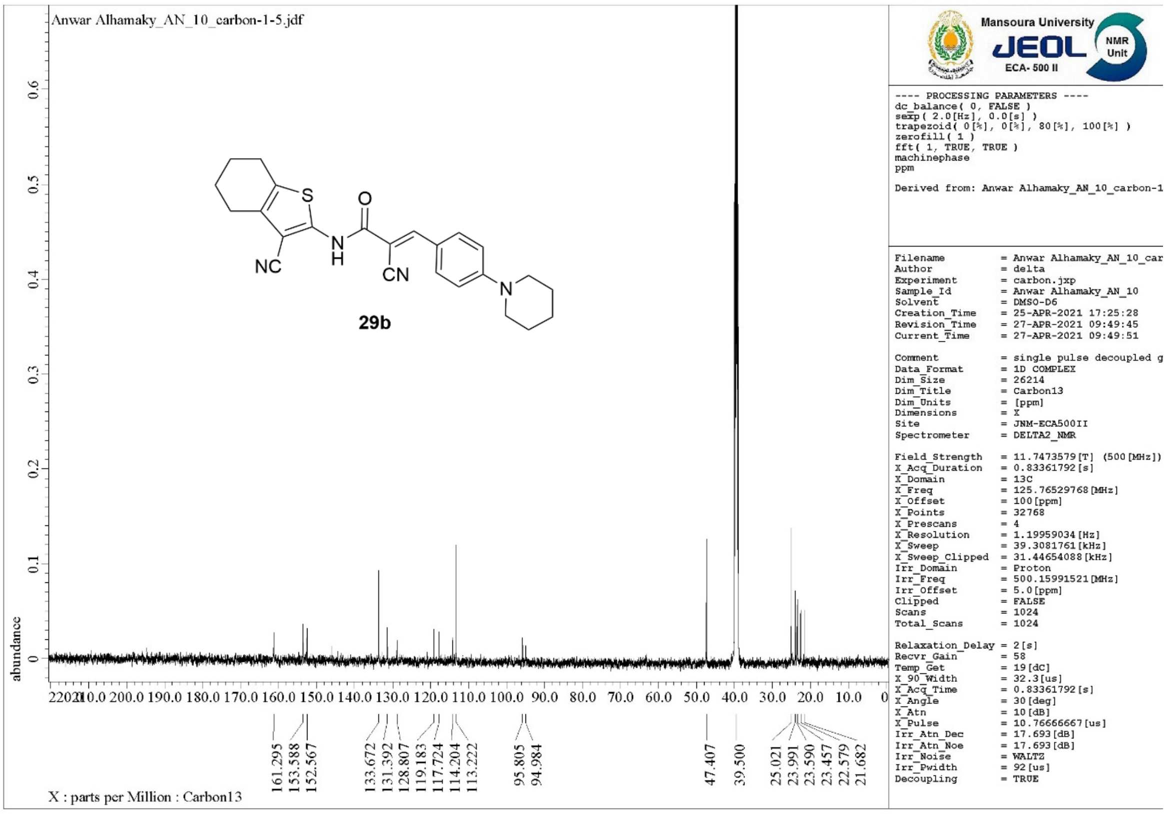

Figure S33. ^{13}C NMR (125 MHz, DMSO-d_6) spectrum of compound **29b**.

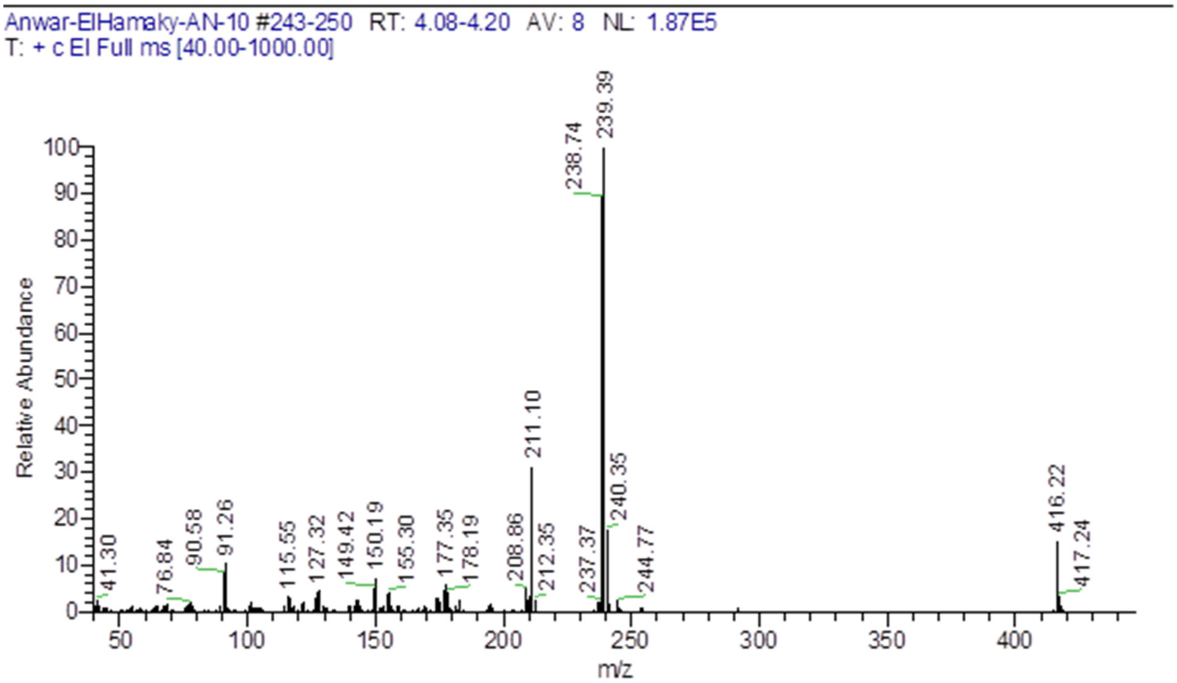

Figure S34. Mass spectrum of compound **29b**.

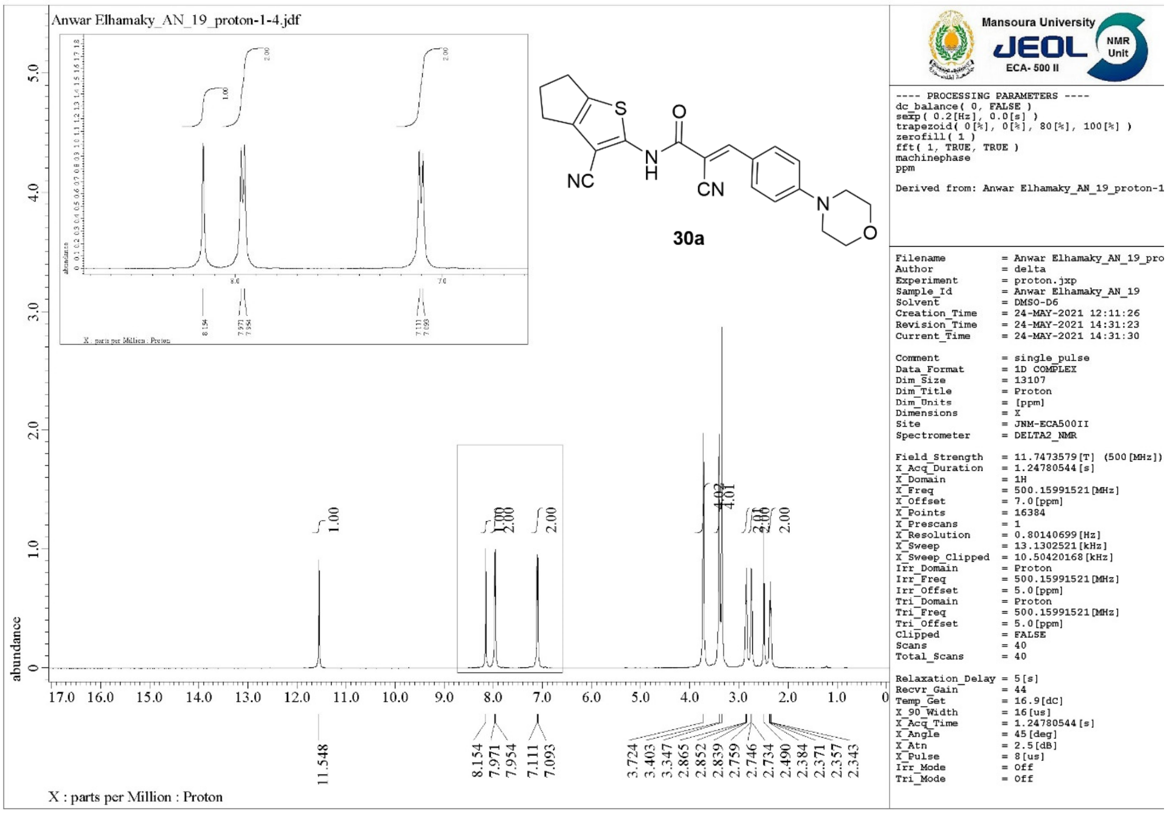

Figure S35. ^{1}H NMR (500 MHz, DMSO-d_6) spectrum of compound **30a** in *E* configuration.

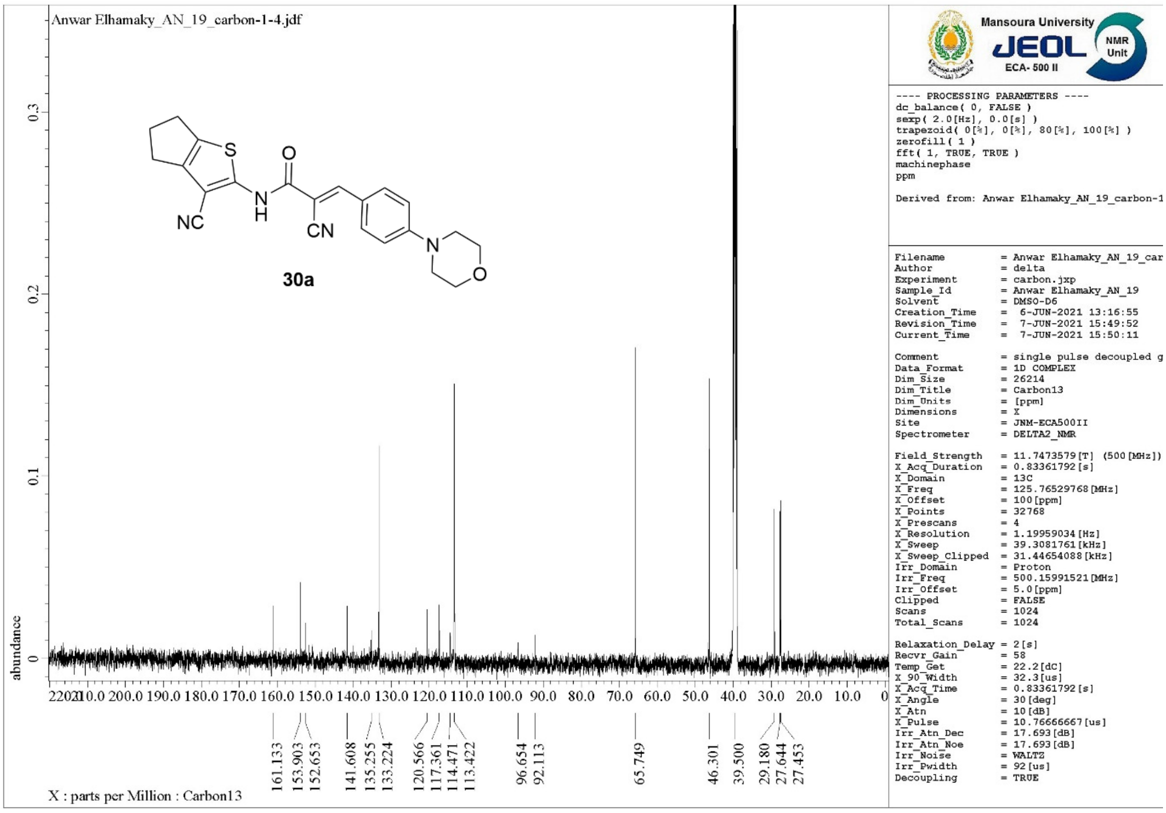

Figure S36. ^{13}C NMR (125 MHz, DMSO-d_6) spectrum of compound **30a**.

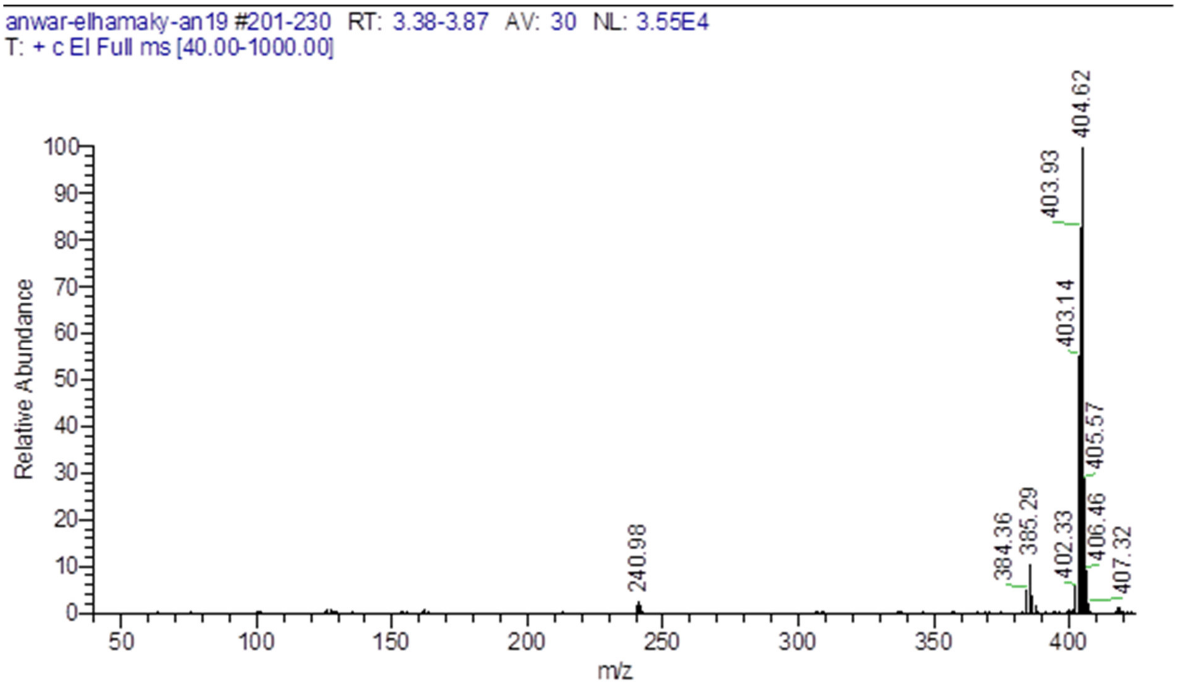

Figure S37. Mass spectrum of compound **30a**.

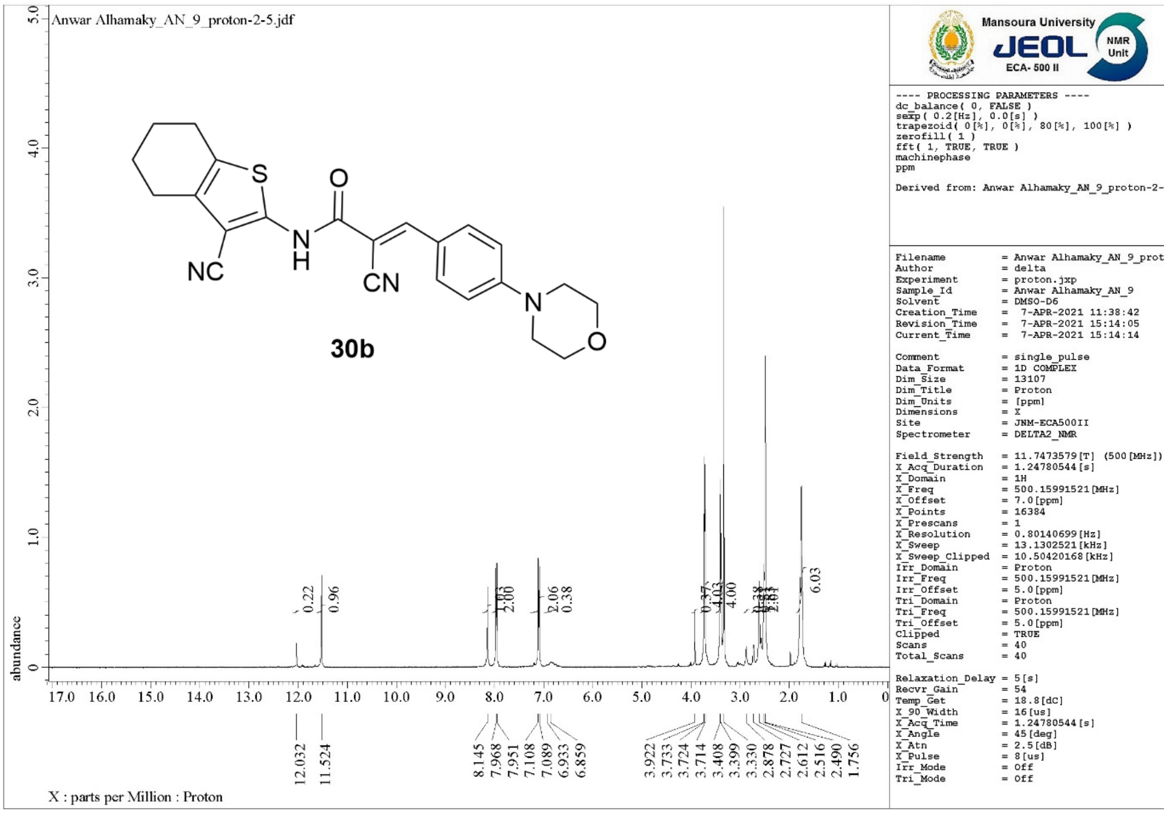

Figure S38. ^{1}H NMR (500 MHz, DMSO-d_6) spectrum of compound **30b** in *E* configuration.

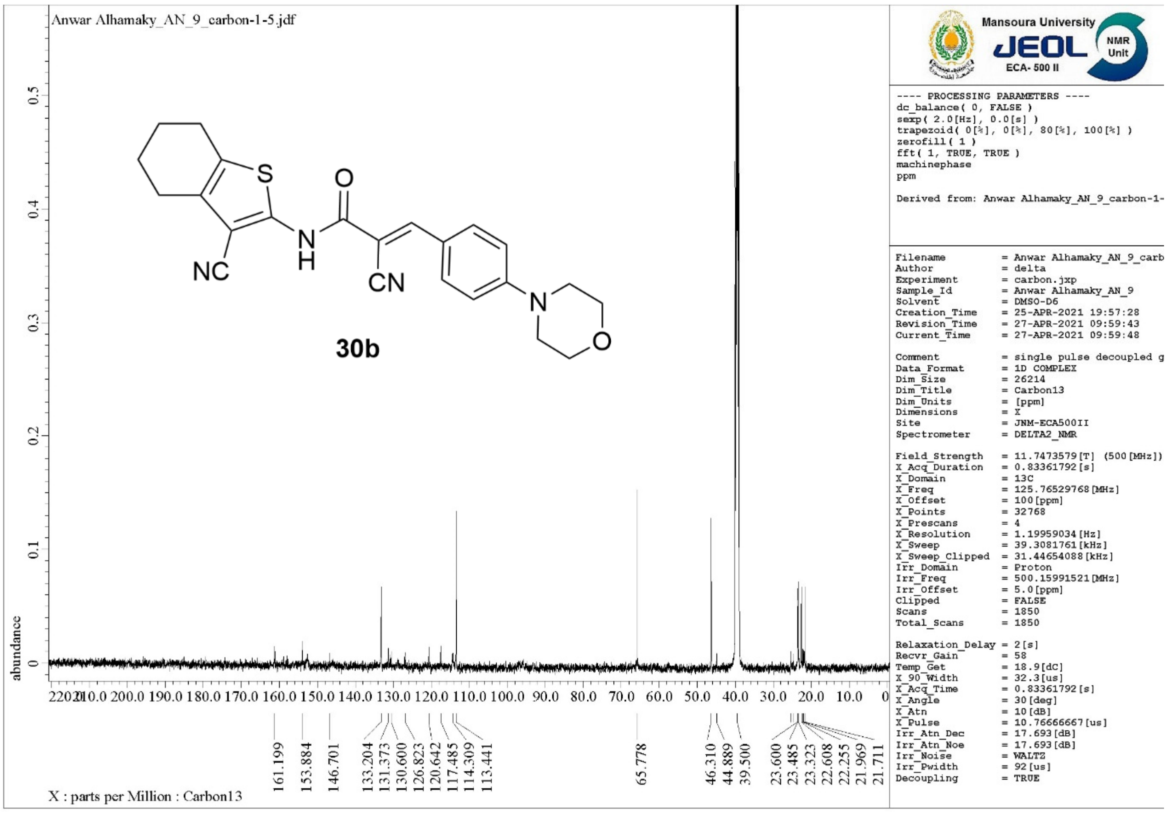

Figure S39. ^{13}C NMR (125 MHz, DMSO-d_6) spectrum of compound **30b** in *E* configuration.

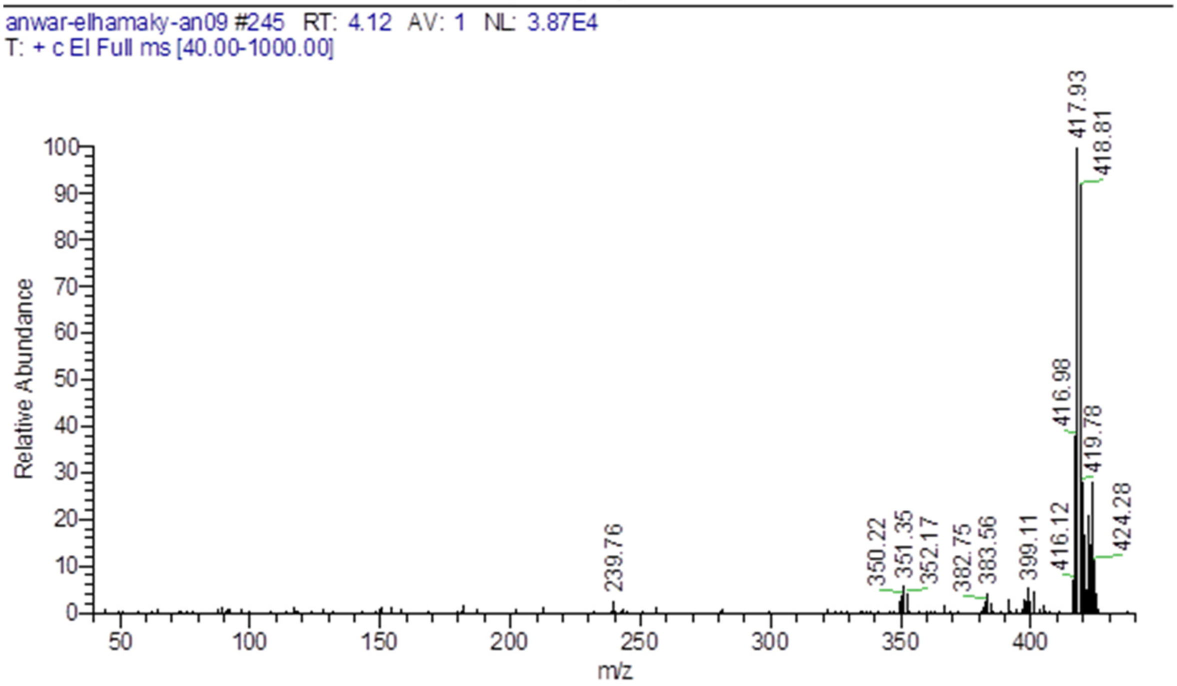

Figure S40. Mass spectrum of compound **30b**.

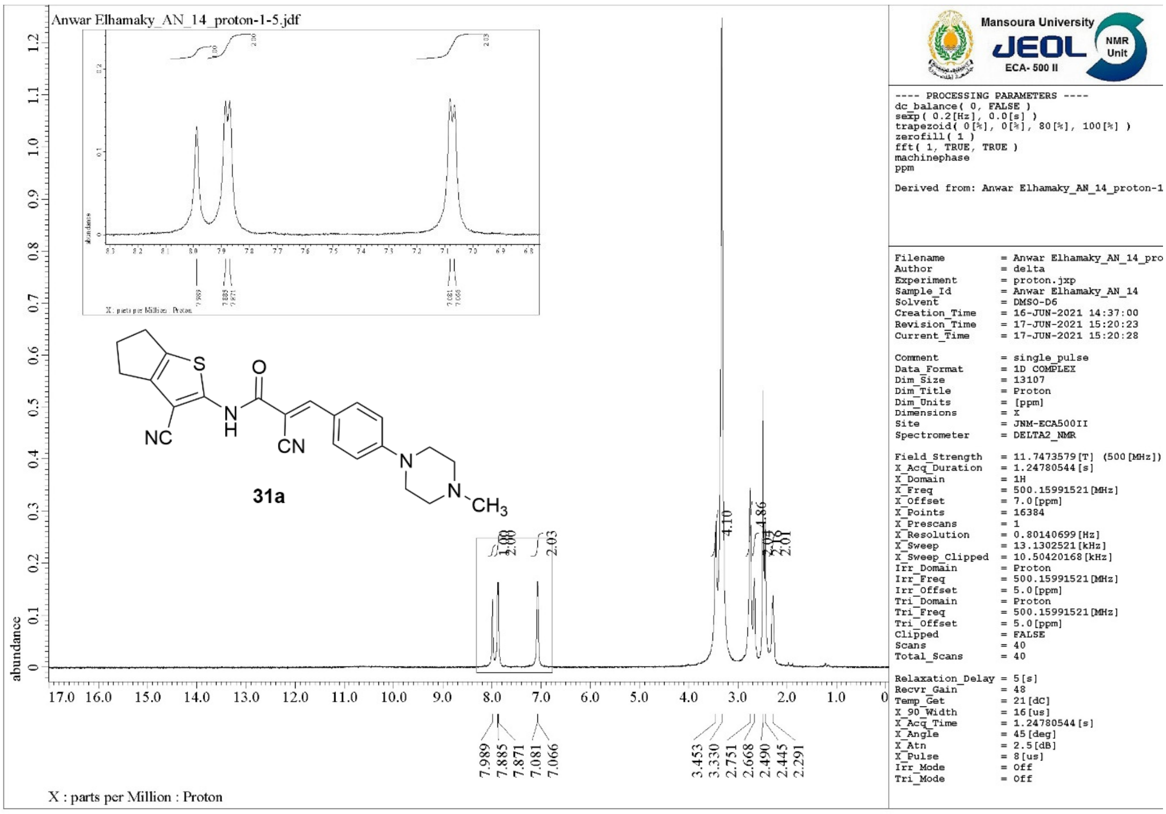

Figure S41. ^{1}H NMR (500 MHz, DMSO-d_6) spectrum of compound **31a** in *E* configuration.

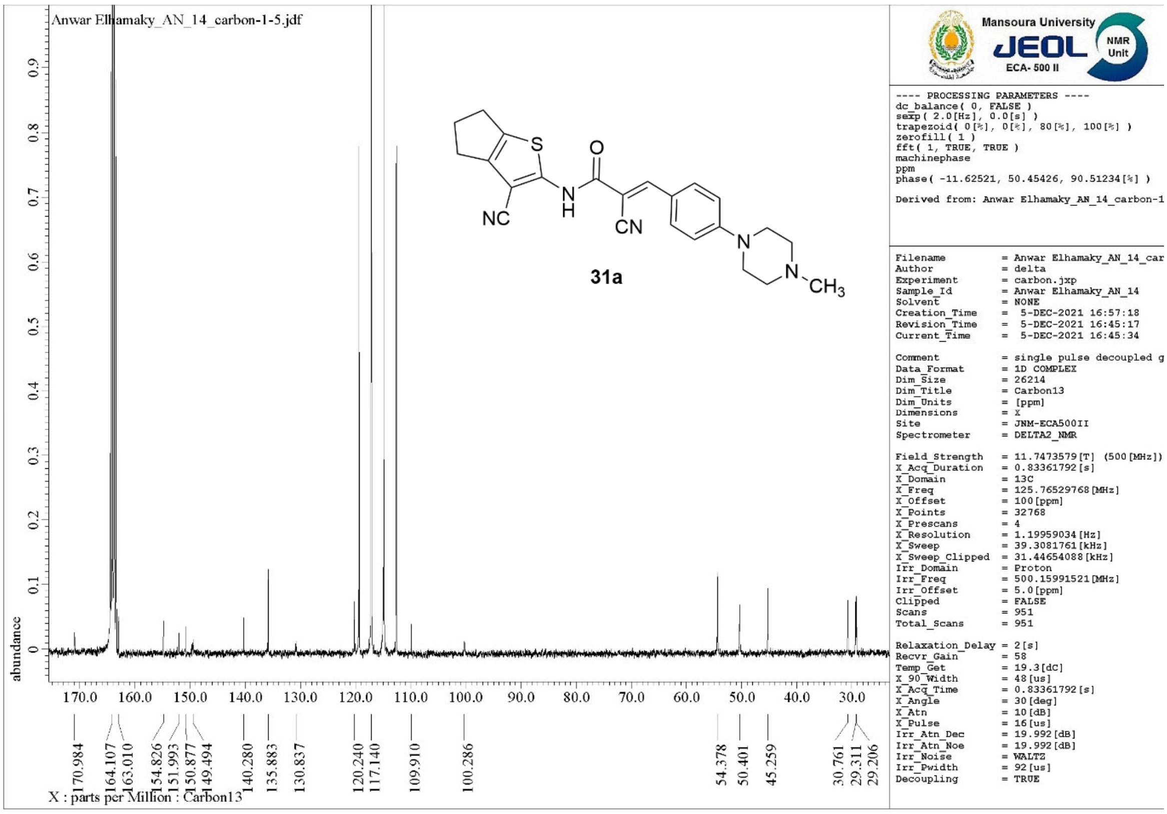

Figure S42. ^{13}C NMR (125 MHz, CF_3COOD) spectrum of compound **31a** in *E* configuration.

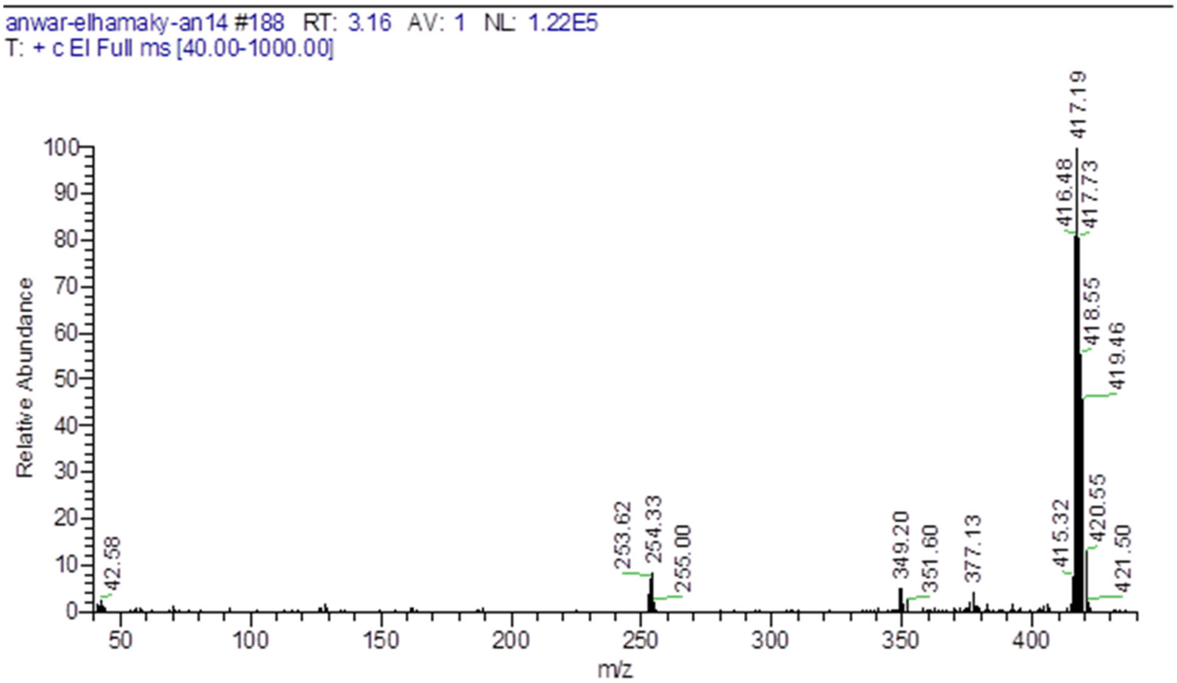

Figure S43. Mass spectrum of compound **31a**.

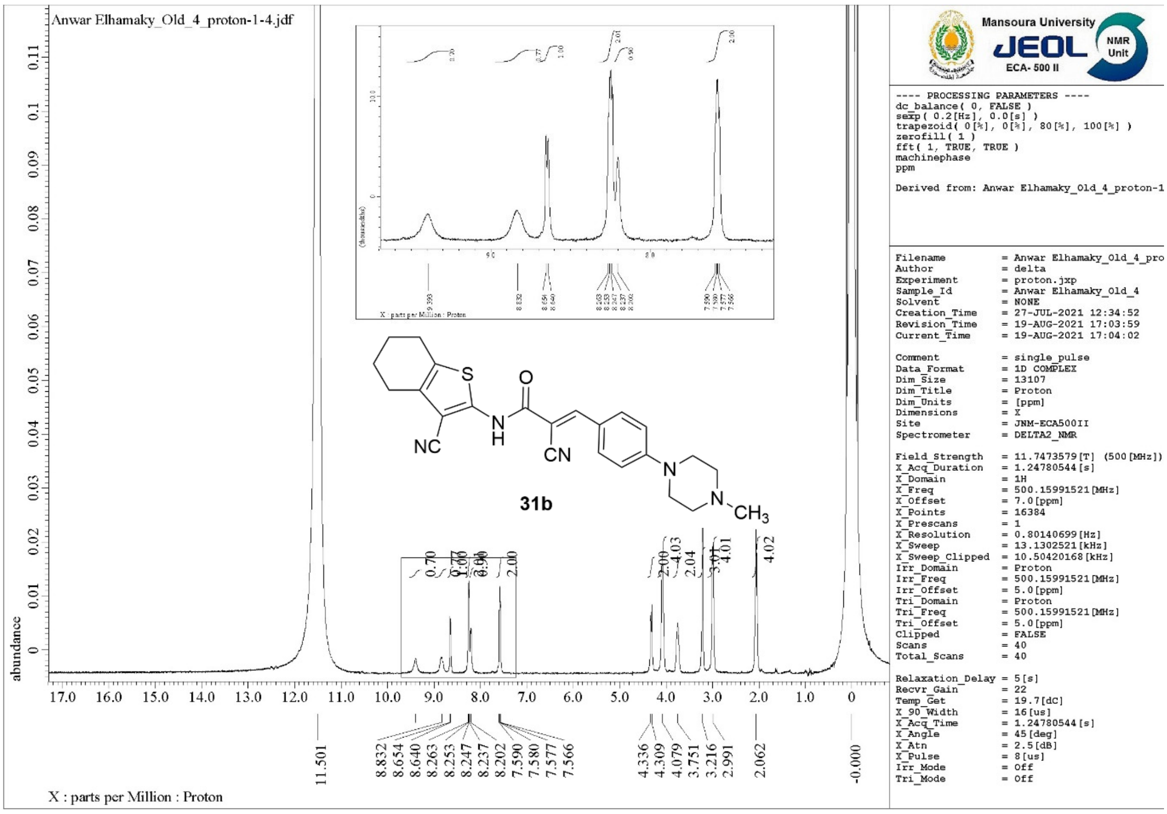

Figure S44. ^{1}H NMR (500 MHz, CF_3COOD) spectrum of directly precipitated powder of compound 31**b** in *E* configuration.

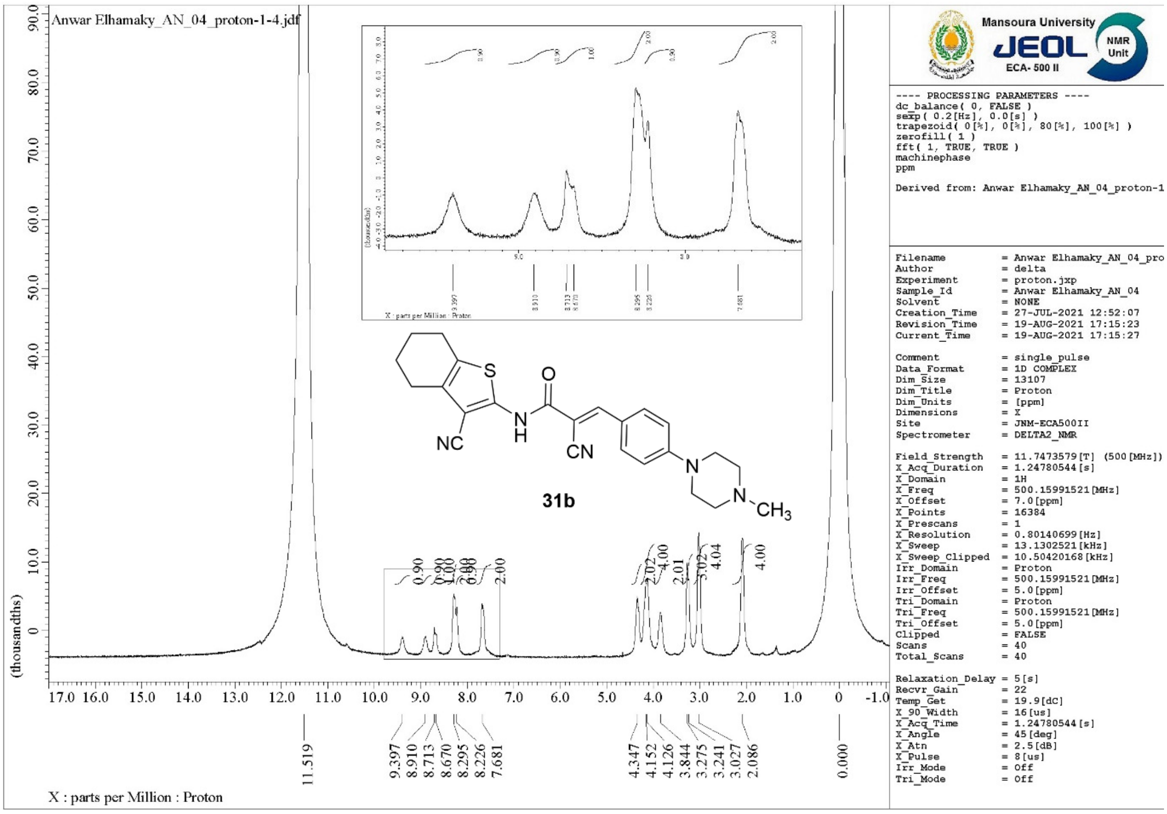

Figure S45. ^{1}H NMR (500 MHz, CF_3COOD) spectrum for the powder obtained by extraction of compound **31b** in *E* configuration.

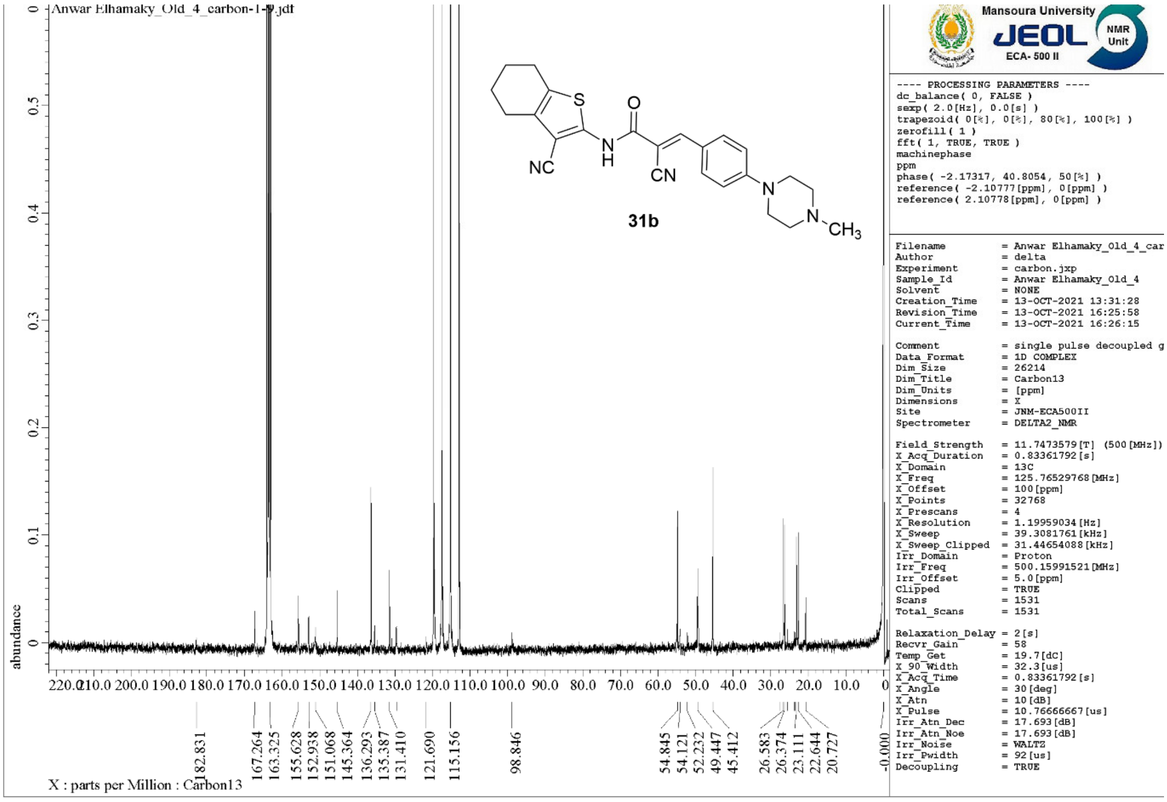

Figure S46. ^{13}C NMR (125 MHz, CF_3COOD) spectrum of compound **31b**.

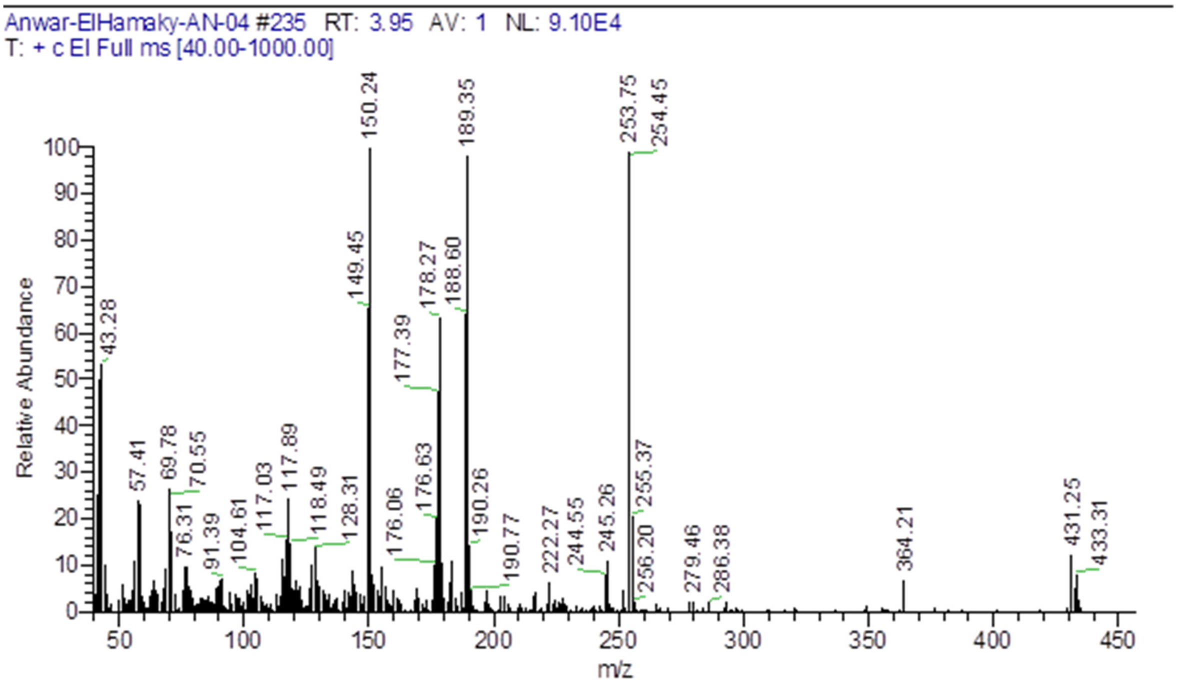

Figure S47. Mass spectrum of compound **31b**.

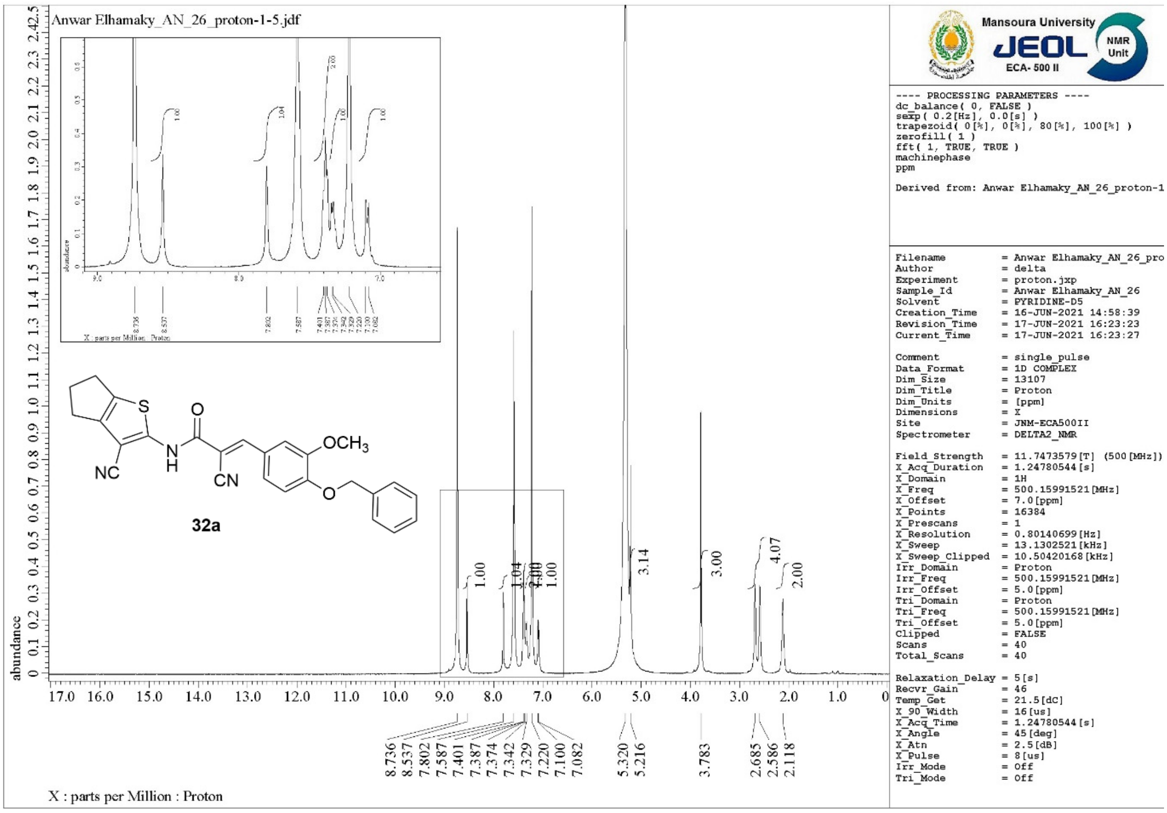

Figure S48. ^{1}H NMR (500 MHz, pyridine-d_5) spectrum of compound **32a** in *E* configuration.

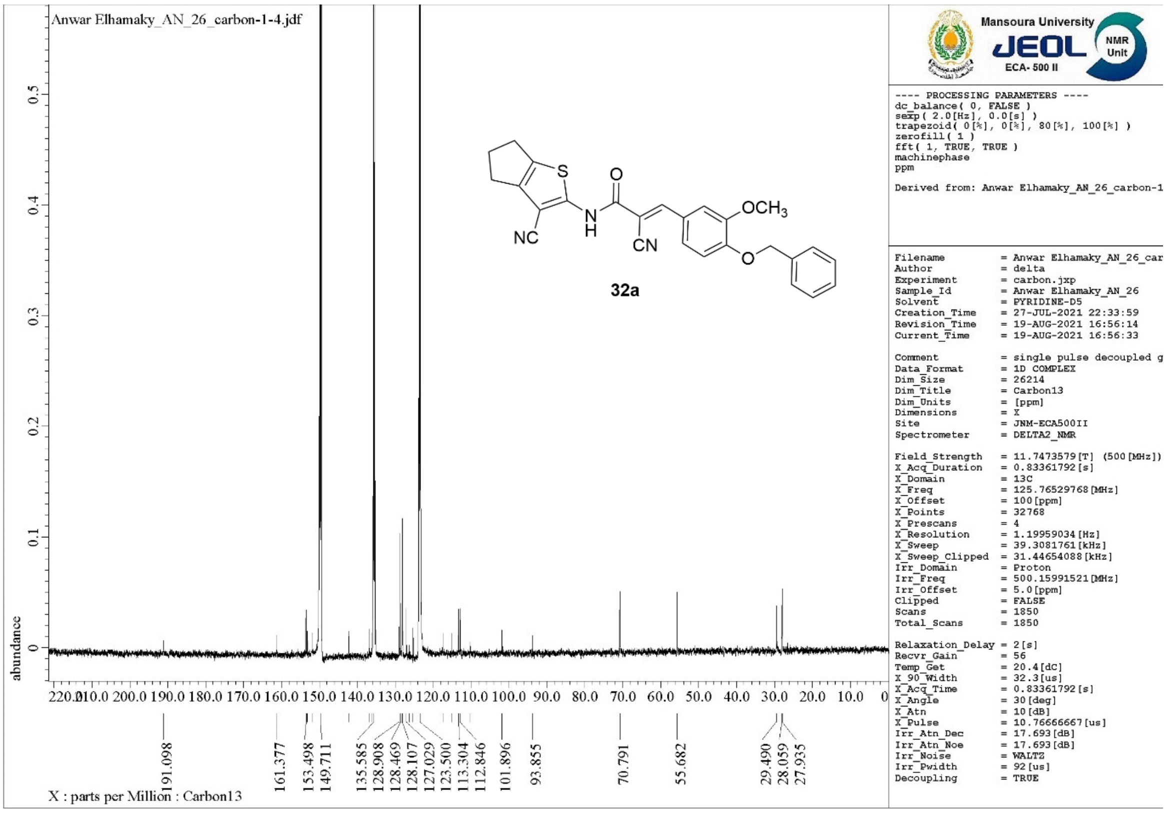

Figure S49. ^{13}C NMR (125 MHz, pyridine-d_5) spectrum of compound **32a**.

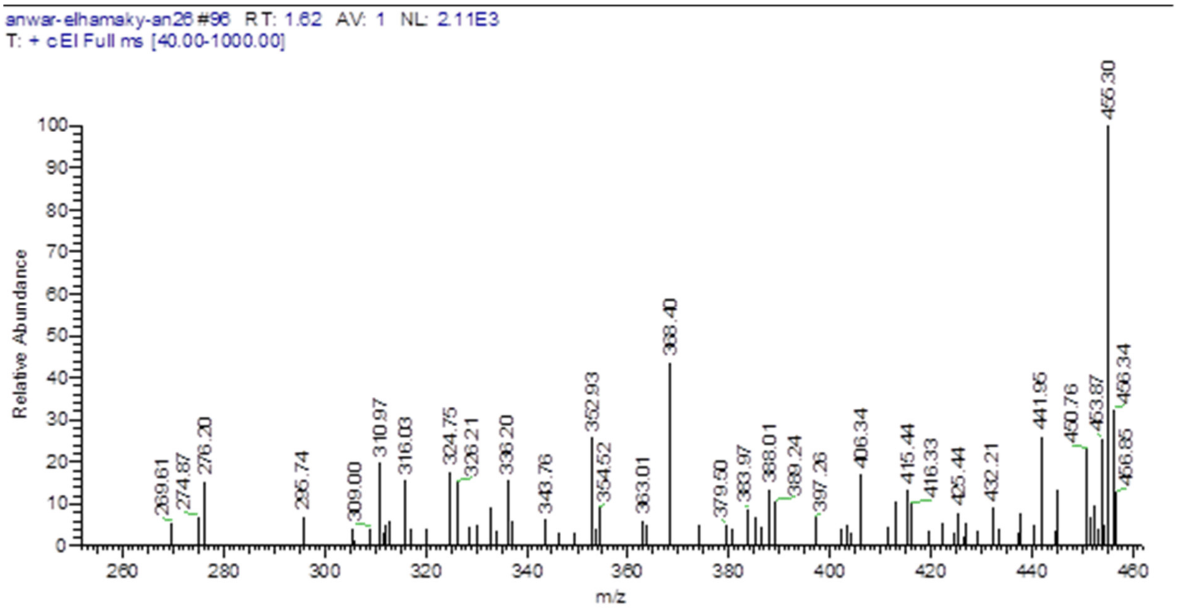

Figure S50. Mass spectrum of compound **32a**.

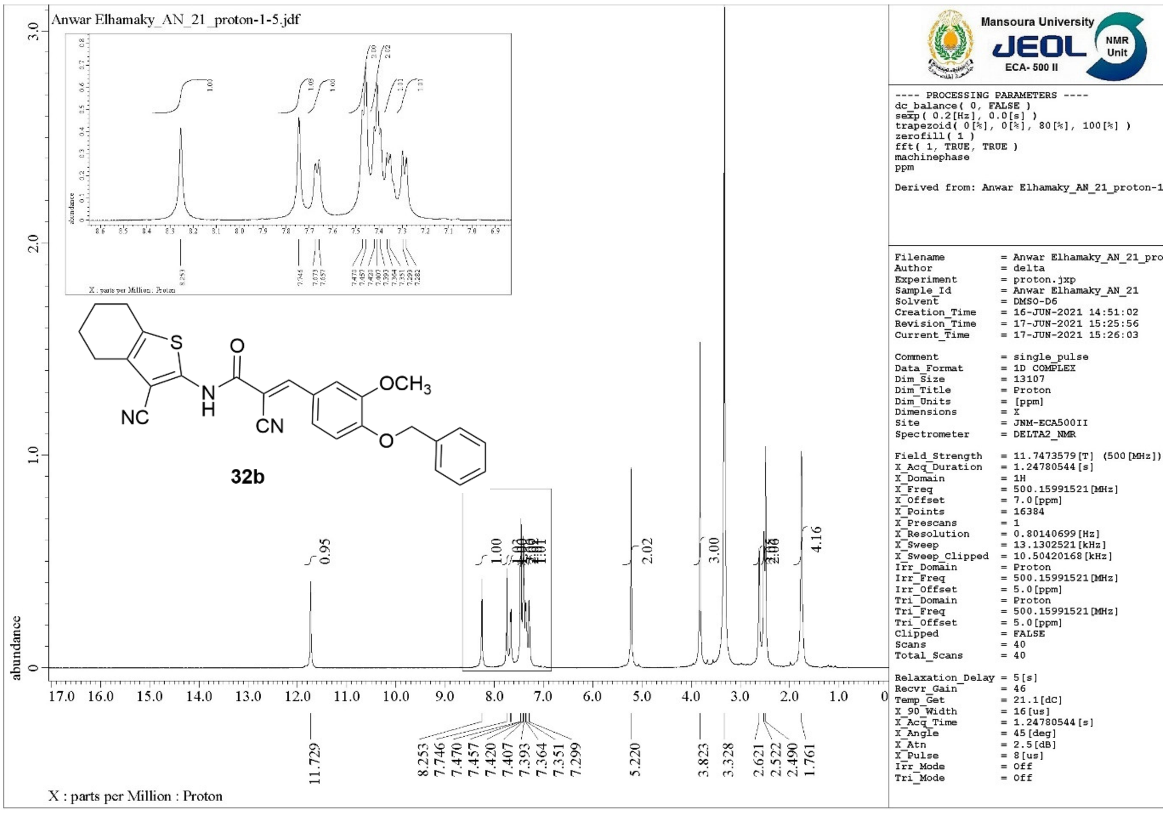

Figure S51. ^{1}H NMR (500 MHz, DMSO-d_6) spectrum of compound **32b** in *E* configuration.

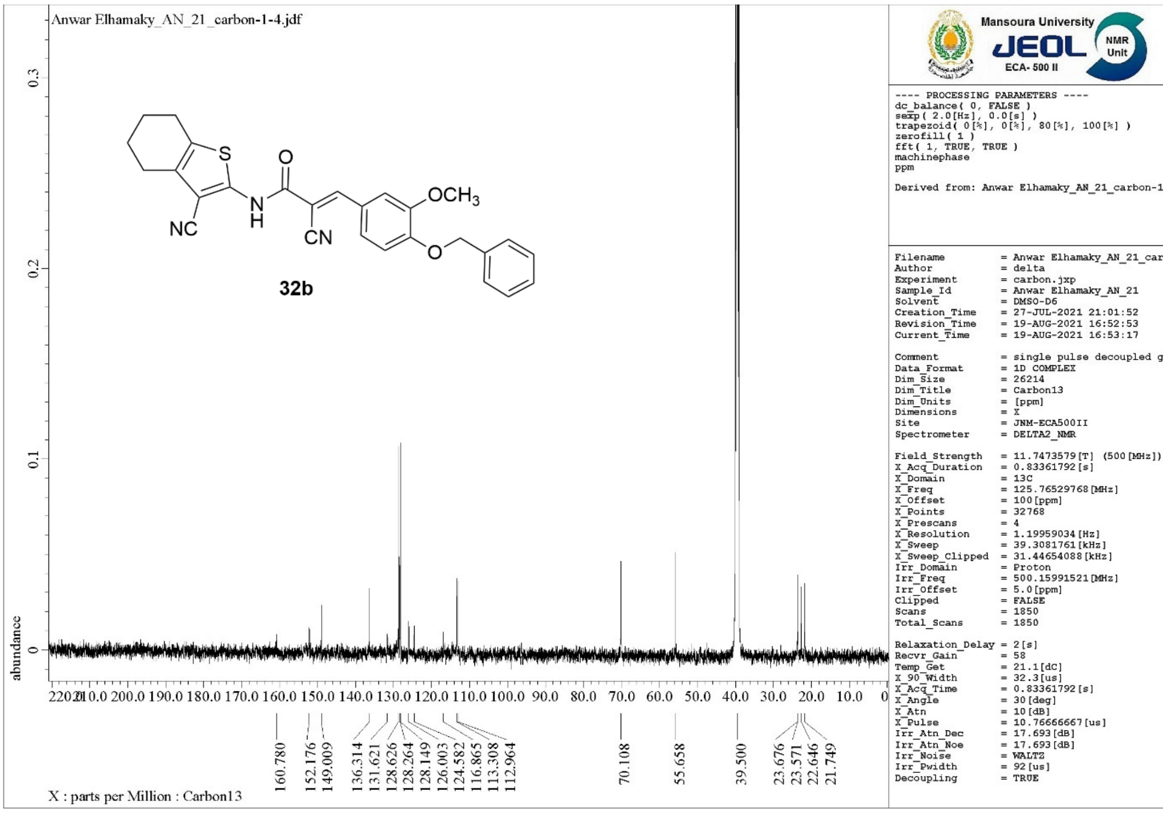

Figure S52. ^{13}C NMR (125 MHz, DMSO-d_6) spectrum of compound **32b**.

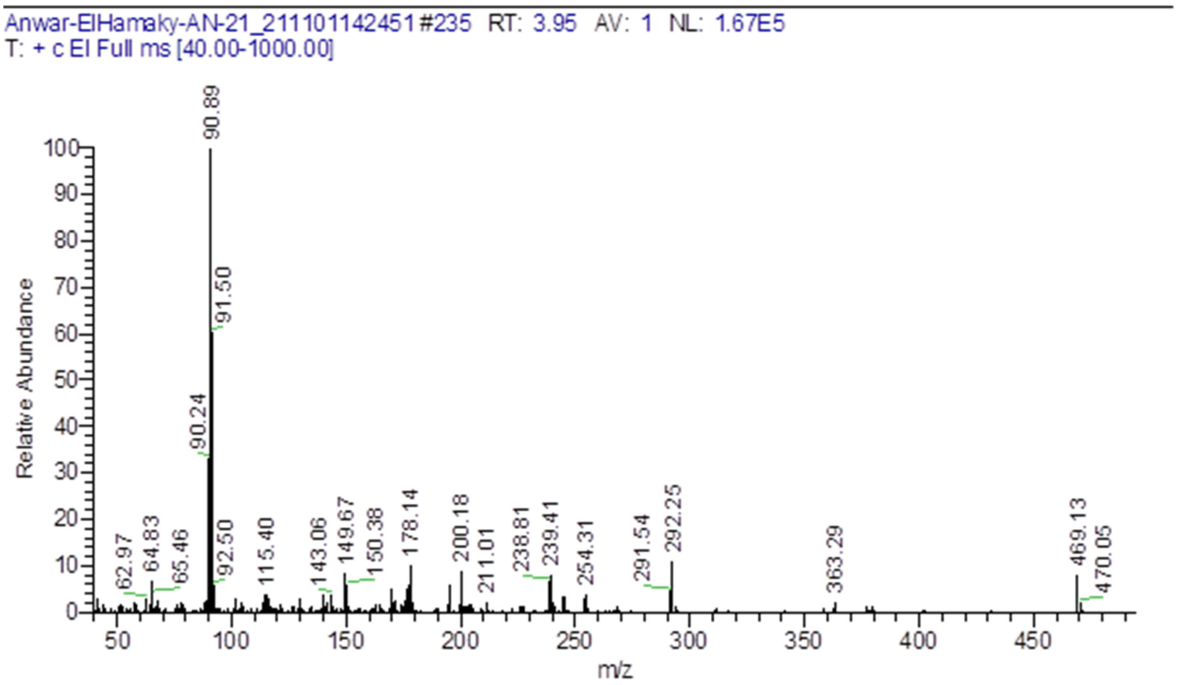

Figure S53. Mass spectrum of compound **32b**.

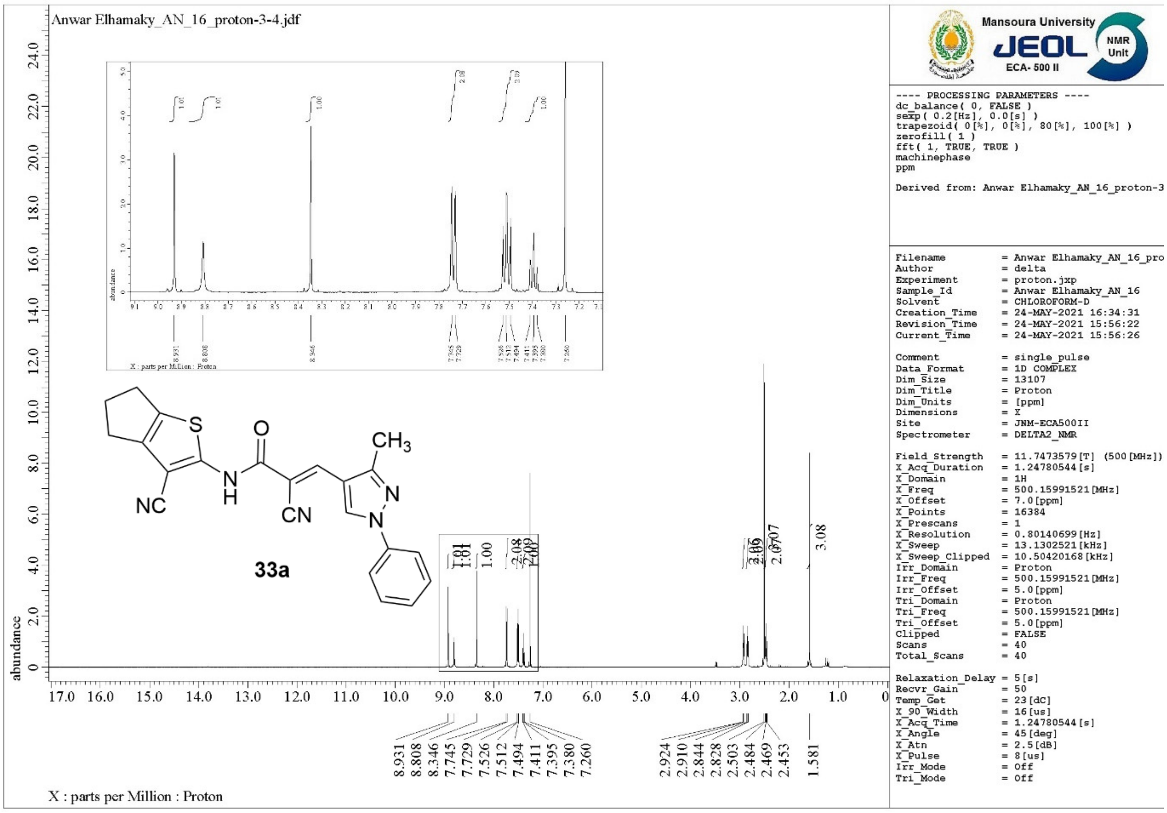

Figure S54. ^{1}H NMR (500 MHz, $CDCl_3$) spectrum of compound **33a** in *E* configuration.

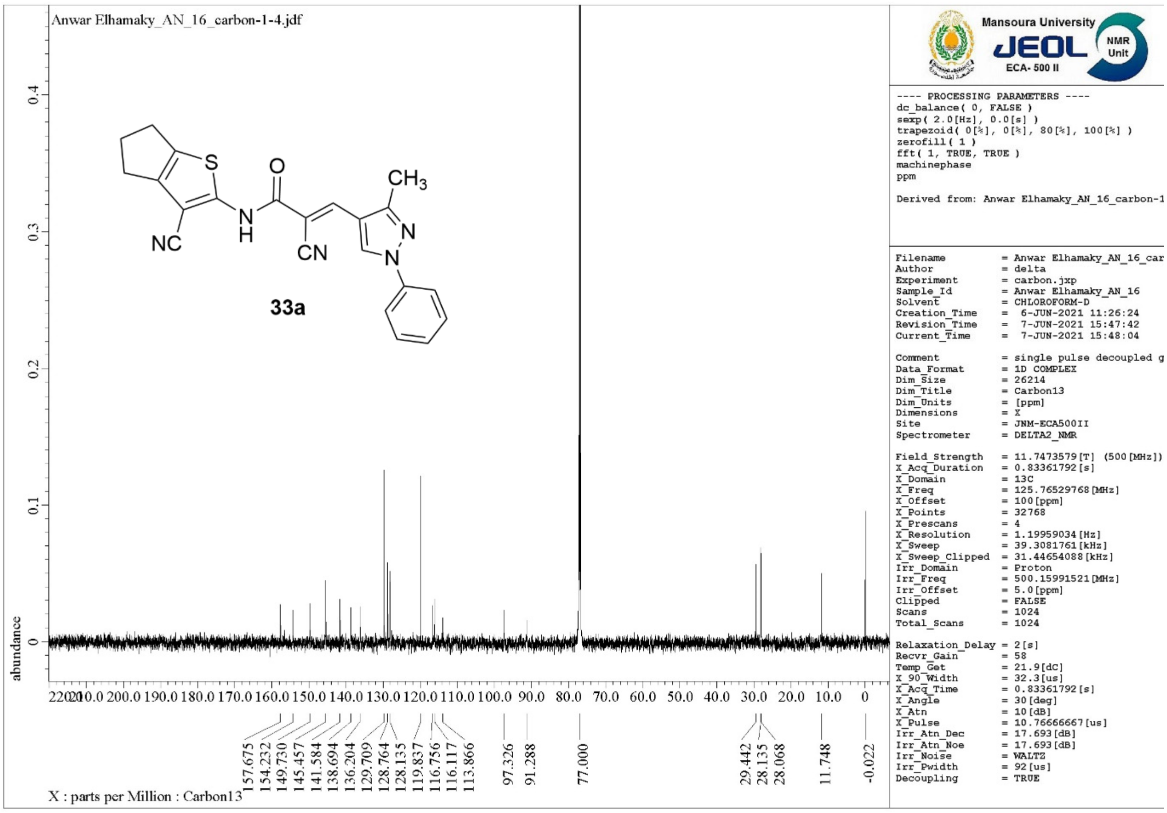

Figure S55. ^{13}C NMR (125 MHz, $CDCl_3$) spectrum of compound **33a**.

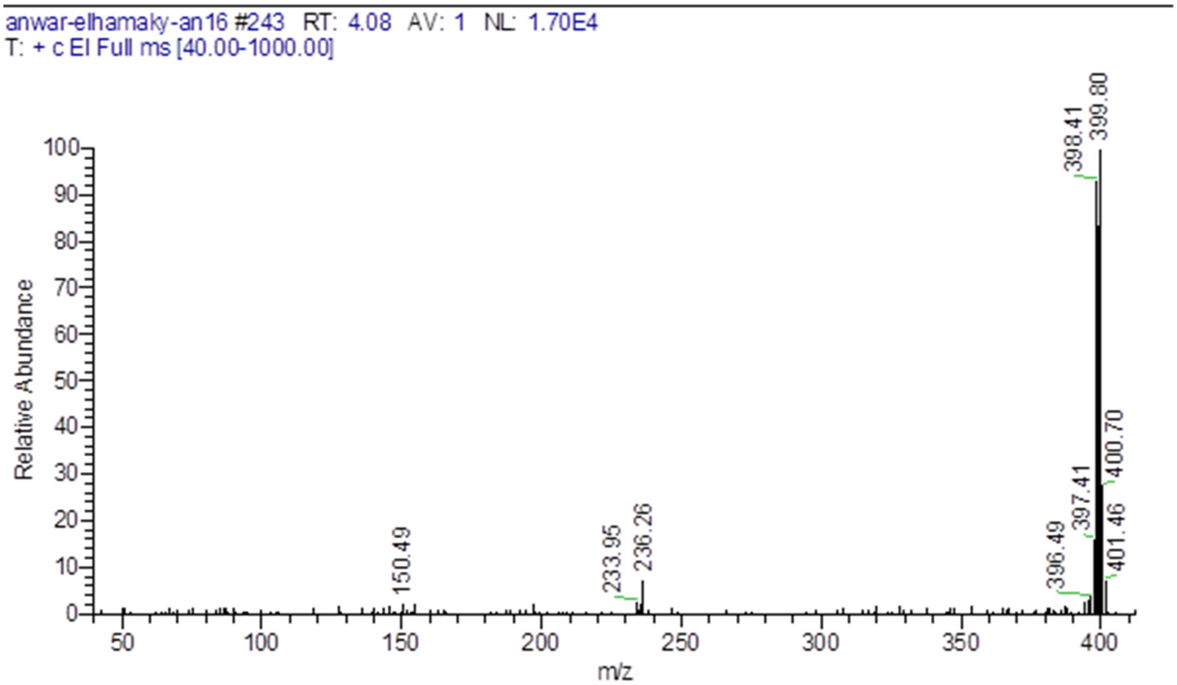

Figure S56. Mass spectrum of compound **33a**.

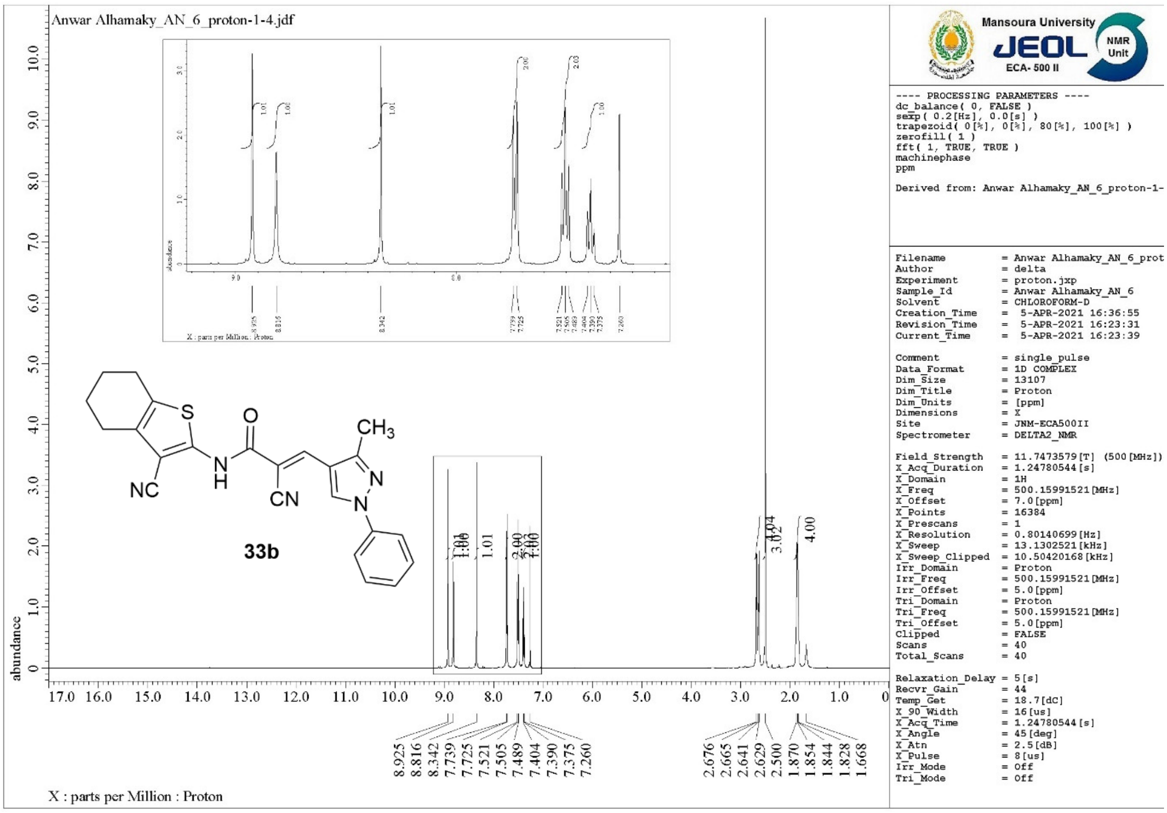

Figure S57. ^{1}H NMR (500 MHz, $CDCl_3$) spectrum of compound **33b** in *E* configuration.

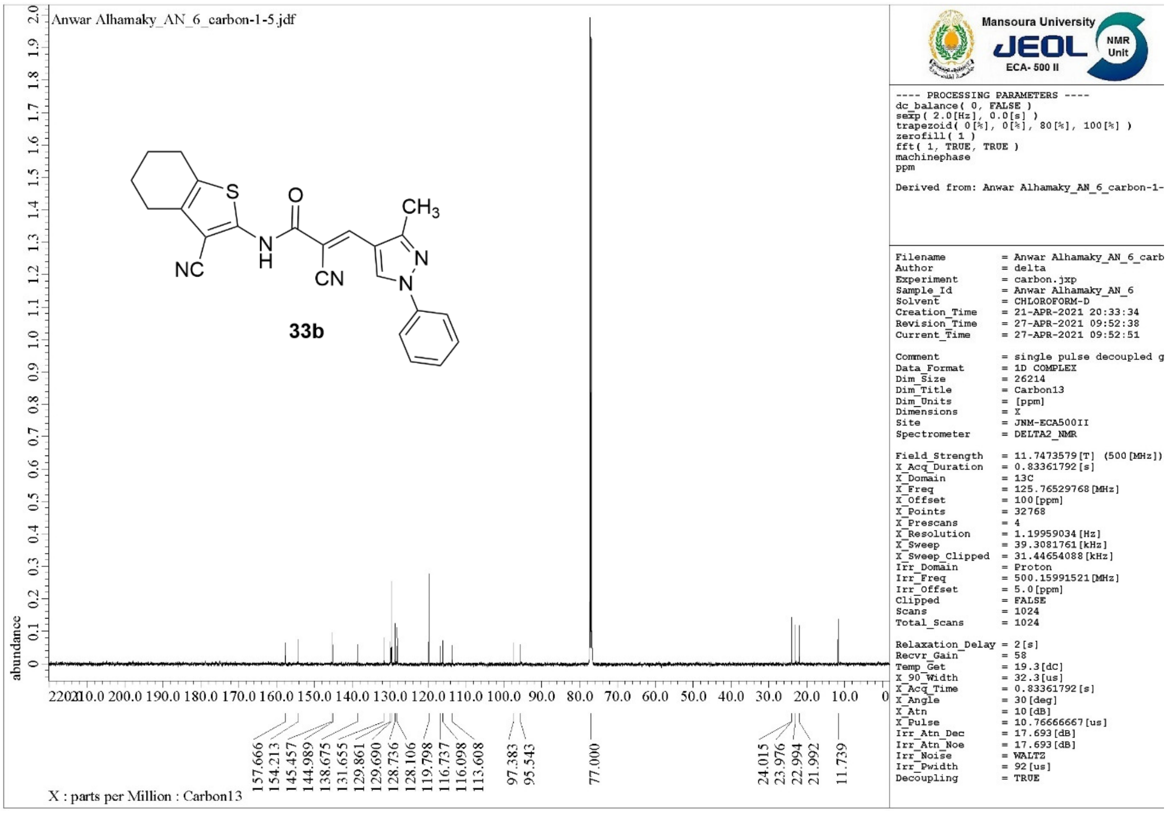

Figure S58. ^{13}C NMR (125 MHz, $CDCl_3$) spectrum of compound **33b**.

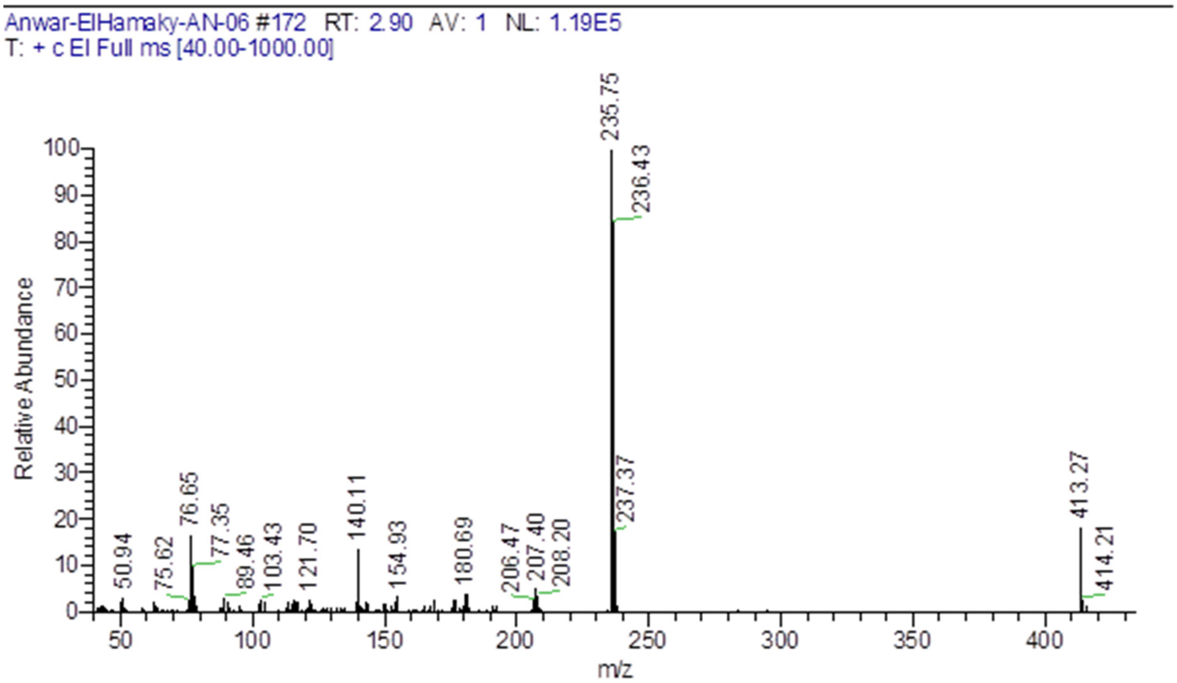

Figure S59. Mass spectrum of compound **33b**.

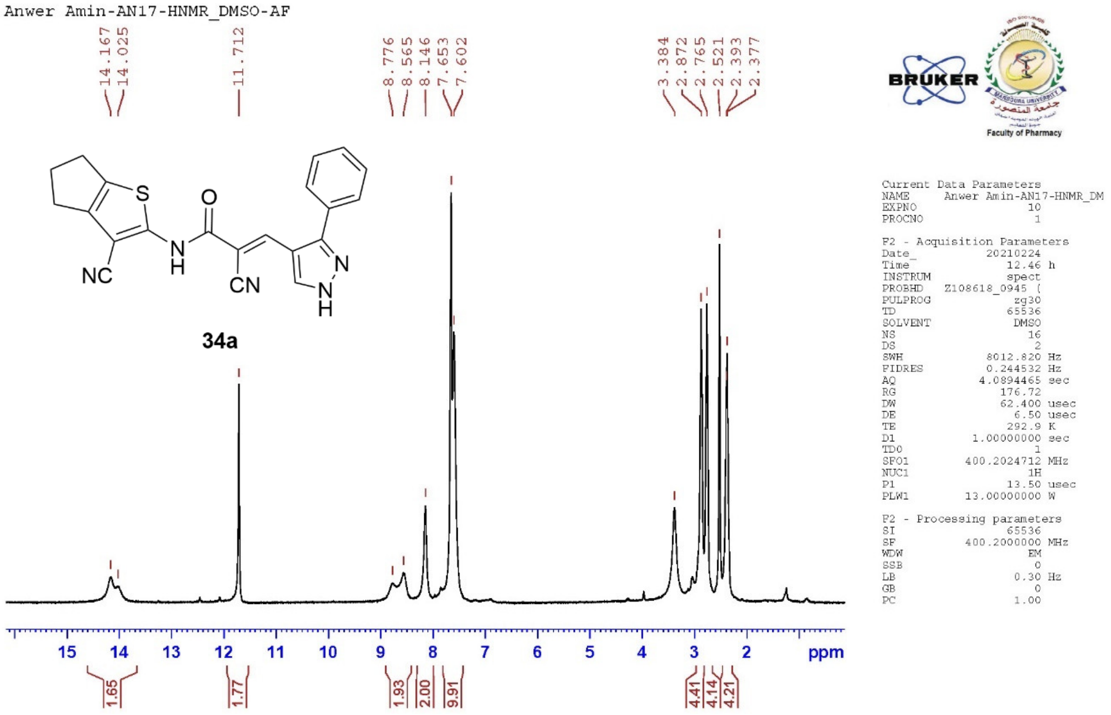

Figure S60. ^{1}H NMR (400 MHz, DMSO-d_6) spectrum of compound **34a** in *E* configuration.

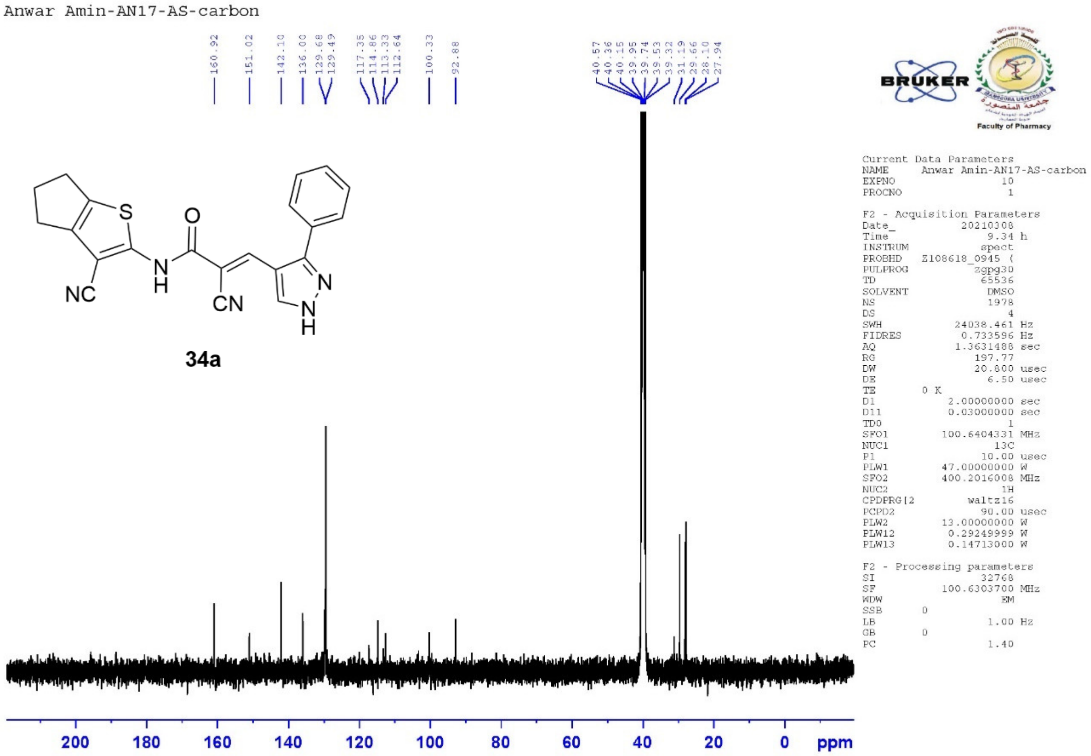

Figure S61. ^{13}C NMR (100 MHz, DMSO-d_6) spectrum of compound **34a**.

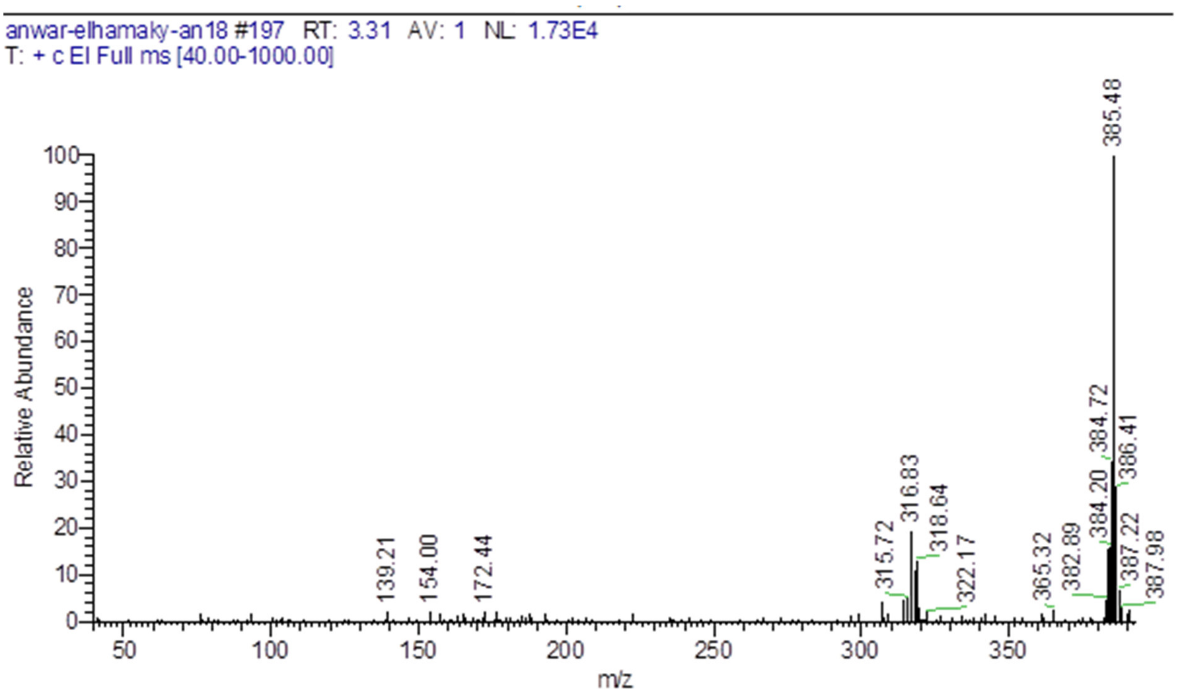

Figure S62. Mass spectrum of compound **34a**.

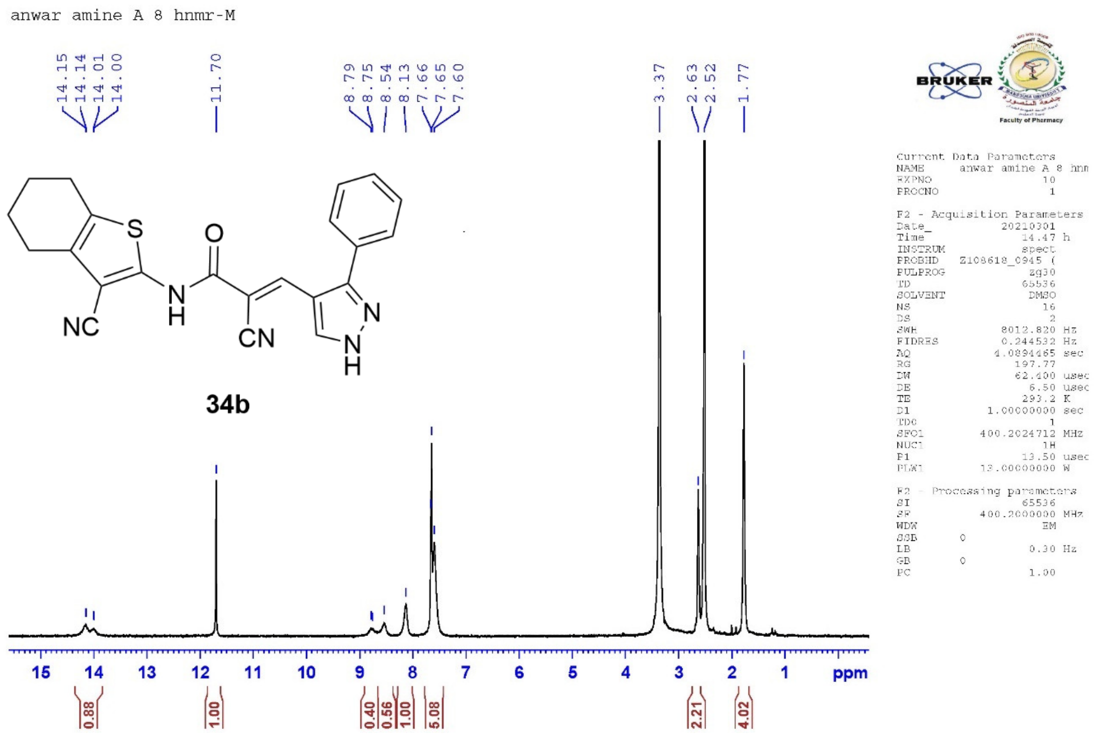

Figure S63. ^{1}H NMR (400 MHz, DMSO-d_6) spectrum of compound **34b** in *E* configuration.

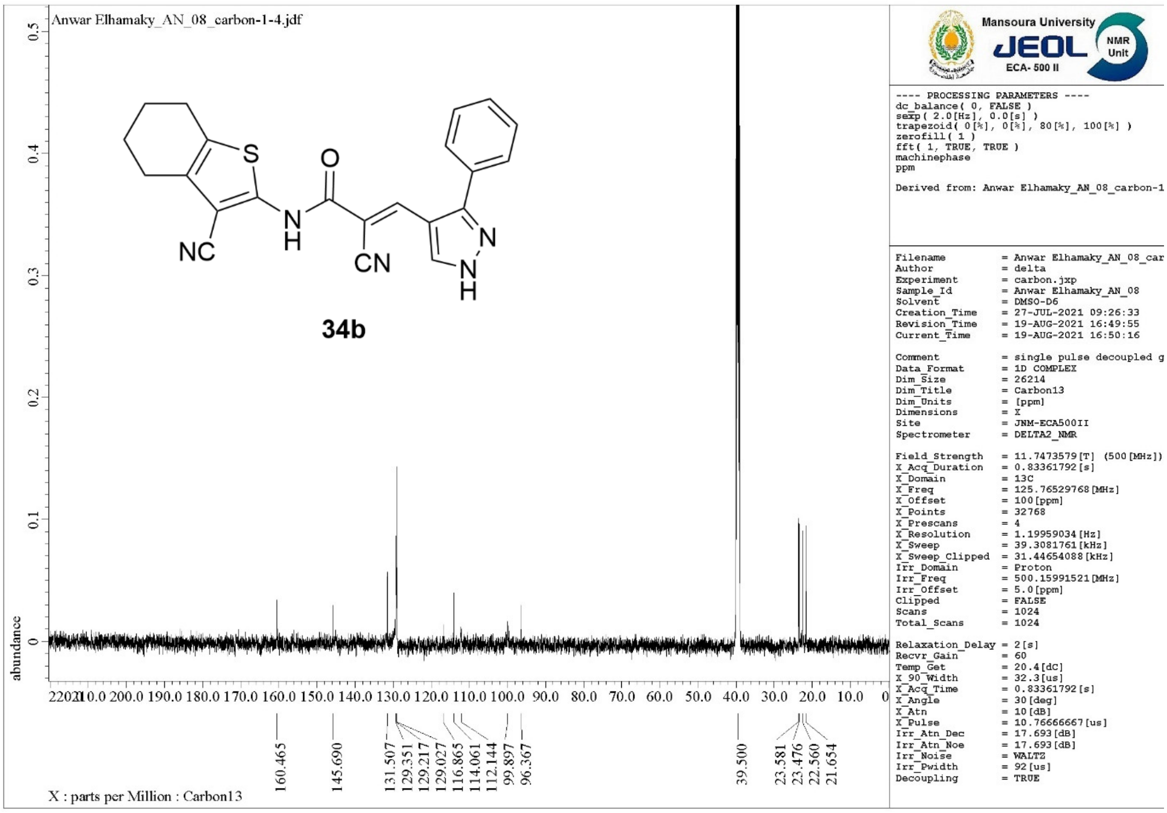

Figure S64. ^{13}C NMR (125 MHz, DMSO-d_6) spectrum of compound **34b**.

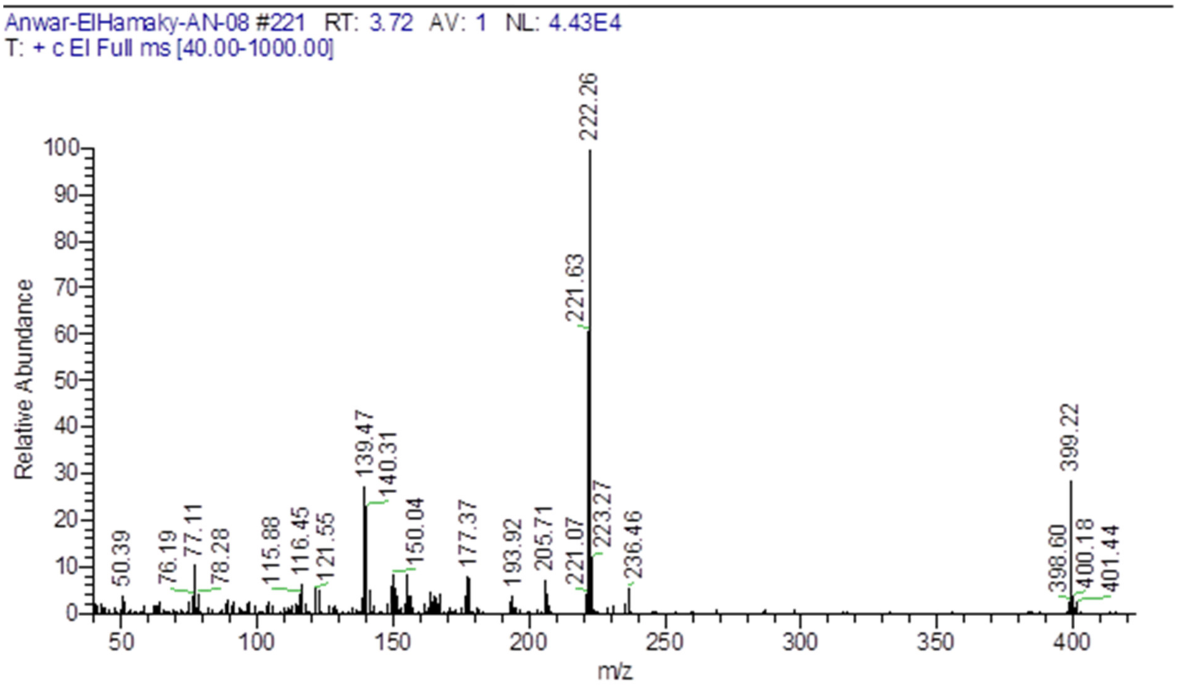

Figure S65. Mass spectrum of compound **34b**.

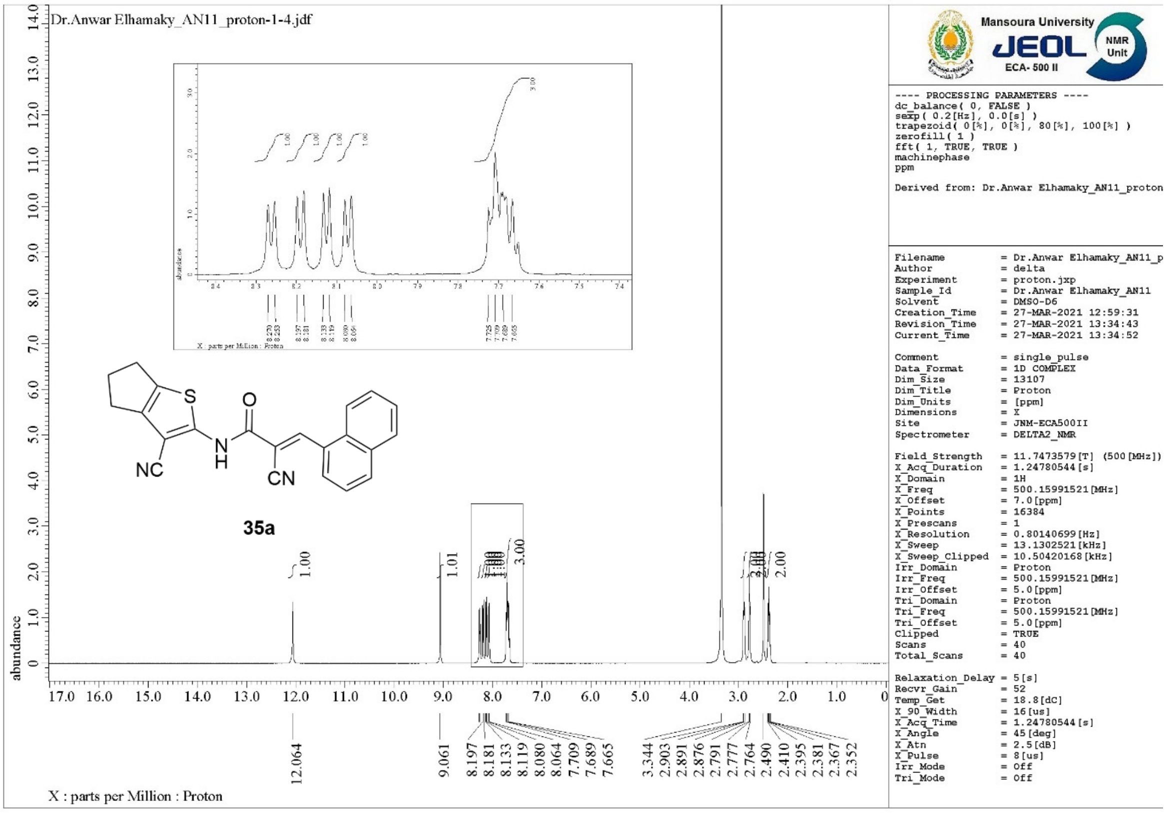

Figure S66. ^{1}H NMR (500 MHz, DMSO-d_6) spectrum of compound **35a** in *E* configuration.

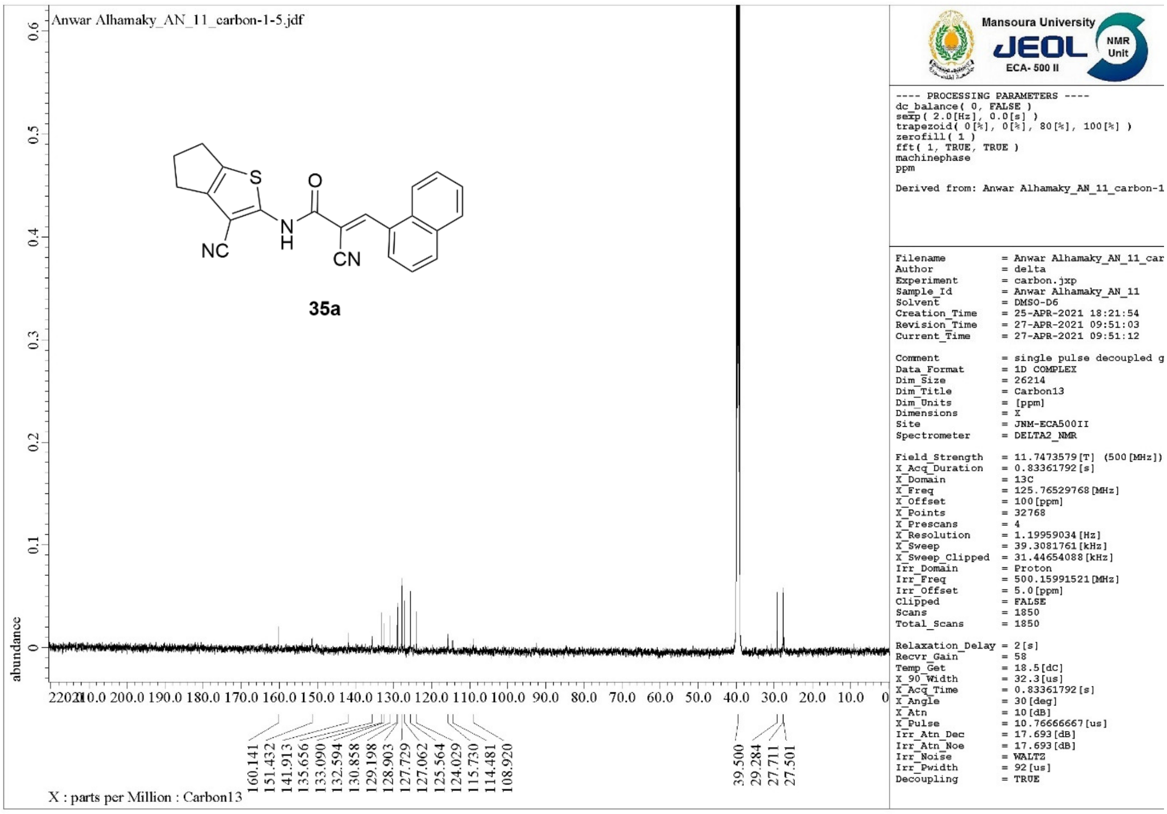

Figure S67. ^{13}C NMR (125 MHz, DMSO-d_6) spectrum of compound **35a**.

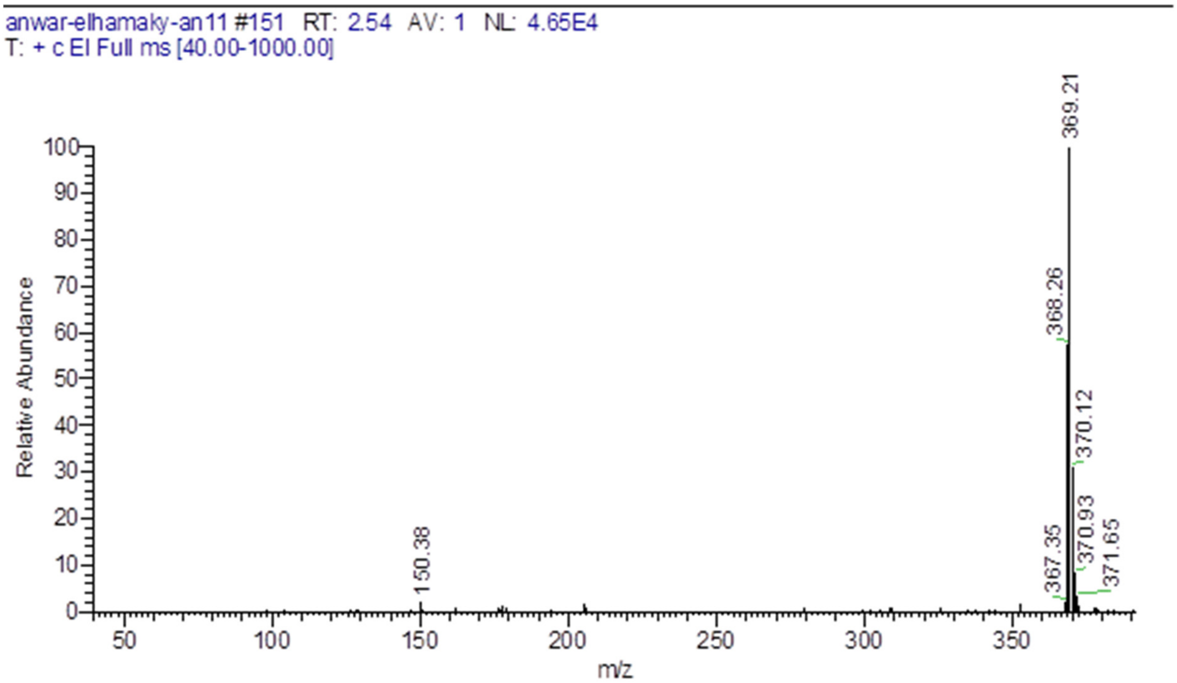

Figure S68. Mass spectrum of compound **35a**.

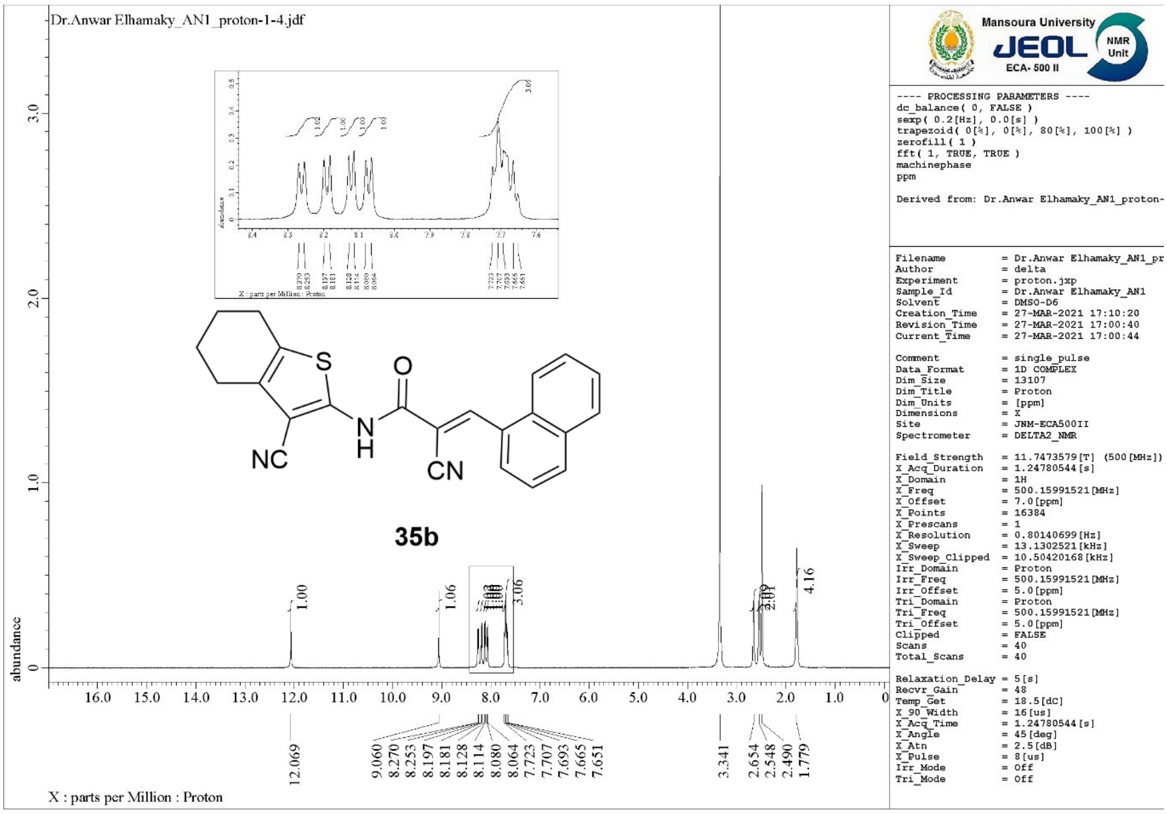

Figure S69. ^{1}H NMR (500 MHz, DMSO-d_6) spectrum of compound **35b** in *E* configuration.

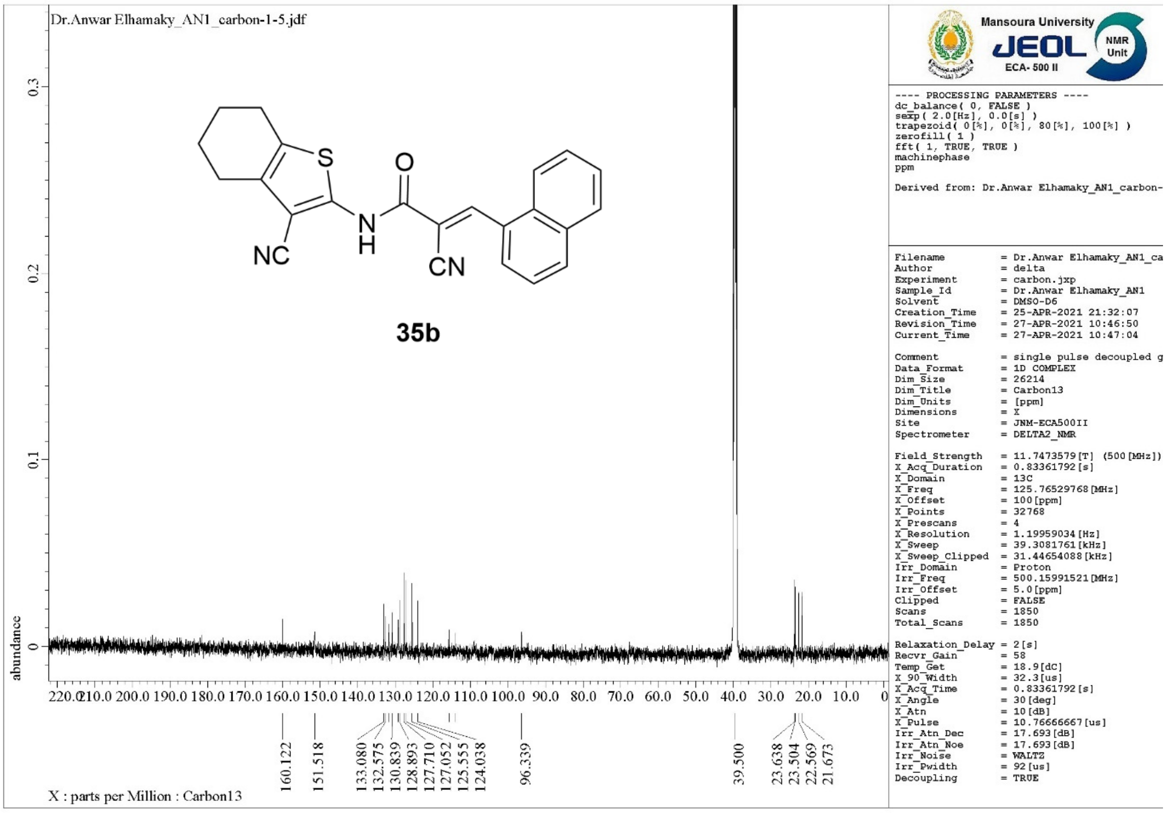

Figure S70. ^{13}C NMR (125 MHz, DMSO-d_6) spectrum of compound **35b**.

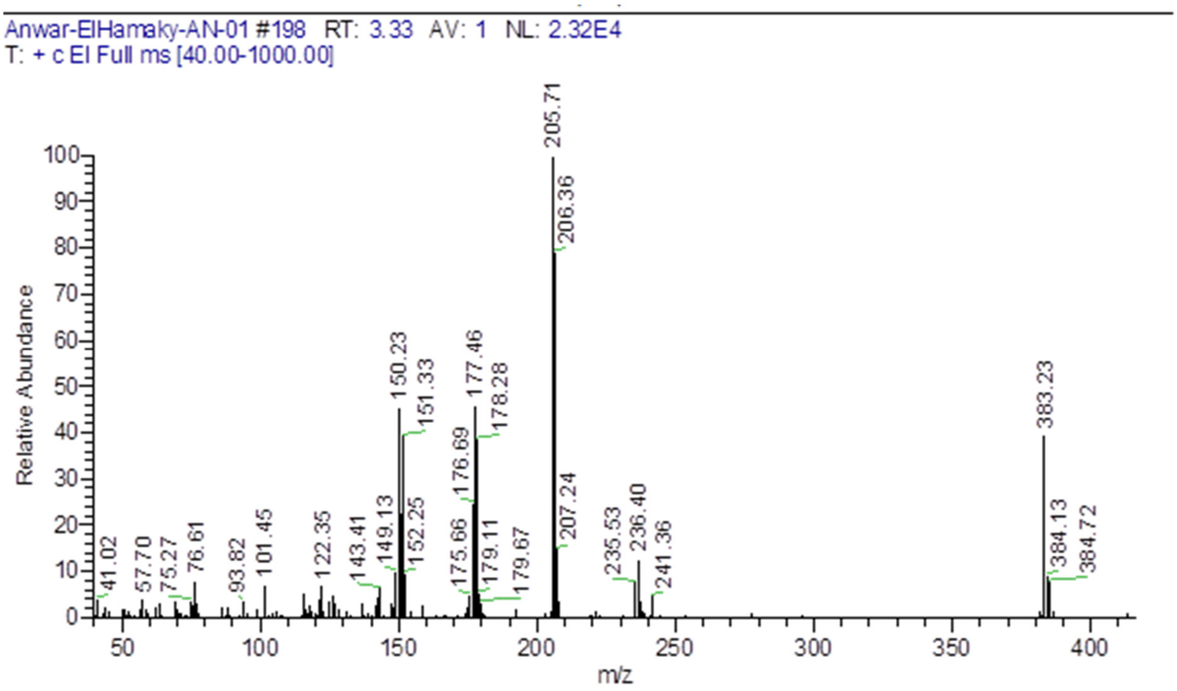

Figure S71. Mass spectrum of compound **35b**.

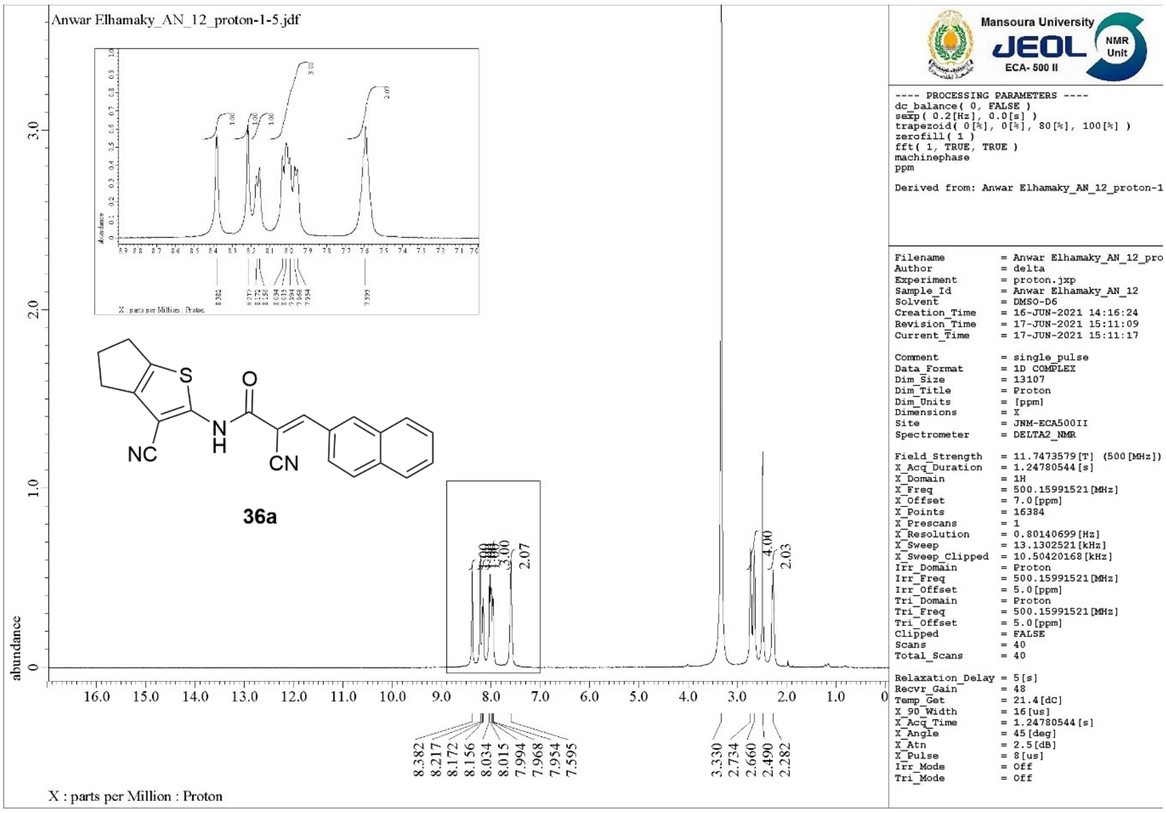

Figure S72. ^{1}H NMR (500 MHz, DMSO-d_6) spectrum of compound **36a** in *E* configuration.

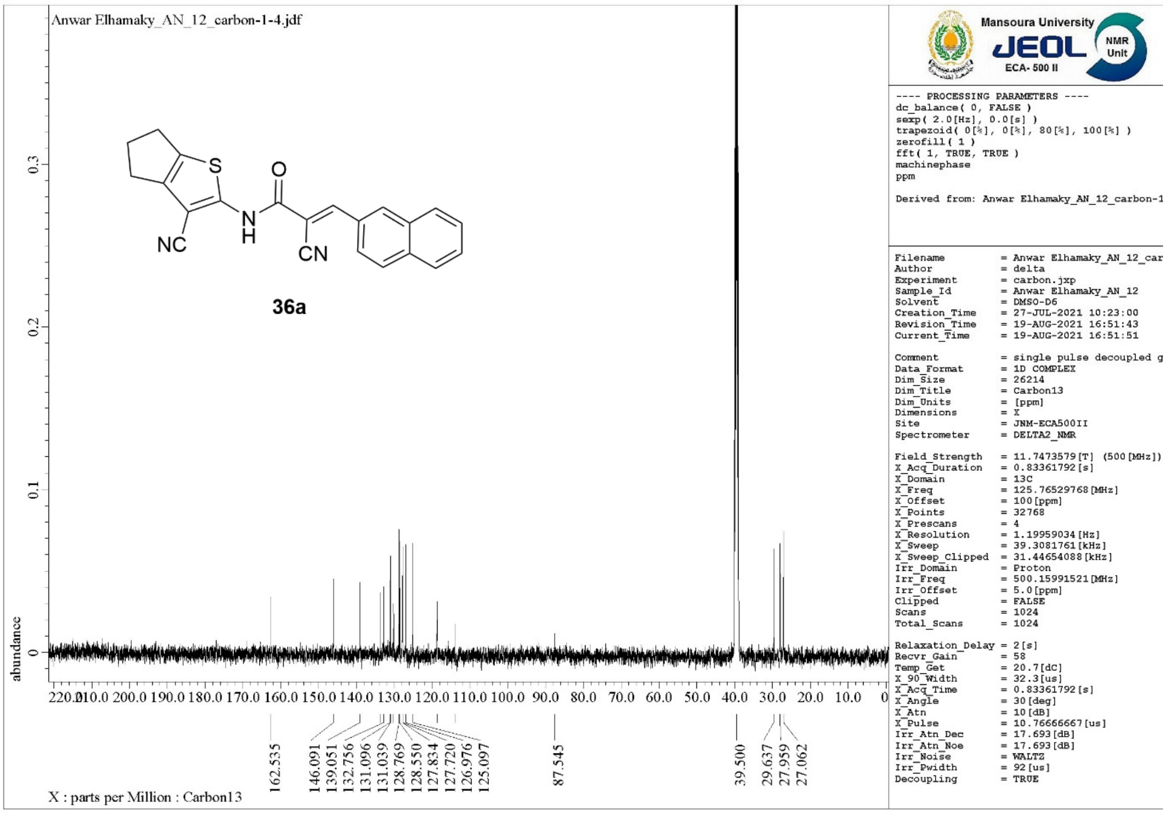

Figure S73. ^{13}C NMR (125 MHz, DMSO-d_6) spectrum of compound **36a**.

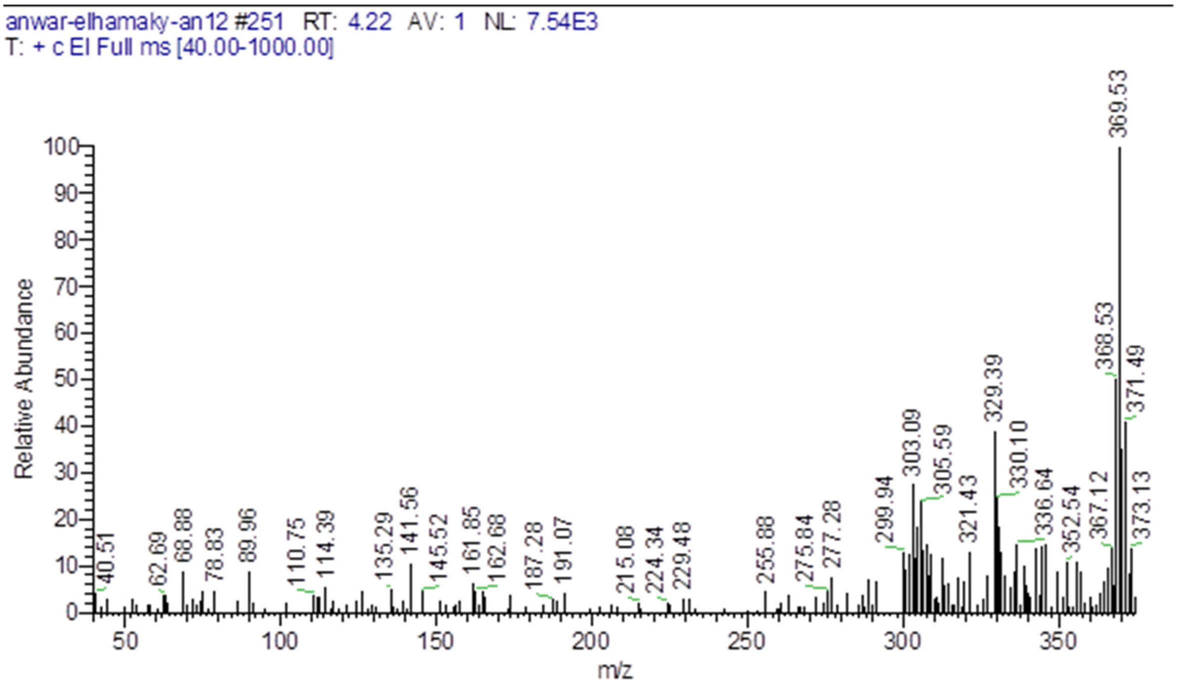

Figure S74. Mass spectrum of compound **36a**.

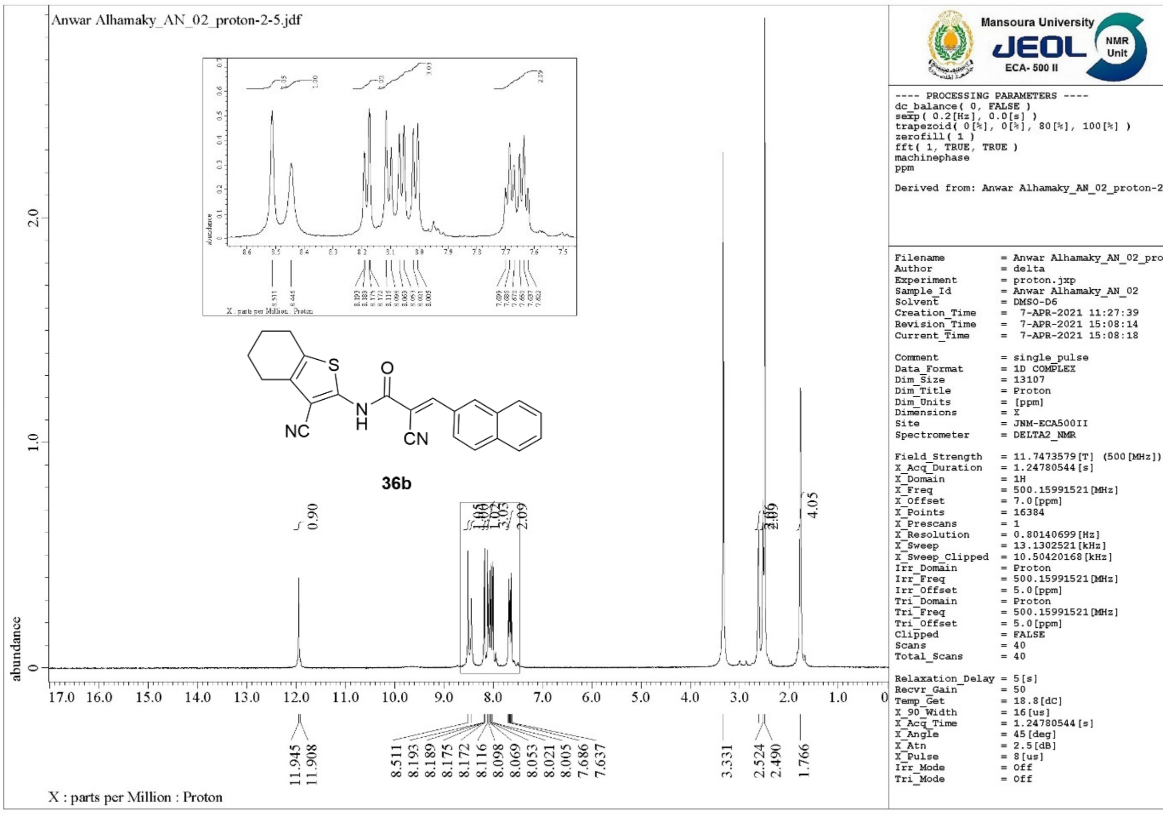

Figure S75. ^{1}H NMR (500 MHz, DMSO-d_6) spectrum of compound **36b** in *E* configuration.

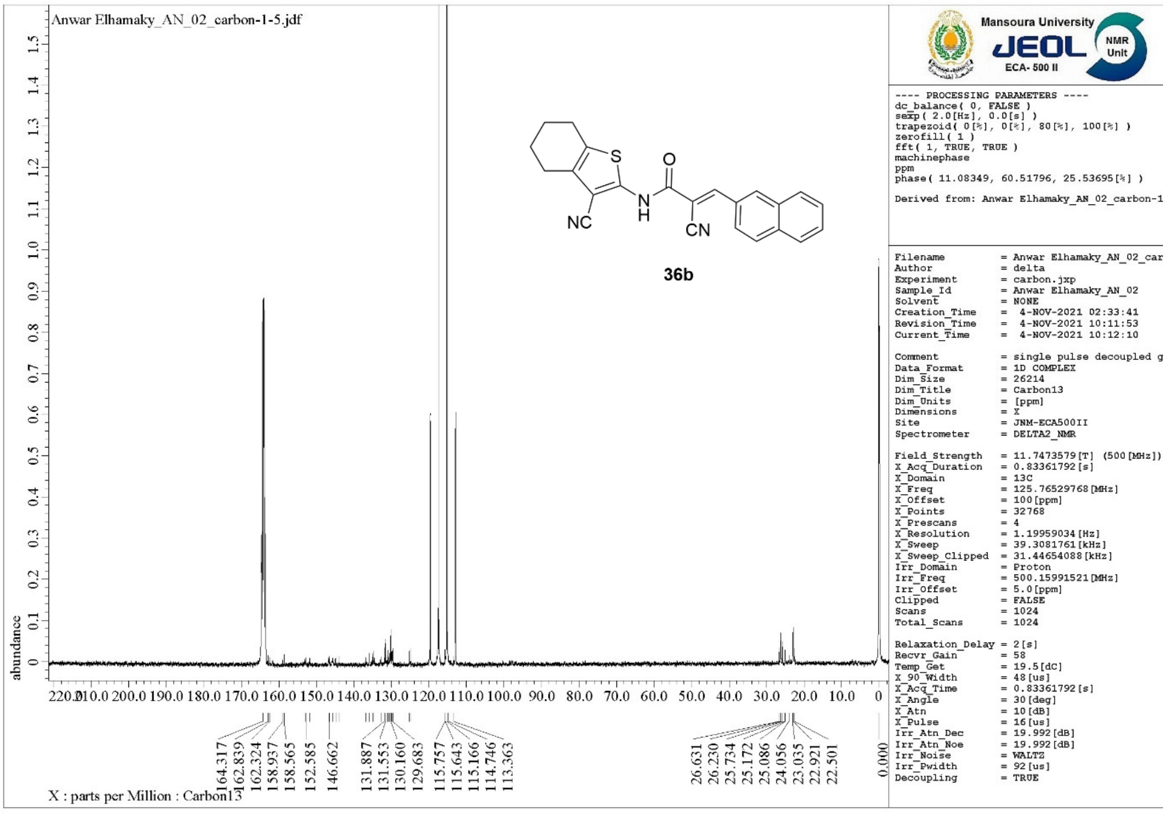

Figure S76 a. ^{13}C NMR (125 MHz, CF_3COOD) spectrum of compound **36b**.

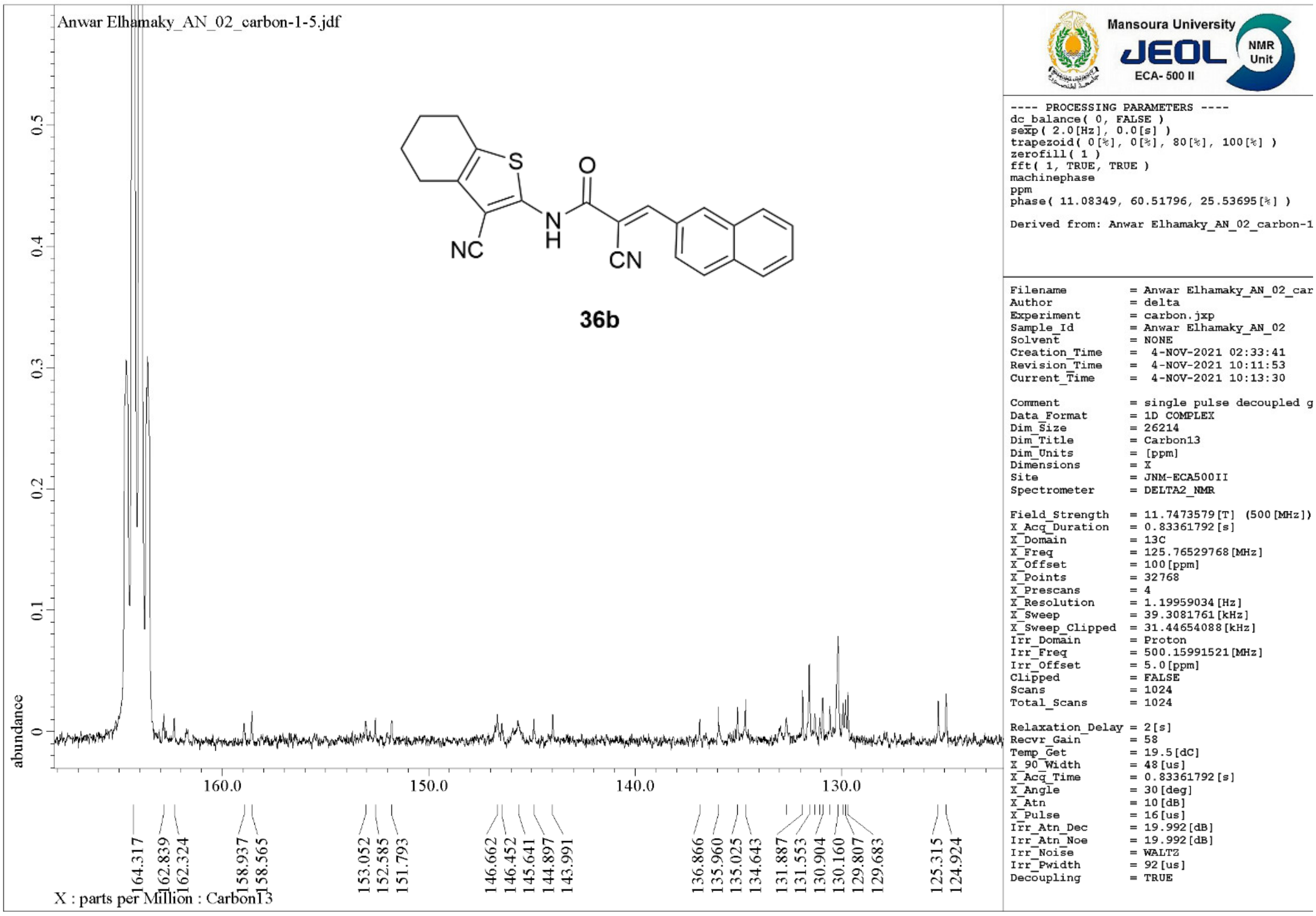

Figure S76 b. ^{13}C NMR (125 MHz, CF_3COOD) spectrum of compound **36b** (focusing on 124.0-165.0 ppm).

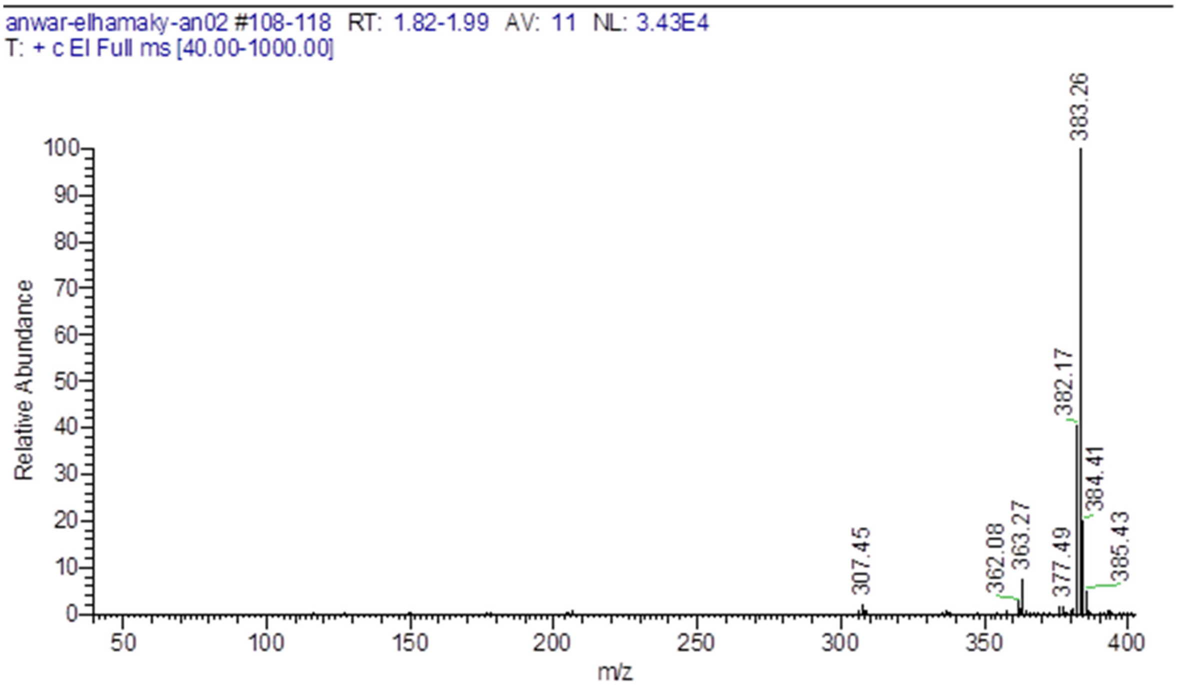

Figure S77. Mass spectrum of compound **36b**.

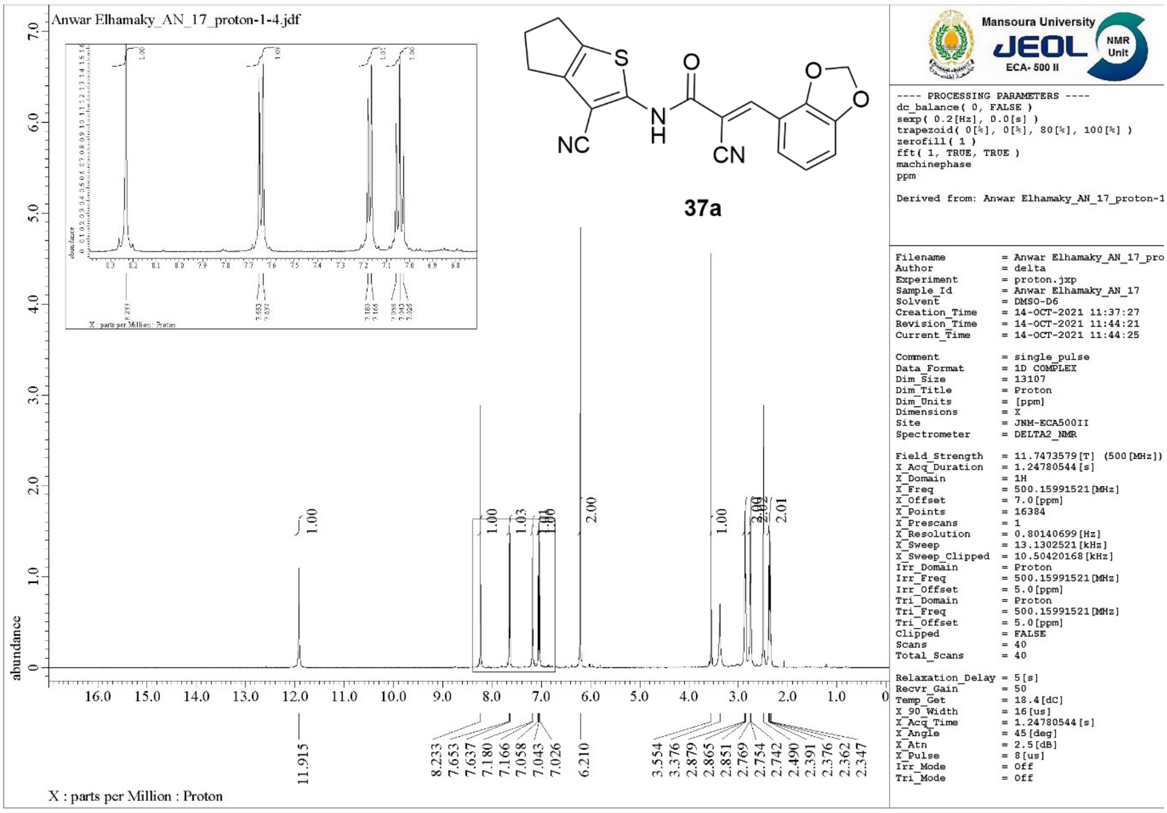

Figure S78. ^{1}H NMR (500 MHz, DMSO-d_6) spectrum of compound **37a** in *E* configuration.

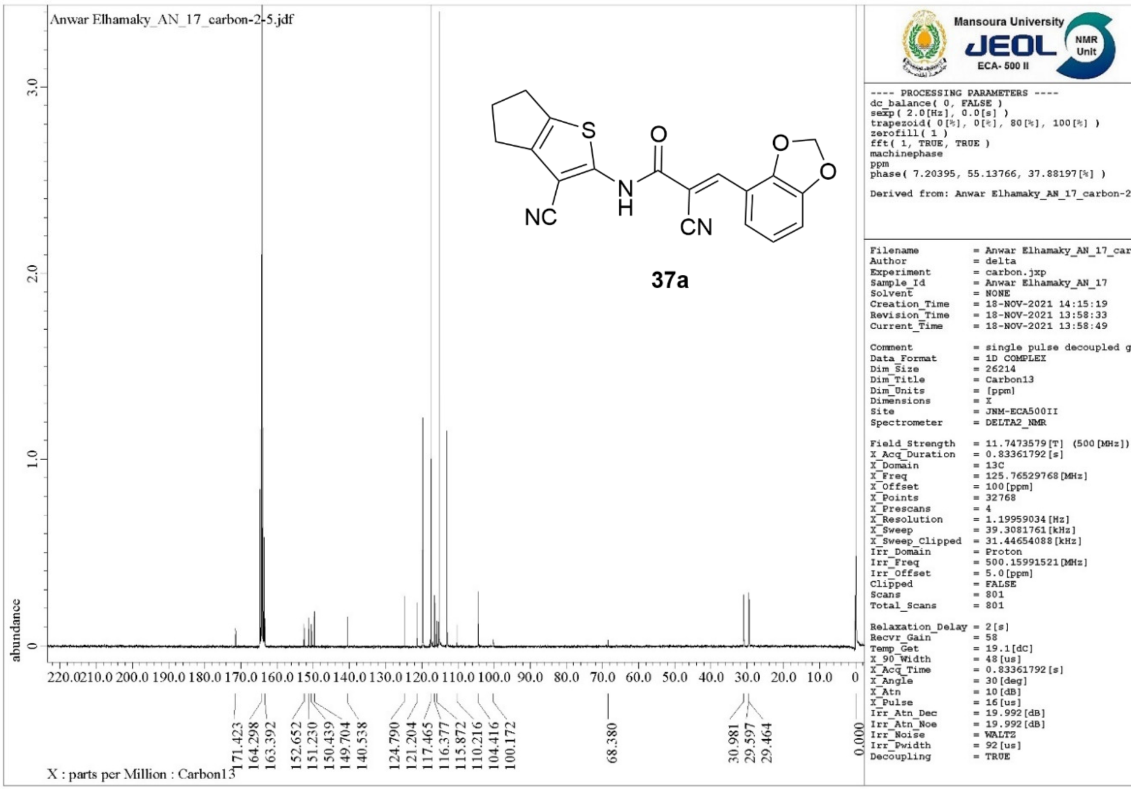

Figure S79 a. ^{13}C NMR (125 MHz, CF_3COOD) spectrum of compound **37a**.

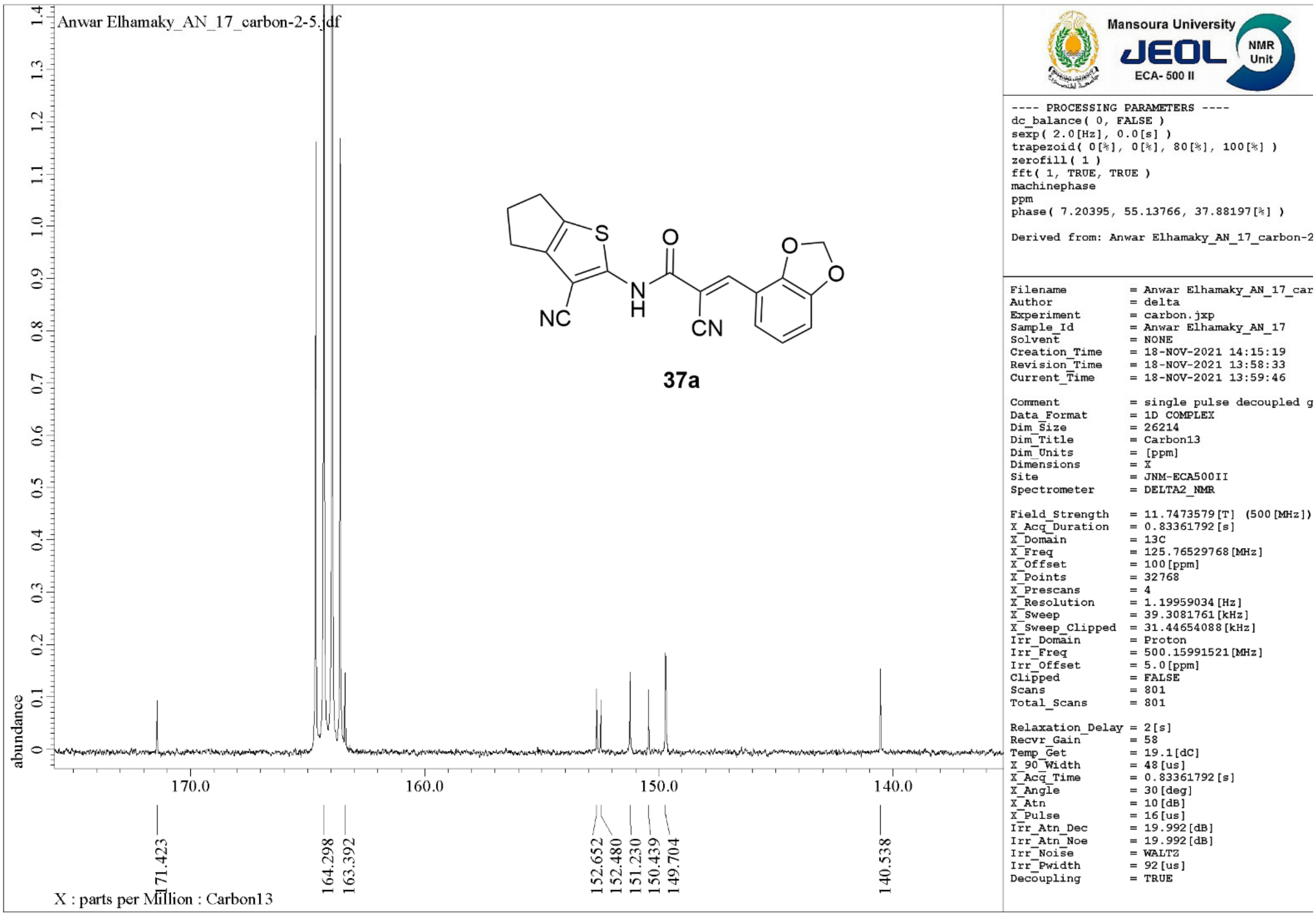

Figure S79 b. ^{13}C NMR (125 MHz, CF_3COOD) spectrum of compound **37a** (focusing on 140.0-172.0 ppm).

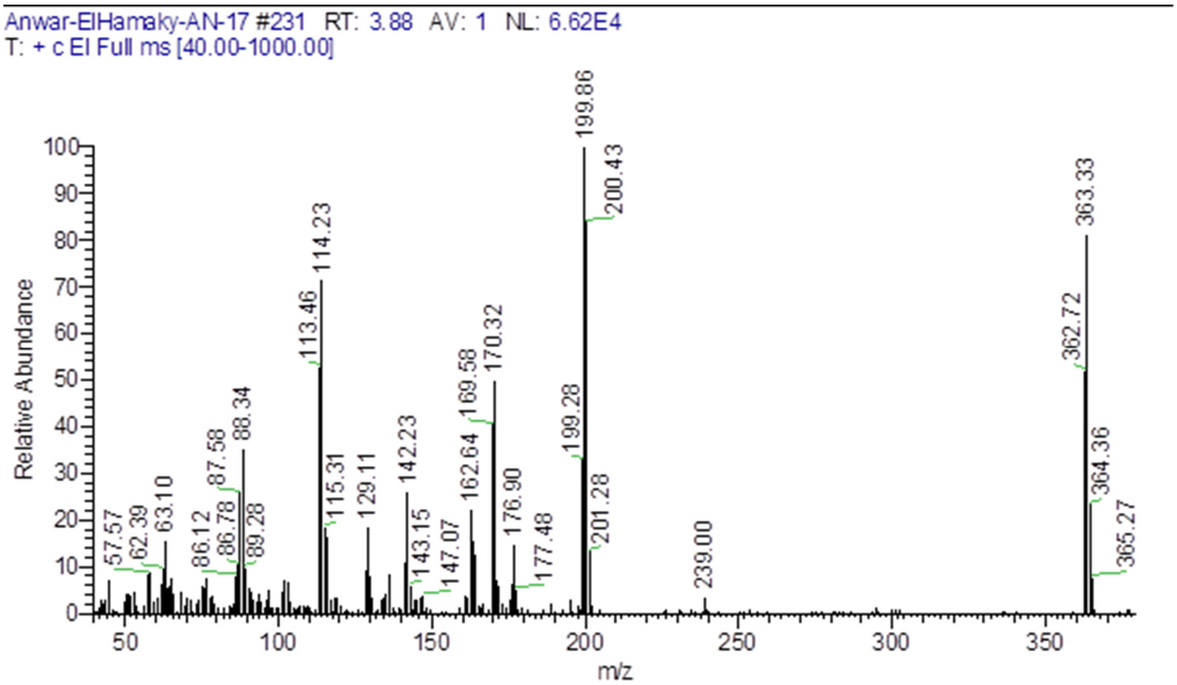

Figure S80. Mass spectrum of compound **37a**.

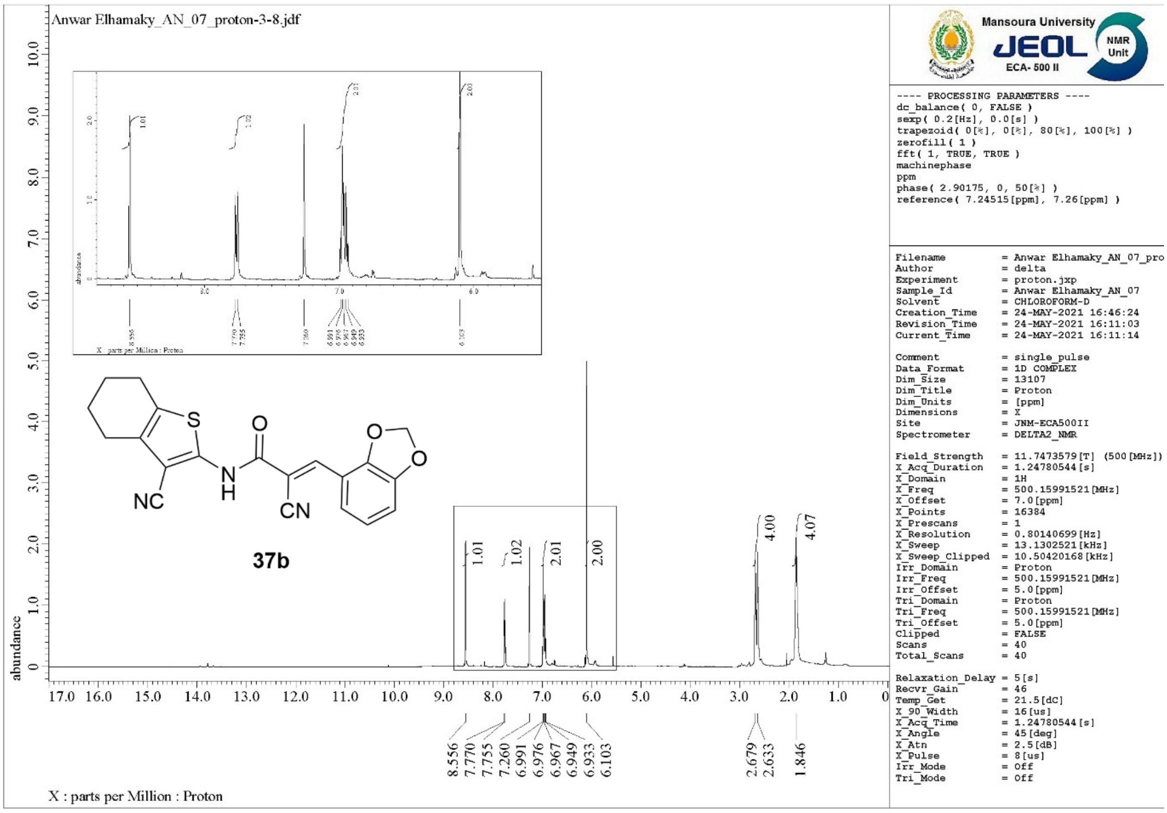

Figure S81. ^{1}H NMR (500 MHz, $CDCl_3$) spectrum of compound **37b** in *E* configuration.

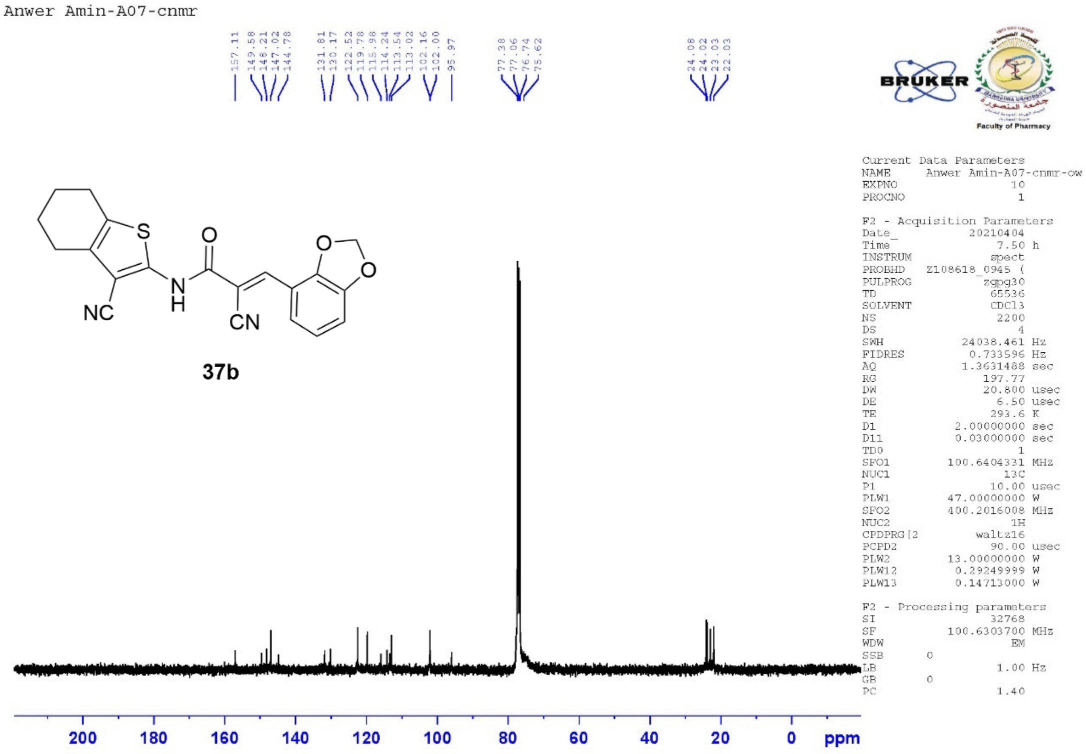

Figure S82. ^{13}C NMR (100 MHz, $CDCl_3$) spectrum of compound **37b**.

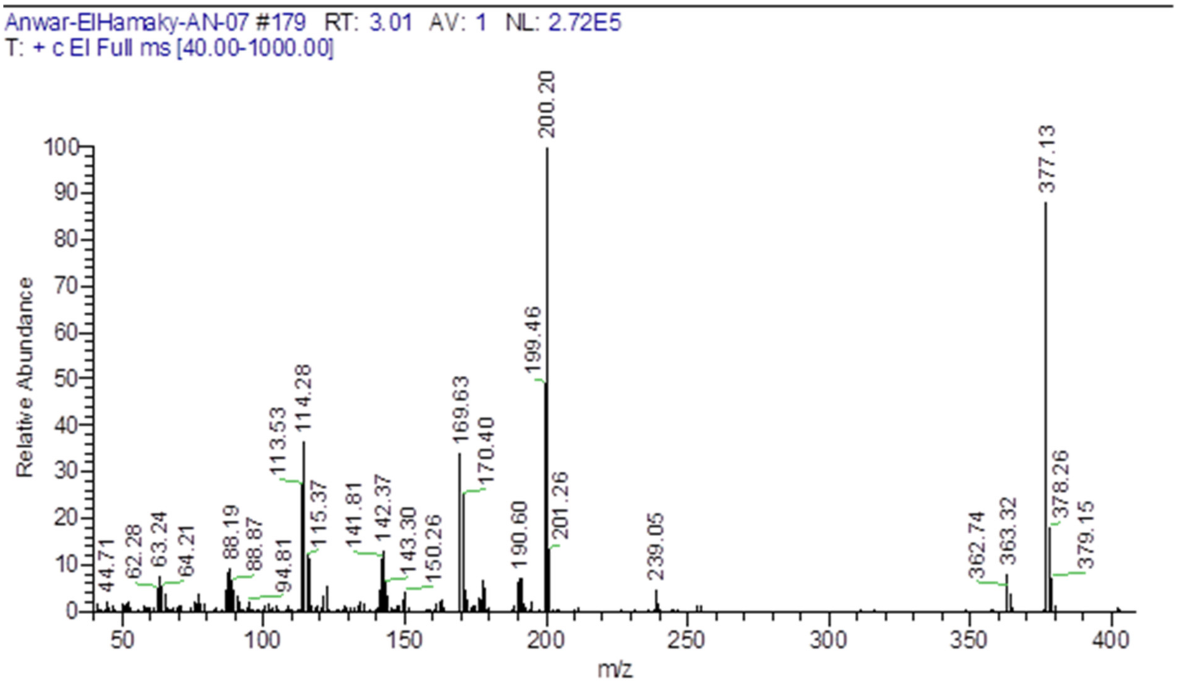

Figure S83. Mass spectrum of compound **37b**.

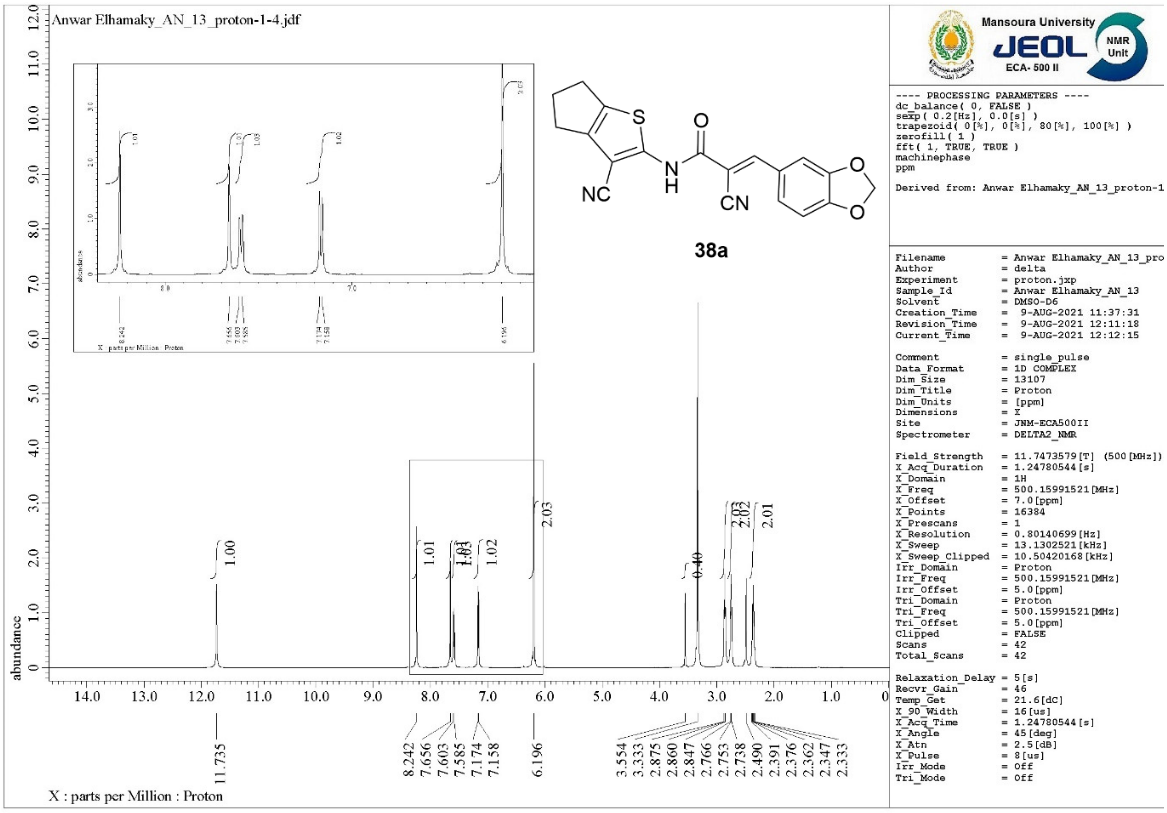

Figure S84. ^{1}H NMR (500 MHz, DMSO-d_6) spectrum of compound **38a** in *E* configuration.

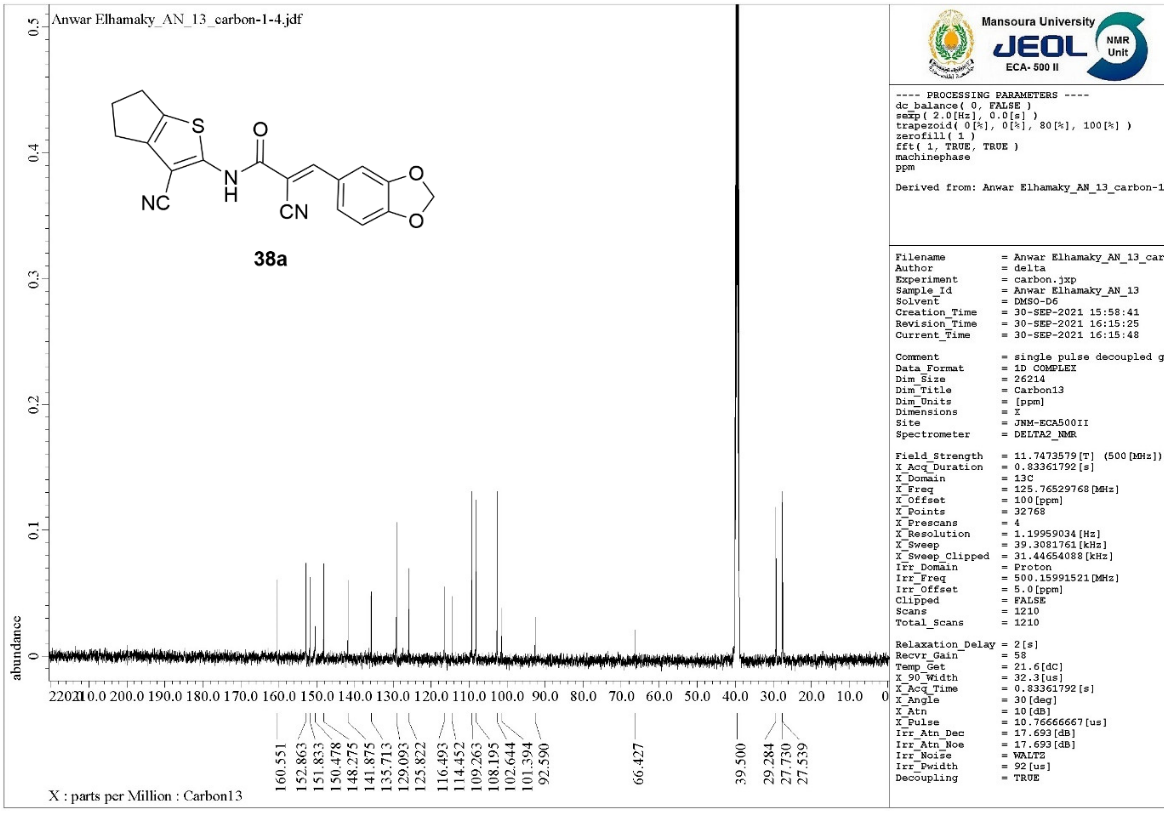

Figure S85. ^{13}C NMR (125 MHz, DMSO-d_6) spectrum of compound **38a**.

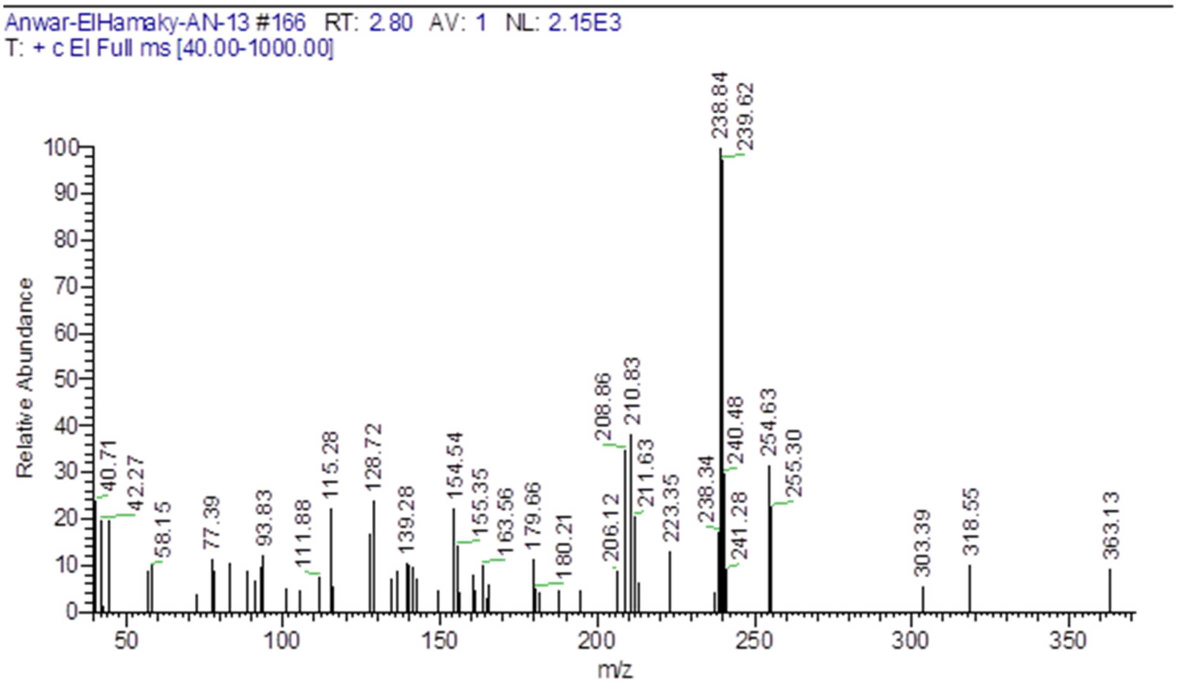

Figure S86. Mass spectrum of compound **38a**.

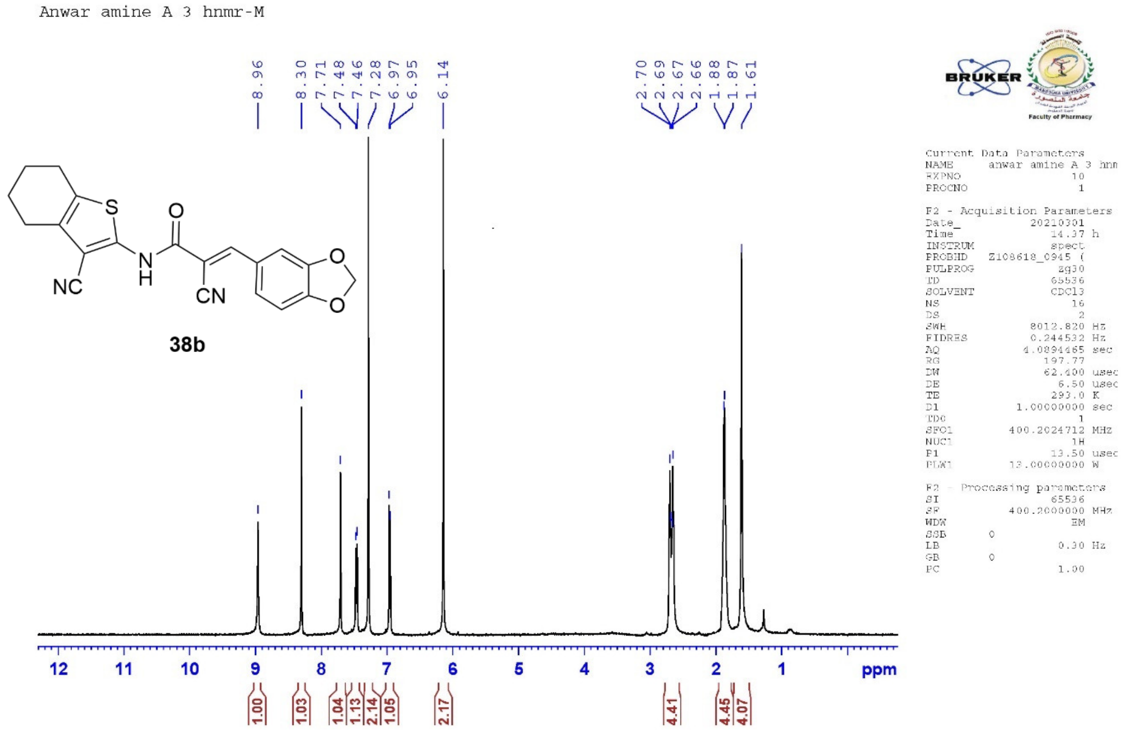

Figure S87. ^{1}H NMR (400 MHz, $CDCl_3$) spectrum of compound **38b** in *E* configuration.

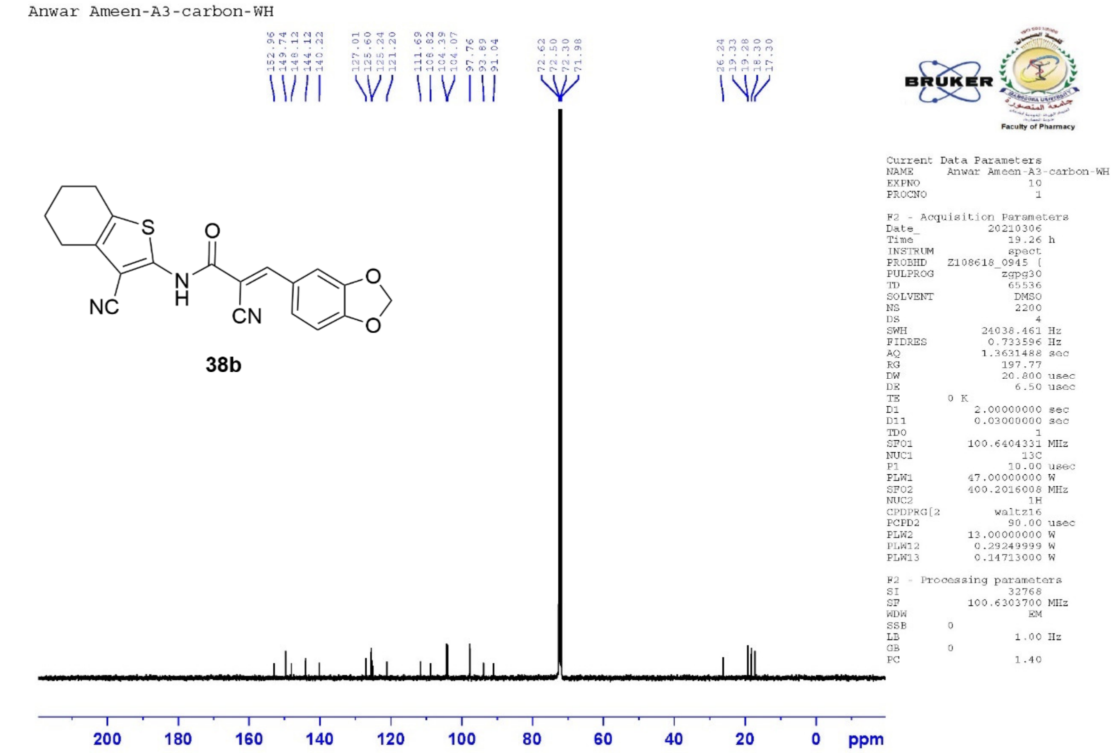

Figure S88. ^{13}C NMR (100 MHz, $CDCl_3$) spectrum of compound **38b**.

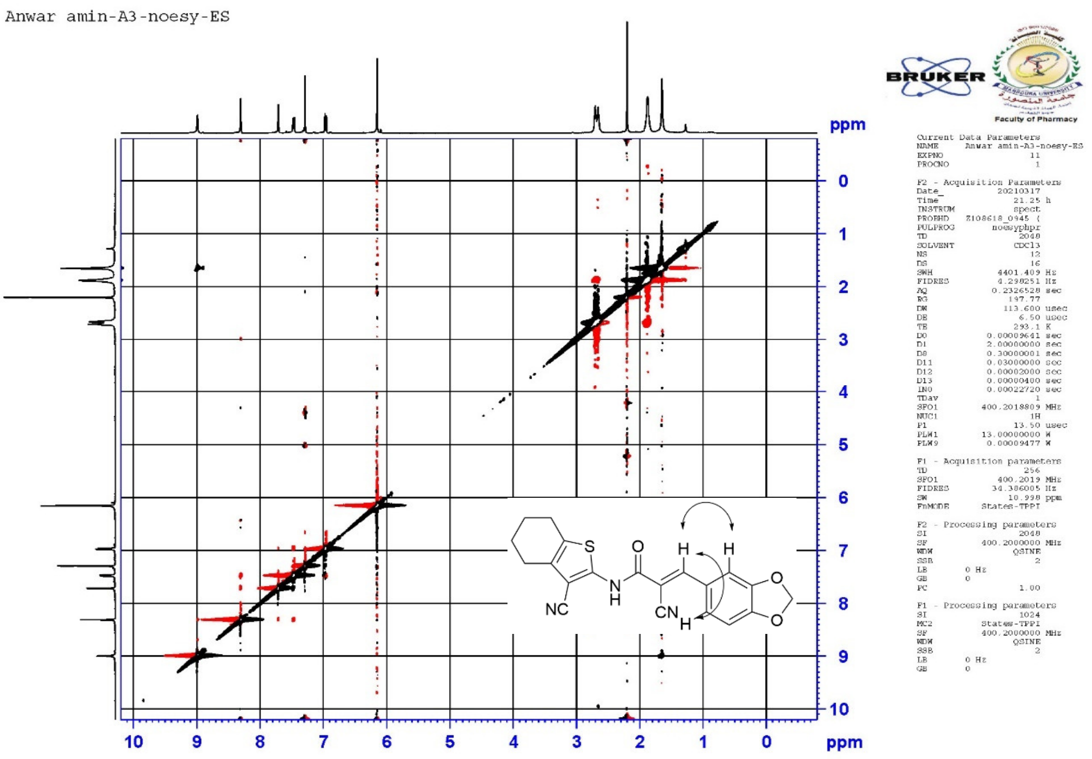

Figure S89. 2D NOESY NMR for compound **38b** in $CDCl_3$.

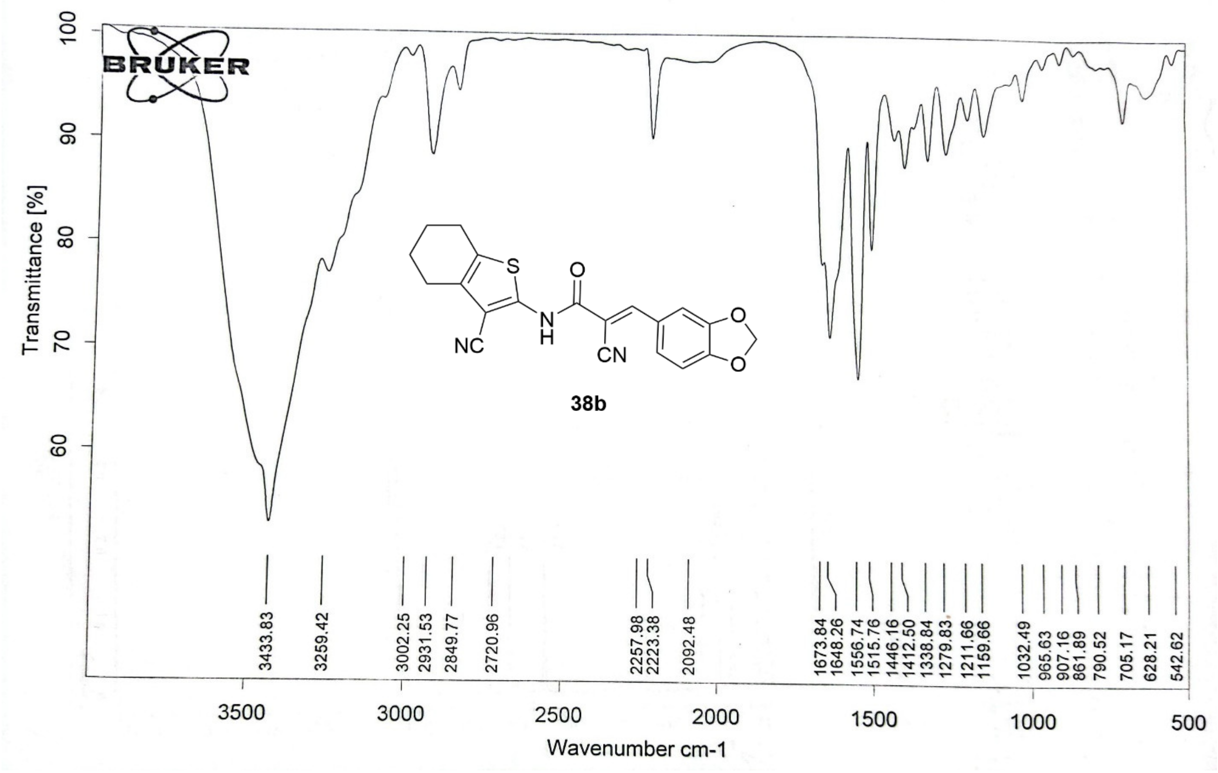

Figure S90. IR spectrum for compound **38b**.

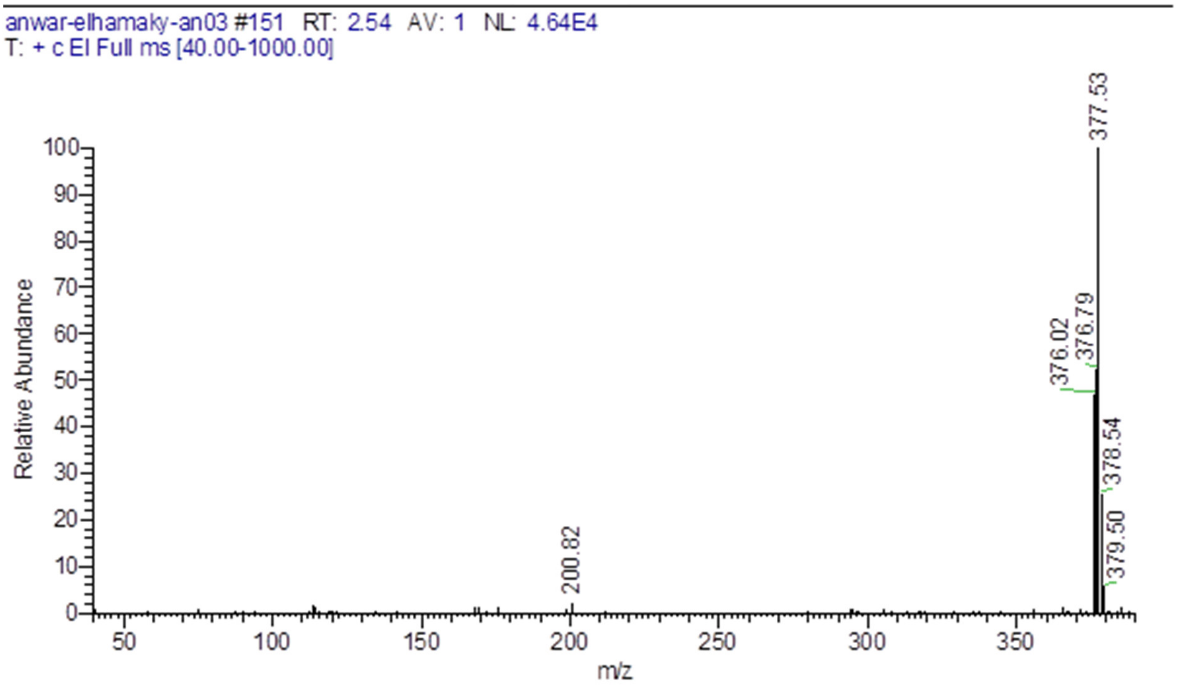

Figure S91. Mass spectrum of compound **38b**.

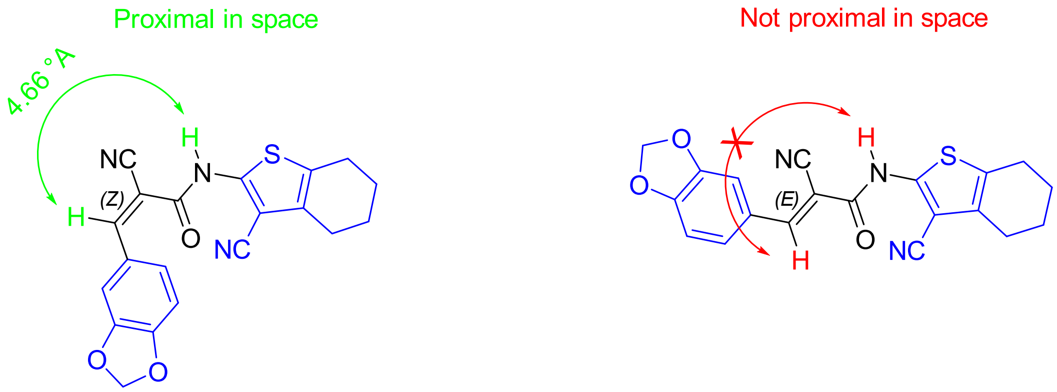

Figure S92. Spatial difference between vinyl and nitrogen protons in *Z* and *E* configurations of **38b**.

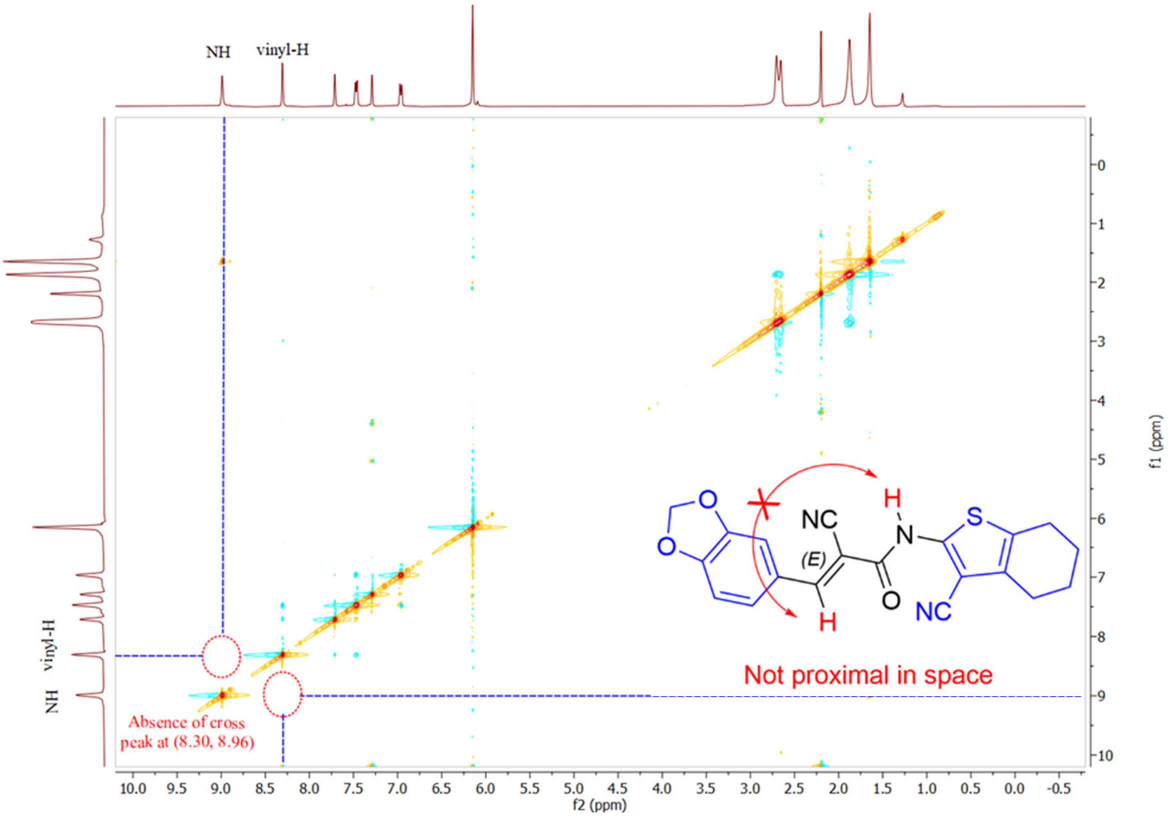

Figure S93. 2D NOESY NMR for compound **38b** in *E* configuration showing the absence of cross peak between vinyl and nitrogen protons.

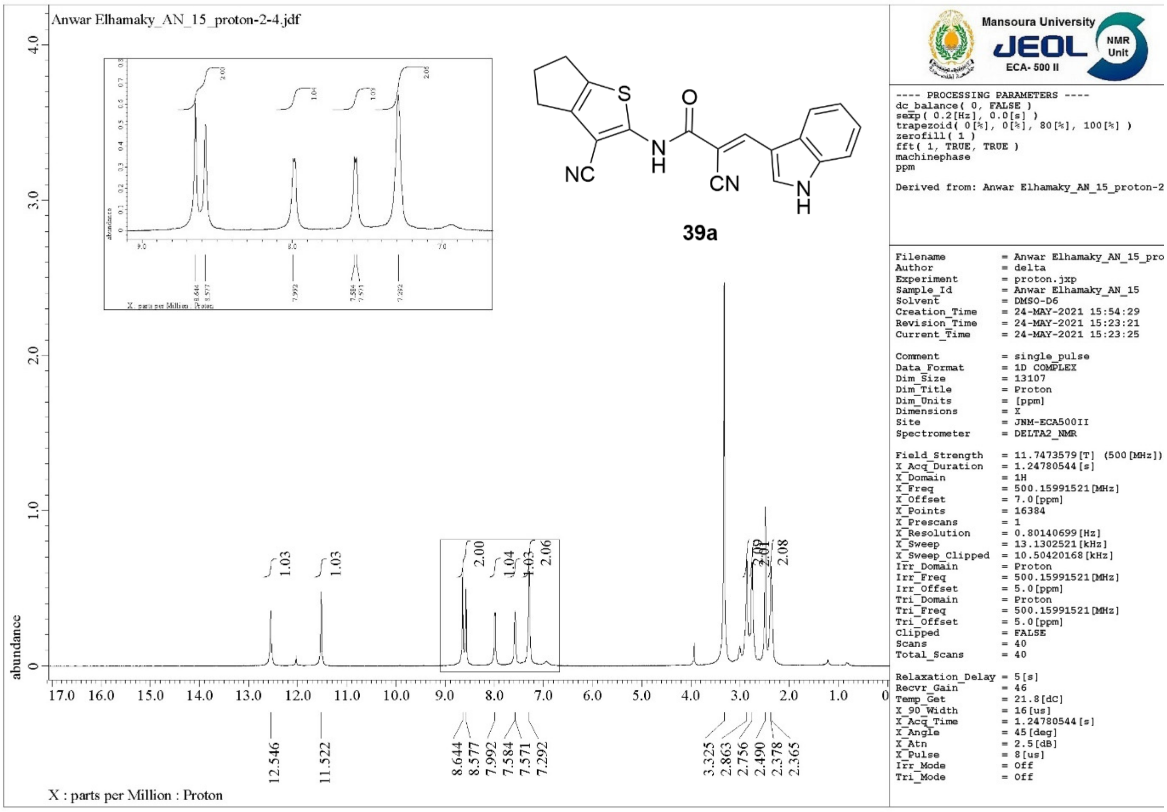

Figure S94. ^{1}H NMR (500 MHz, DMSO-d_6) spectrum of compound **39a** in *E* configuration.

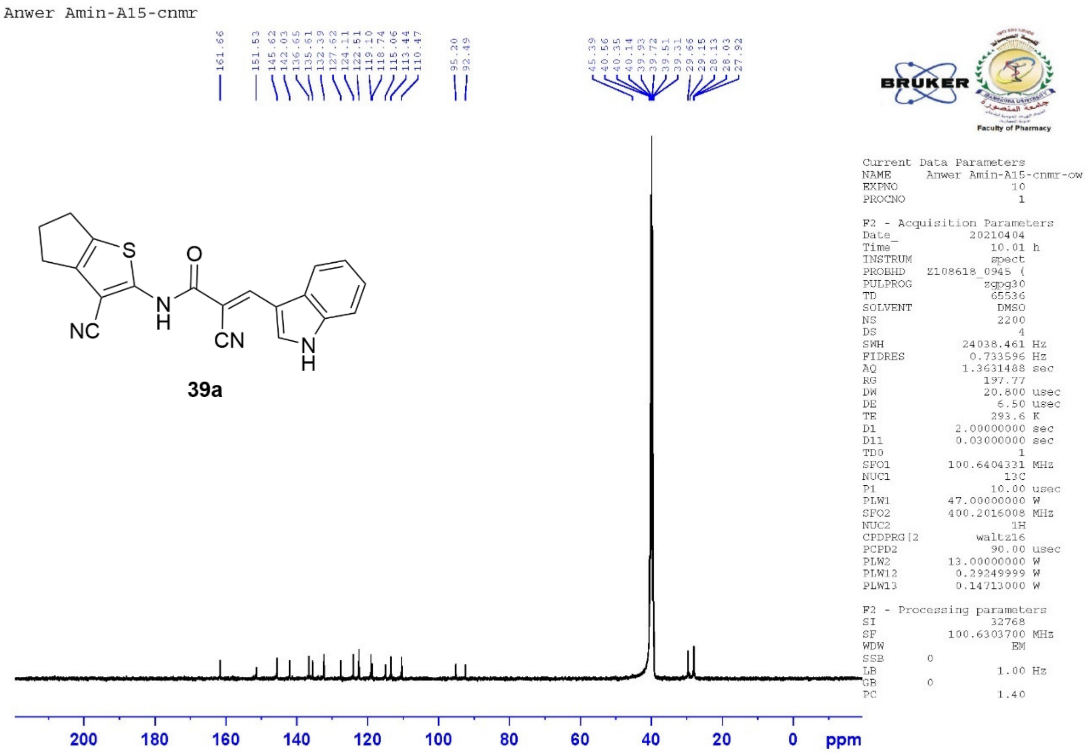

Figure S95. ^{13}C NMR (100 MHz, $CDCl_3$) spectrum of compound **39a**.

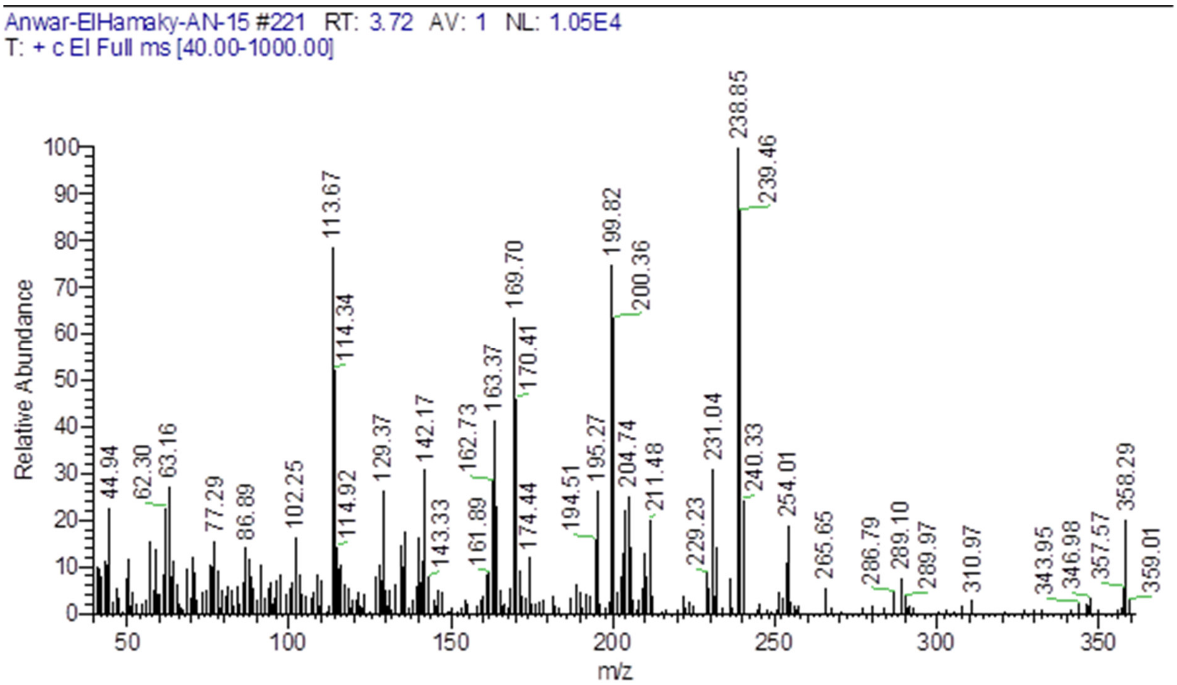

Figure S96. Mass spectrum of compound **39a**.

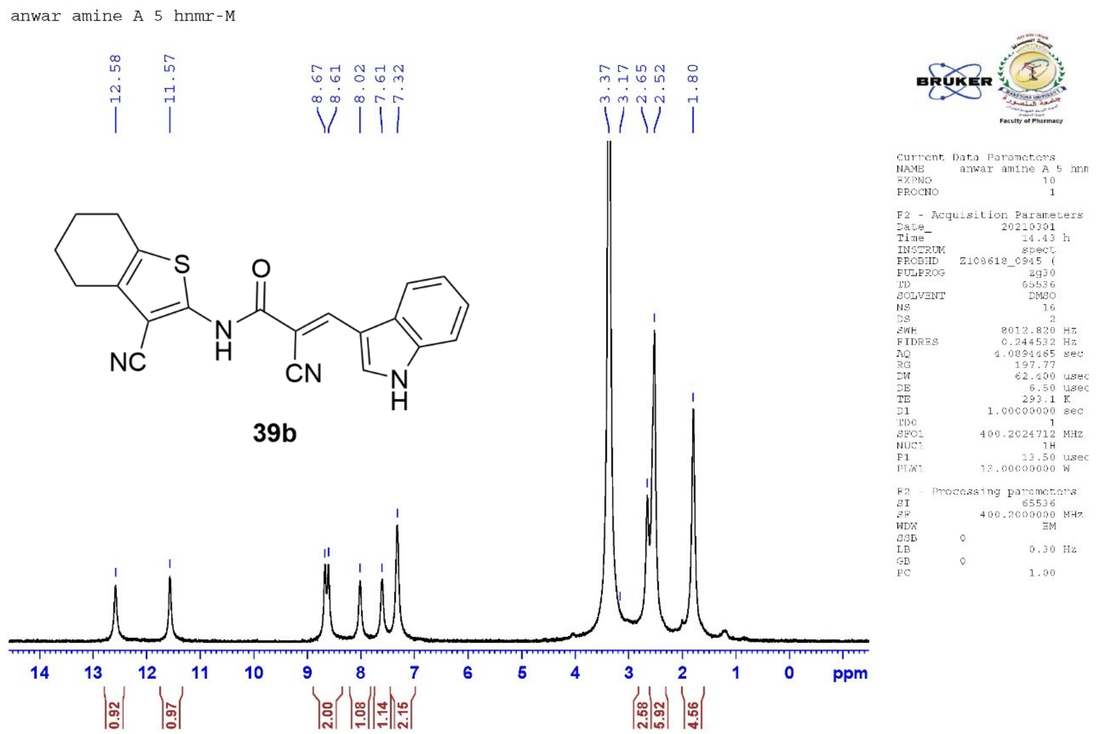

Figure S97. ^{1}H NMR (400 MHz, DMSO-d_6) spectrum of compound **39b** in *E* configuration.

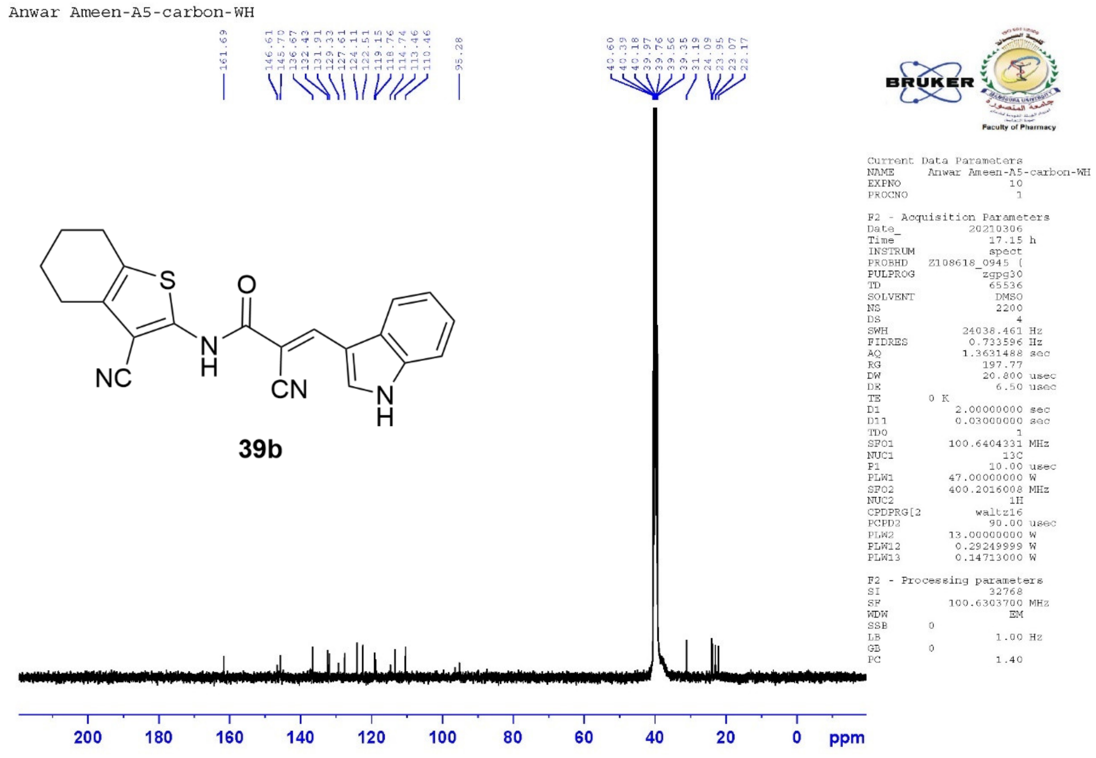

Figure S98. ^{13}C NMR (100 MHz, DMSO-d_6) spectrum of compound **39b**.

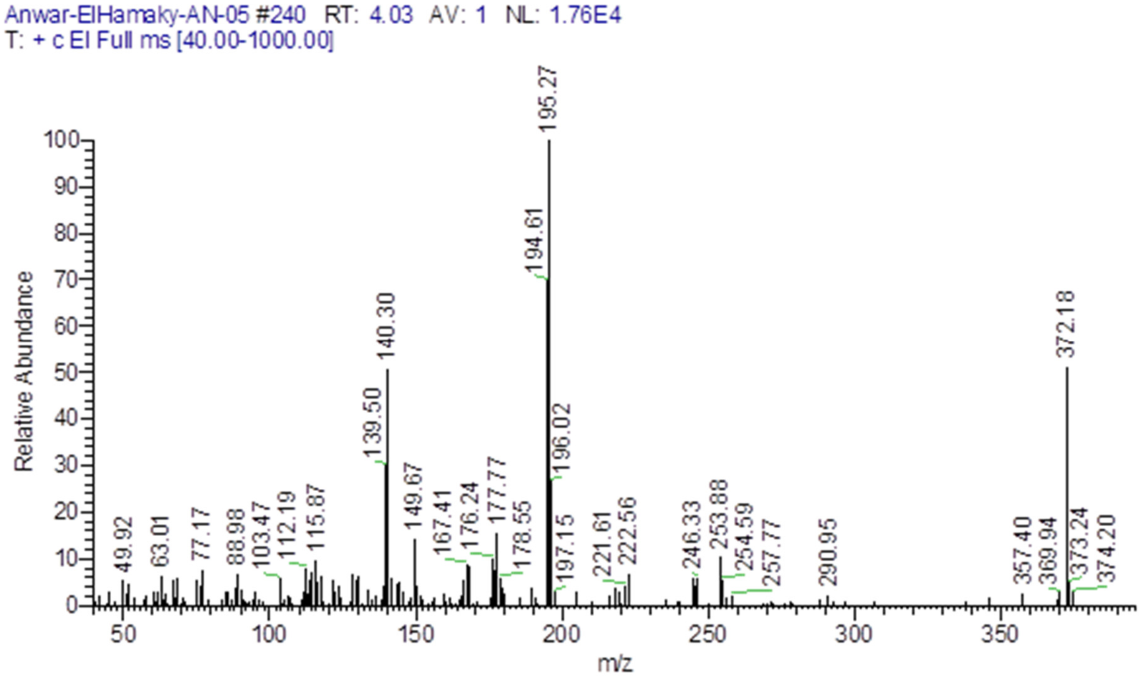

Figure S99. Mass spectrum of compound **39b**.

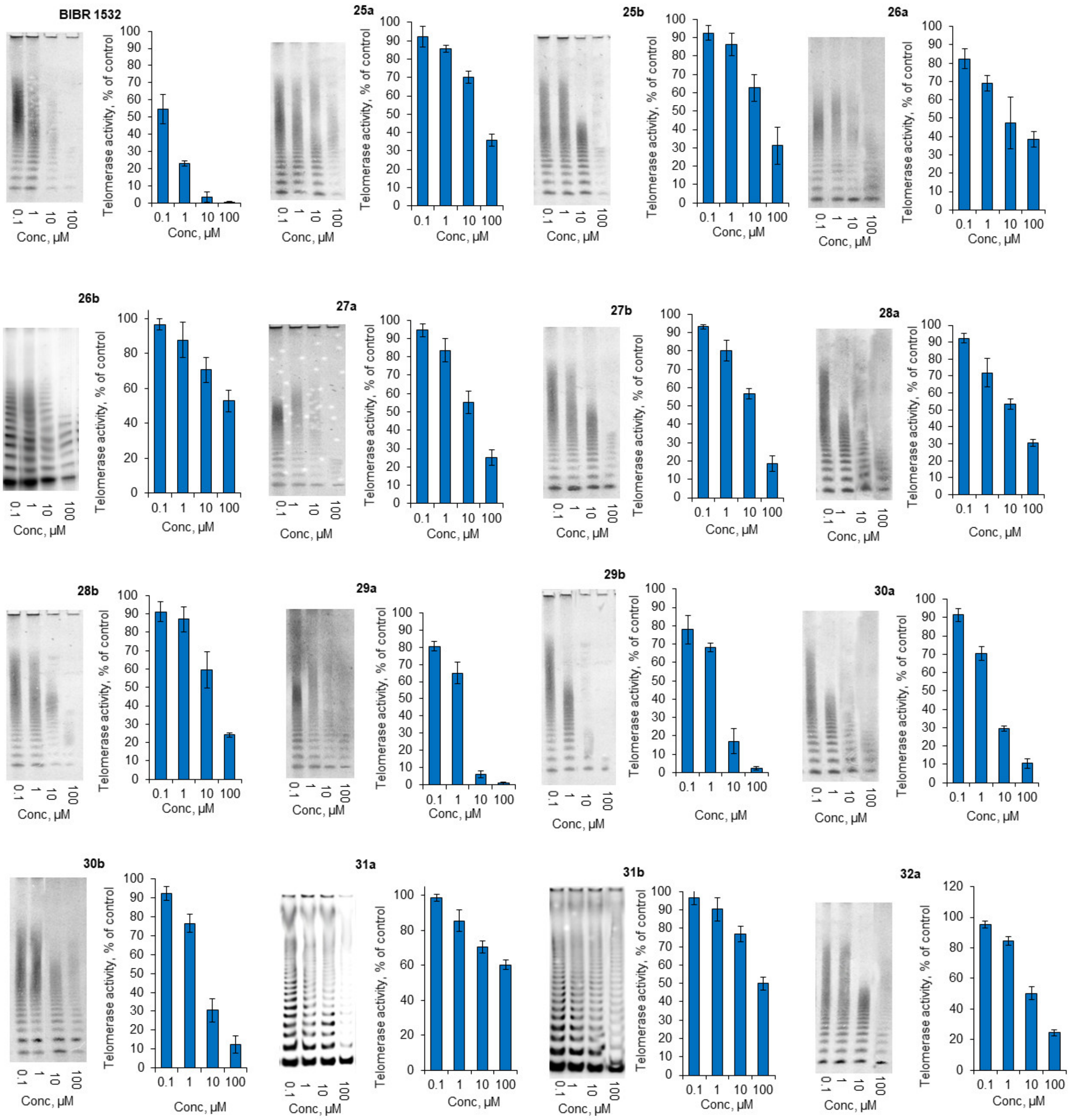

Figure S100. Suppression of telomerase activity in A549 cell-free lysates treated with different concentrations of inhibitors. Representative telomeric repeat amplification protocol (TRAP) gel electrophoresis for treated lysates and quantification of TRAP. One representative TRAP gel of a total of four for each of the experiments is shown. The results are presented as the mean ± standard error of the mean.

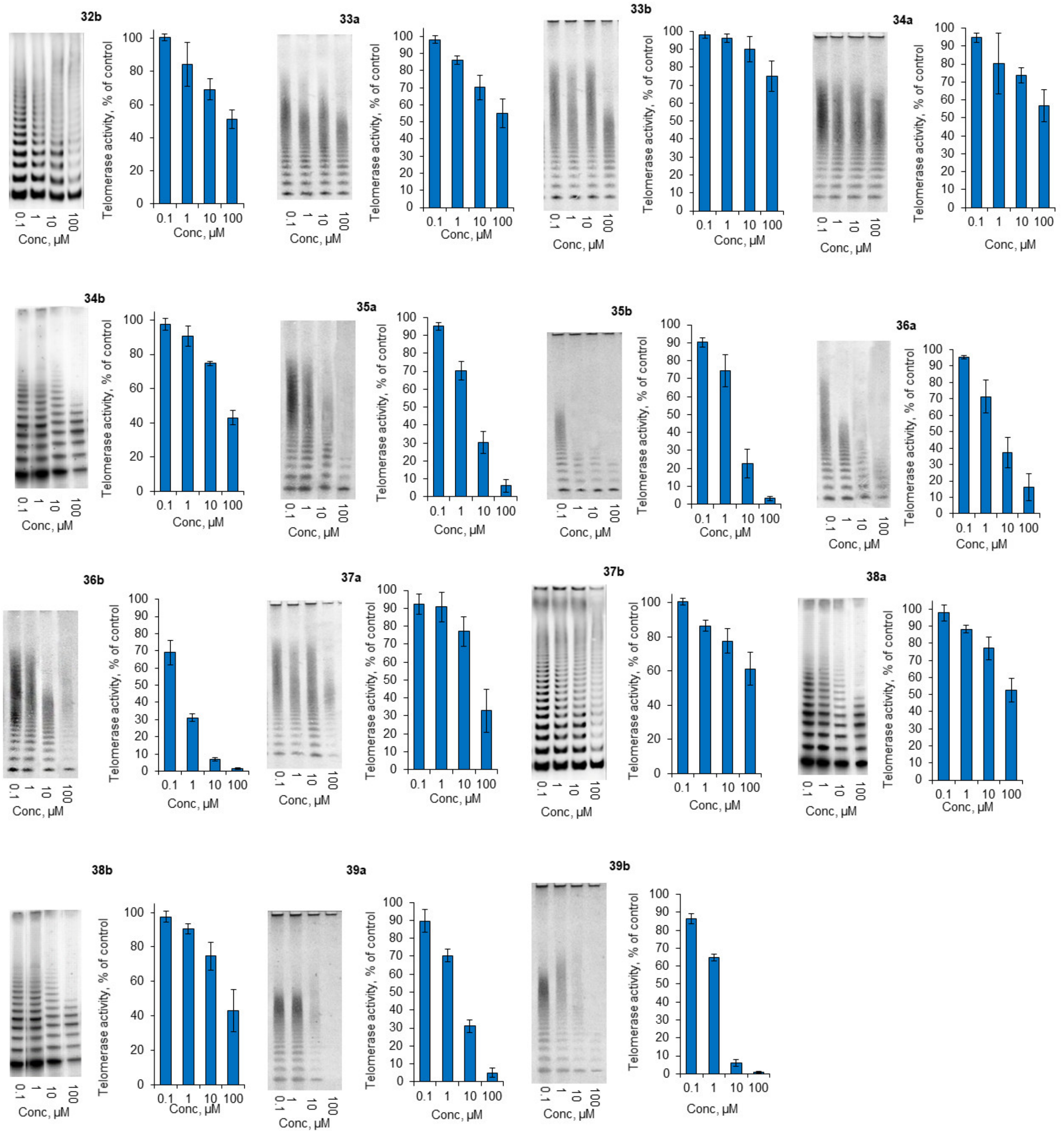

Figure S100. Suppression of telomerase activity in A549 cell-free lysates treated with different concentrations of inhibitors. Representative telomeric repeat amplification protocol (TRAP) gel electrophoresis for treated lysates and quantification of TRAP. One representative TRAP gel of a total of four for each of the experiments is shown. The results are presented as the mean ± standard error of the mean. (Continued).

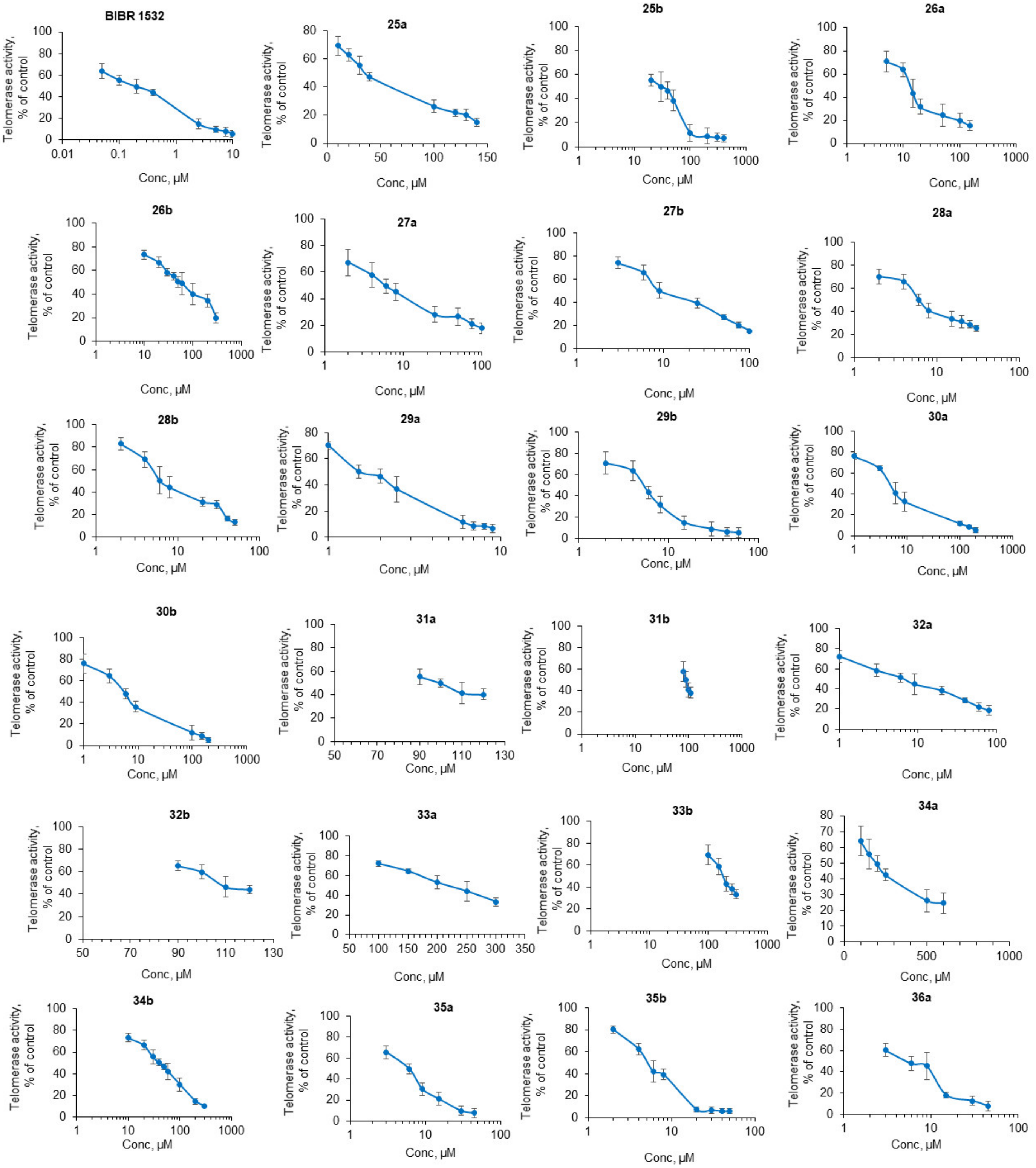

Figure S101. Dose-depending curves which were used for calculation of IC_{50} and IC_{90} values. N = 4. These data were obtained by RTQ-TRAP.

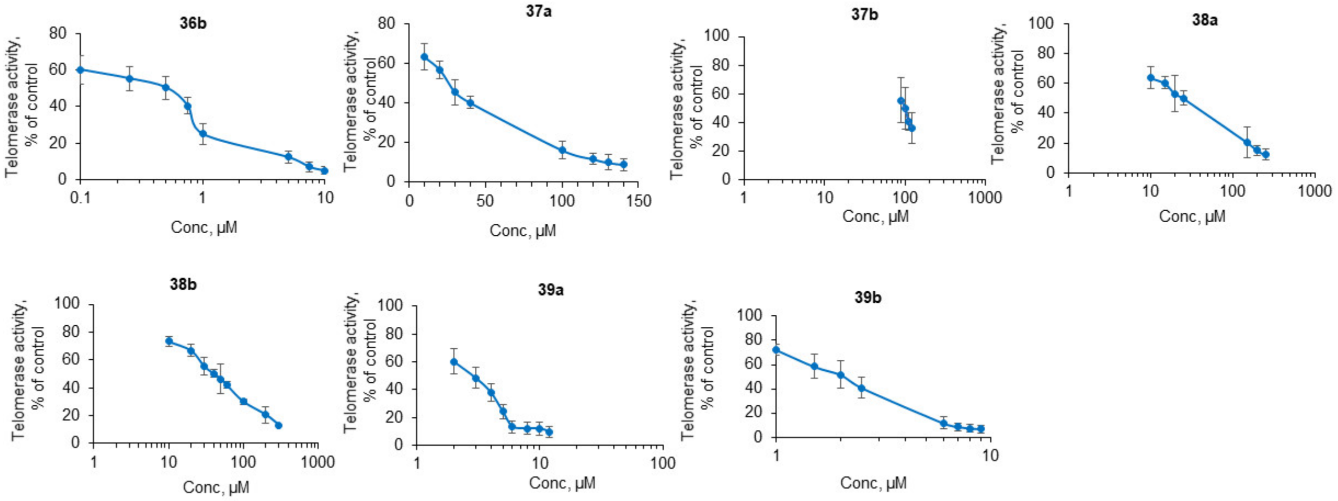

Figure S101. Dose-depending curves which were used for calculation of IC_{50} and IC_{90} values. N = 4. These data were obtained by RTQ-TRAP (Continued).

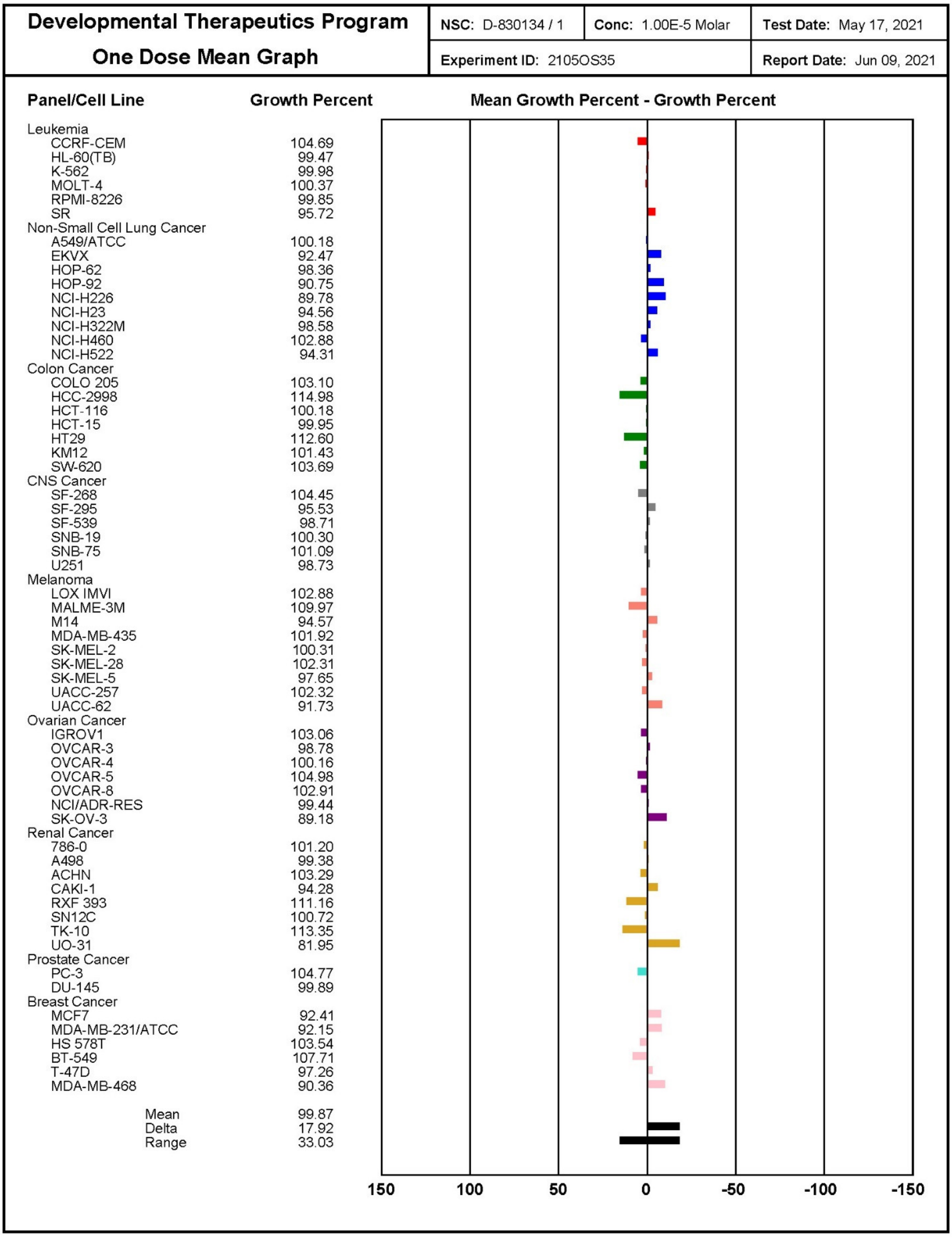

Figure S102. One dose mean graph for compound **36b** (NSC 830134) at 10 μM.
